# Friendly visiting by a volunteer for reducing loneliness or social isolation in older adults: A systematic review

**DOI:** 10.1002/cl2.1359

**Published:** 2023-11-30

**Authors:** Jorien Laermans, Hans Scheers, Philippe Vandekerckhove, Emmy De Buck

**Affiliations:** ^1^ Centre for Evidence‐Based Practice, Belgian Red Cross Mechelen Belgium; ^2^ Department of Public Health and Primary Care, Leuven Institute for Healthcare Policy KU Leuven Leuven Belgium; ^3^ Belgian Red Cross Mechelen Belgium; ^4^ Centre for Evidence‐Based Health Care Stellenbosch University Cape Town South Africa

## Abstract

**Background:**

Loneliness and social isolation are currently among the most challenging social issues. Given their detrimental impact on physical and mental health, identifying feasible and sustainable interventions to alleviate them is highly important. Friendly visiting, a befriending intervention whereby older persons are matched with someone who visits them on a regular basis, seems promising. However, it is unclear if face‐to‐face (F2F) friendly visiting by a volunteer (FVV) is effective at reducing loneliness or social isolation, or both.

**Objectives:**

To assess the effect of F2F FVV on feelings of loneliness, social isolation (primary outcomes) and wellbeing (i.e., life satisfaction, depressive symptom experiencing and mental health; secondary outcomes) in older adults.

**Search Methods:**

We searched six electronic databases up until 11 August 2021. We also consulted 15 other resources, including grey literature sources and websites of organizations devoted to loneliness and ageing, between 25 October and 29 November 2021.

**Selection Criteria:**

We included experimental and observational studies that quantitatively measured the effect of F2F FVV, compared to no friendly visiting, on at least one of following outcomes in older adults (≥60 years of age): loneliness, social isolation or wellbeing.

**Data Collection and Analysis:**

Two reviewers independently performed study selection, data extraction and synthesis, risk of bias and GRADE assessment. If outcomes were measured multiple times, we extracted data for one short‐term (≤1 month after the intervention had ended), one intermediate‐term (>1 and ≤6 months), and one long‐term time point (>6 months). Data from randomized controlled trials (RCTs) and non‐RCTs were presented and synthesized separately. Synthesis was done using vote counting based on the direction of effect.

**Main Results:**

Nine RCTs and four non‐RCTs, conducted primarily in the United States and involving a total of 470 older adults (mean or median ages: 72–83 years), were included. All studies were limited in size (20–88 participants each). Programmes lasted 6–12 weeks and mostly involved weekly visits by undergraduate students to community‐dwelling older adults. Visits consisted mainly of casual conversation, but sometimes involved gameplaying and TV‐watching. All studies had major shortcomings in design and execution. The current evidence about the effect of F2F FVV on loneliness in older adults is very uncertain, both in the short (one RCT in 88, and one non‐RCT in 35 participants) and intermediate term (one RCT in 86 participants) (both very low‐certainty evidence). The same goes for the effects on social isolation, again both in the short (one RCT in 88, and two non‐RCTs in 46 participants) and intermediate term (two non‐RCTs in 99 participants) (both very low‐certainty evidence). Similarly, there is a lot of uncertainty about the effect of F2F FVV on outcomes related to wellbeing (all very low‐certainty evidence).

**Authors’ Conclusions:**

Due to the very low‐certainty evidence, we are unsure about the effectiveness of F2F FVV with regard to improving loneliness, social isolation, or wellbeing in older adults. Decision‐makers considering implementing FVV should take into account this uncertainty. More and larger high‐quality studies that are better designed and executed, and preferably conducted in various settings, are needed.

## 1 PLAIN LANGUAGE SUMMARY

### 1.1 Research evidence on the effectiveness of face‐to‐face visiting by a volunteer for improving social isolation and loneliness of older adults is very uncertain

We are unsure if friendly face‐to‐face visits by a volunteer can improve loneliness, social isolation, depressive feelings, life satisfaction and mental health‐related outcomes in older adults. Decisionmakers who are considering friendly face‐to‐face visiting as a way to alleviate loneliness or social isolation in older adults should take this research uncertainty into account.

### 1.2 What is this review about?

Loneliness and social isolation are reaching epidemic proportions in both children and adults, despite the increasing connectedness in our 21st century world. Given their devastating impact on physical and mental health, it is important to identify and invest in feasible and sustainable options to decrease social isolation and feelings of loneliness.

Friendly face‐to‐face visiting, where people are matched to someone who visits them in‐person on a regular basis, seems to be a realistic and sustainable option for providing social support.

**What is the aim of this review?**

We wanted to find out if friendly face‐to‐face visiting by a volunteer is effective at reducing loneliness or social isolation, or both, in adults aged 60 or older. We also wanted to find out if visits can improve depressive symptoms, life satisfaction and mental health outcomes.

### 1.3 What studies are included?

We found 13 relevant studies comparing friendly visiting by a volunteer to no friendly visiting, involving 470 older adults in total. Ten of these studies were conducted in the USA.

Friendly face‐to‐face visiting programmes ranged from six to 12 weeks in duration and mostly involved weekly visits by undergraduate students. Visits consisted mainly of casual conversation, but playing games and watching TV were also mentioned.

### 1.4 What are the main findings of this review?

The evidence is very uncertain about the effect of friendly face‐to‐face visiting by a volunteer on improving loneliness, social isolation, depressive symptom experiencing, life satisfaction and mental health‐related outcomes in older adults.

None of the studies reported on the long‐term effects (more than six months after the friendly visiting programme has ended) on loneliness or social isolation. None of the studies reported on the medium‐term effects (1‐6 months after the programme has ended) on mental health.

### 1.5 What do the findings of this review mean?

We have very little confidence in the evidence, because the studies were very small, used methods likely to lead to errors in their results, and often did not transparently report all data. Given the limitations of the available evidence, further research is very likely to change the results of our review.

### 1.6 How up to date is this review?

The authors searched for studies up to August 2021.

## 2 BACKGROUND

### 2.1 Description of the condition

The concepts of ‘loneliness’ and ‘social isolation’ have been debated and contested extensively, resulting in myriad definitions. In addition, these terms are often used interchangeably, although they are distinct (though related) concepts. Therefore, defining these concepts and highlighting the distinctions between them is of the essence.

In this systematic review, loneliness is defined as ‘a subjective, unwelcome feeling of lack or loss of companionship. It happens when we have a mismatch between the quantity and quality of social relationships that we have, and those that we want’ (the cognitive deficit model of Perlman, 1981). It is therefore a deeply personal and subjective negative experience.

In contrast, social isolation is an objective state, defined in terms of the quantity of social relationships and contacts. It reflects a reduction in social network size and paucity of social contact, which can be triggered by factors such as mobility impairments, unemployment, or deteriorating health (Steptoe, 2013).

Feeling lonely is therefore different from being socially isolated. In fact, a person may feel lonely even in the presence of other people. Similarly, an individual may live alone without feeling lonely.

Although we live in an increasingly connected world, millions of children and adults suffer from loneliness or social isolation, or both. The Joint Research Centre of the European Commission reported in a 2018 policy brief that 7% of adults in Europe (roughly 30 million people) frequently feel lonely, and 18% (around 75 million people) are socially isolated (i.e., meet socially with friends, relatives or work colleagues at most once a month) (d'Hombres, 2018). A cross‐country survey of adults in the United States, the United Kingdom and Japan, performed by the Kaiser Family Foundation in partnership with The Economist, revealed that prevalence rates of loneliness or social isolation lie as high as 22% (US), 23% (UK) and 9% (Japan) (DiJulio, 2018).

Although these estimated prevalence rates themselves have remained stable over the past decades, the burden of loneliness and social isolation is expected to increase even further during the next couple of decades. Population ageing is one of the key contributors: as people grow older, they are at increased risk of living by themselves and of becoming disabled, which in its turn constitutes a barrier to social interaction. In its 2015 evidence review, Age UK stated that 6%–10% of older people say they always or often feel lonely, and that nearly half of the people over 65 say that television or pets are their main form of company (Davidson, 2015).

An increasing number of studies show that loneliness and social isolation can have a detrimental impact on physical and mental health. For instance, they reportedly have the same harmful effect as smoking 15 cigarettes a day (Holt‐Lunstad, 2010), and put individuals at greater risk of developing clinical dementia (Holwerda, 2014). In addition, loneliness has been associated with negative psychological effects such as depressed mood, low levels of life satisfaction and happiness (Prince, 1997; Schultz, 1984). These findings highlight the need for effective interventions to tackle loneliness and social isolation.

A growing range of interventions are being developed to alleviate social isolation and loneliness. These include social facilitation interventions (e.g., friendship clubs, shared interest topic groups), psychological therapies (whereby recognized therapeutic approaches are delivered by trained professionals, e.g., mindfulness, reminiscence therapy), health and social care provision (whereby health and/or social care professionals are involved and participants are enroled in a formal care programme, either in a nursing home or in the community setting), animal interventions (e.g., animal‐assisted therapy, robotic pets), befriending interventions (a form of social facilitation with the aim of formulating new friendships) and leisure/skill development interventions (e.g., gardening programmes, voluntary work, computer training courses) (reviewed by Gardiner, 2018).

Among the different existing interventions, friendly visiting, a befriending intervention whereby older persons are matched with someone who visits them on a regular basis, seems to be a realistic and sustainable option for providing social support. However, until this day, it remains unclear if friendly visiting by a volunteer is effective at reducing loneliness or social isolation, or both.

### 2.2 Description of the intervention

The intervention of interest for this review is any frequency and any duration of friendly visiting by a volunteer (of any age) to a community‐dwelling or institutionalized older adult. We define a volunteer as a person who does something, especially helping other people, willingly and without being forced or paid to do it; either in a formal or informal setting. During these visits, the volunteer engages in friendly talking, playing games and/or reminiscing, with the sole purpose of reducing loneliness, social isolation, depressive symptoms, and/or improving life satisfaction and/or mental health in the older adult.

### 2.3 How the intervention might work

The Model of Depression and Loneliness (MODEL) theoretical framework may offer some insight in how friendly visiting might decrease social isolation and loneliness (Cohen‐Mansfield, 2007). Rooted in a cognitive‐behavioural theory, MODEL describes the influence of environmental resources, health, stressful life events and psychological factors on loneliness and depression in older adults. According to the framework, older adults experience less opportunities to meet people, may live alone more often and may face limitations in financial resources, making it harder to create new and maintain existing social contacts. Besides these environmental factors that reflect social isolation, health issues and difficulties with mobility represent additional barriers to developing meaningful social ties. Stressful life events such as retirement, deaths of friends and family, and relocation can cause people to lose long‐standing social networks, thereby contributing to loneliness. Finally, long‐standing reliance on established contacts, little need to initiate new contacts, and decreased social skills may affect their ability to engage in meaningful social relationships.

The MODEL framework was shown to explain 42% of the variance in loneliness and 47% of the variance in depressed affect among low‐income older adults (Cohen‐Mansfield, 2007), highlighting the importance of developing interventions targeting the modifiable factors that contribute to loneliness, including new contacts, mobility and financial resources.

Friendly visiting programmes are capable of overcoming most of these barriers, as they allow the older adults to meet and maintain social contact with volunteers, regardless of whether they experience any mobility or financial issues. As loneliness has been associated with depressed mood, low levels of life satisfaction and happiness (Prince, 1997; Schultz, 1984), friendly visiting may exert its beneficial effect on these outcomes through that on loneliness.

### 2.4 Why it is important to do this review

Several existing systematic reviews have looked at the effectiveness of interventions aiming to reduce loneliness or social isolation, but either applied a very broad or a rather narrow scope.

In 2017, a systematic review and meta‐analysis was published investigating the effectiveness of befriending interventions targeting individuals with distressing physical and mental conditions (Siette, 2017). This review included a wide range of befriending interventions (social support delivery through face‐to‐face encounters at home, in support groups, or via telephone contact) in a very diverse population of interest (adults of any age with any type of physical or mental condition).

Similarly, another systematic review on the effectiveness of health promotion interventions that target social isolation and loneliness among older people, used a broad scope for its interventions of interest (Cattan, 2005). Studies were categorized as ‘group’, ‘one‐to‐one’, ‘service provision’ and ‘community development’ interventions. The ‘one‐to‐one’ category included a wide range of interventions, including home visits by professionals providing health assessments or services, telephone support‐therapy by social services, friendly telephone calls by peers, and social support visits by volunteers.

During the development of the 2015 evidence‐based guideline ‘Older people: independence and mental wellbeing’ by the National Institute for Health and Care Excellence (NICE, 2015), another very broadly scoped systematic review was developed to investigate the effectiveness of interventions to improve or protect the mental wellbeing and/or independence of older people in the United Kingdom (McDaid, 2015).

Similarly, a recently published integrative review included a wide range of interventions to reduce social isolation and loneliness among older people (Gardiner, 2018).

Also in 2018, the What Works Centre for Wellbeing published an overview of 14 systematic reviews of controlled studies published between 2008 and 2018 looking into the effectiveness of interventions aimed to alleviate loneliness (Victor, 2018). Again, the included studies investigated an extremely diverse range of interventions, delivered either in the community setting or in care homes and residential facilities.

Despite their broad scopes, none of these existing (overviews of) systematic reviews have allowed to make clear statements on the effectiveness of friendly face‐to‐face visiting by a volunteer to the generalizable older population, that is, older adults that do not suffer from any serious physical or mental illness.

Several other systematic reviews have narrowed the scope of their studied population to adults suffering from chronic non‐cancer pain (Cooper, 2014) or older adults who experienced a fall (Tricco, 2022), only looked at interventions delivered by health or social care professionals (Grant, 2014; Montgomery, 2008; Sims‐Gould, 2017), or did not investigate the effect of friendly visiting (Franck, 2016; Snowden, 2015). Another recent systematic review focused on the effectiveness of 20 interventions used to combat social isolation, but not loneliness, in older adults (Manjunath, 2021).

In conclusion, the existing systematic reviews highlight the need for a systematic collection, extraction and analysis of studies looking specifically at the effectiveness of friendly visiting by a volunteer on reducing loneliness or social isolation in older, otherwise healthy, adults. In addition, in their overview of reviews, Victor et al. highlighted the need for better reporting of numerical data and a focus on effect sizes and precision rather than using p values as a surrogate for effectiveness, in both future trials and reviews (Victor, 2018).

Loneliness and social isolation are proving to be among the most challenging social issues to our 21st century ageing society. Given their detrimental impact on physical and mental health (and vice versa), policy‐makers should invest in effective interventions to reduce loneliness or social isolation, or both. In January 2018, British Prime Minister Theresa May has set the example, by appointing Tracey Crouch as the country's first Minister for Loneliness. Reviews that study the effects of feasible and sustainable interventions, such as friendly visiting by a volunteer, on loneliness, social isolation and wellbeing, may provide useful information to the minister and other governments and organizations that are preparing to face the challenge.

## 3 OBJECTIVES

By systematically searching for individual studies, this review answered the following research question:

What is the effect of friendly visiting by a volunteer on feelings of loneliness, social isolation (primary outcomes) and wellbeing (i.e., life satisfaction, depressive symptom experiencing and mental health; secondary outcomes) in older adults?

## 4 METHODS

### 4.1 Criteria for considering studies for this review

#### 4.1.1 Types of studies

Since we applied quite specific criteria at the level of population and intervention, we included a broad range of study designs to ensure that the systematic review was as inclusive as possible.

Studies using an experimental design (randomized controlled trials, quasi‐ or non‐randomized controlled trials, controlled before and after studies or controlled interrupted time series) were included. In addition, as we anticipated that they would provide the majority of the available evidence, studies using an observational design (cohort studies, case‐control studies, controlled before and after studies, controlled interrupted time series, cross‐sectional studies) were eligible as well.

Other study designs such as case series, narrative reviews and non‐original studies such as editorials, book reviews, commentaries, and letters to the editor, were excluded. In addition, qualitative studies were not included in this review.

#### 4.1.2 Types of participants

Studies in community‐dwelling and institutionalized older adults (≥60 years of age) were included. Studies that also included younger adults (<60 years of age) were only included if: (1) they reported the results separately for ≥60‐year‐olds, or (2) they specifically defined the population as ‘older adults’ or ‘elderly’ and the average age of the participants was or exceeded the age of 60.

As this review was conducted to directly inform the friendly visiting programme of the Belgian Red Cross, which specifically aims at tackling loneliness within the general population of older adults, studies focusing exclusively on specific groups, such as widow(er)s or bereaved older adults, caregivers of older adults, hospitalized older adults, community‐dwelling older adults with severe mental or physical health problems (e.g., palliative care patients, clinically depressed older adults), were beyond the scope of this review.

#### 4.1.3 Types of interventions

Interventions for this systematic review included any frequency and any duration of friendly visiting by a volunteer (of any age) to an older adult (≥60 years of age). We define a volunteer as a person who does something, especially helping other people, willingly and without being forced or paid to do it; either in a formal or informal setting. The friendly visits should consist of friendly talking, playing games and/or reminiscing, with the sole purpose of reducing loneliness, social isolation, depressive symptoms, and/or improving life satisfaction and/or mental health in the older adult.

Interventions delivered by health or social care professionals were excluded from the review. As this review aimed at investigating the effect of face‐to‐face social interaction with others, again to directly inform the friendly visiting programme of the Belgian Red Cross, interventions delivered via computerized systems or telephone were excluded as well. In addition, studies concerning screening of older adults, small group meetings, support groups, social networks, extensive courses, computer courses at home and support for the bereaved were excluded.

Within experimental studies, the effect of friendly visiting was compared to no friendly visiting. For observational studies, the outcomes (see below) of older adults who received friendly visits would have been compared to those of older adults who did not receive friendly visits.

#### 4.1.4 Types of outcome measures

Studies were included if they quantitatively measured the effect of friendly visiting on at least one or more of the following primary or secondary outcomes.

##### Primary outcomes

The primary outcomes for this review were loneliness and social isolation.

Studies that measured loneliness were included, regardless of the measurement instrument used. Loneliness measuring instruments include, but are not limited to:

- Validated formal loneliness scales:
  ￮ UCLA 20‐Item Loneliness Scale (Russell, 1996);
  ￮ UCLA 3‐Item Loneliness Scale (Hughes, 2004);
  ￮ De Jong Gierveld 11‐Item Loneliness Scale (De Jong Gierveld, 1985; De Jong Gierveld, 1999);
  ￮ De Jong Gierveld 6‐Item Loneliness Scale (De Jong Gierveld, 2006);
  ￮ Social and Emotional 37‐Item Loneliness Scale for Adults (SELSA) (DiTommaso, 1993);
  ￮ Social and Emotional 15‐Item Loneliness Scale for Adults (SELSA‐S) (DiTommaso, 2004).
- Single‐item questions, such as:
  ￮ How often do you feel lonely? (hardly ever or never, some of the time, often);
  ￮ During the past week, have you felt lonely? (rarely or none of the time [e.g., less than 1 day], some or a little of the time [e.g., 1–2 days], occasionally or a moderate amount of time [e.g., 3–4 days], all of the time [e.g., 5–7 days]).

Studies that measured social isolation were included, as long as the measuring instrument used objectively quantified social isolation (i.e., by measuring the frequency of social contact and/or the size of the respondent's social network). Objective social isolation measuring instruments include, but are not limited to:

- Validated scales:
  ￮ Lubben Social Network 10‐Item Scale (Lubben, 1988);
  ￮ Lubben Social Network 6‐Item Scale (Lubben, 2006).
- Single‐item questions, such as:
  ￮ How often do you meet socially with friends, relatives or work colleagues?
  ￮ How often do you have contact with non‐cohabitant others?

Studies using instruments that measure social support in a subjective way (i.e., by measuring perceived social support), such as the Social Support Questionnaire and the Multidimensional Scale of Perceived Social Support, were excluded.

Studies that used a measure that combines objective quantification of social isolation with subjective measuring of perceived social support, such as the Duke Social Support Index 35‐Item Scale (George, 1989) and the Duke Social Support Index 10‐Item Scale (Wardian, 2013), were only included if the results of the objective subscales or scale domains were reported separately.

This systematic review was comprehensive regarding the timing of these measurements. In other words, we included:

- Studies that assessed an outcome once during the post‐intervention period (immediately after the intervention or in the longer term).
- Studies that assessed the same outcome multiple times during the post‐intervention period (e.g., immediately after the intervention and 6 months later),
- Studies that assessed the same outcome before the start of the intervention and post‐intervention.

Studies were not excluded solely on the basis of reporting of outcome data. To this end, we contacted the authors to ascertain whether the data for our outcomes of interest are unavailable due to lack of measurement or lack of reporting.

##### Secondary outcomes

Depressive symptom experiencing, life satisfaction and mental health outcomes were considered as secondary outcomes.

If a certain measurement instrument contained multiple items or subscales that covered outcomes that were not of interest, the study was excluded (see Differences between protocol and review). For this reason, the following scales were not deemed eligible for inclusion:

- Revised Social Dysfunction Rating Scale (RSDRS, Arthur, 1973): measures social interaction, which is not a direct sign of mental well‐being
- Blau's scale (Bogat, 1983): measures working, leisure, eating, sleeping, social contact, earning, parenting, loving, environment and self‐acceptance

### 4.2 Search methods for identification of studies

A comprehensive search for eligible published and unpublished studies and reports was performed to reduce the risk of publication bias and identify the best available evidence. No date, location or language restrictions were placed on the searches or included studies.

#### 4.2.1 Electronic searches

##### Electronic databases

Our search strategies are presented in Supporting Information: Appendix 1.

The following databases were searched from inception to May 11, 2020, with search for an update on August 11, 2021:

- The Cochrane Library (Cochrane Database of Systematic Reviews and Cochrane Central Register of Controlled Trials);
- MEDLINE (PubMed interface);
- Embase (Embase.com interface);
- PsycInfo and PsycArticles (psycnet.apa.org);
- ProQuest Sociology Database;
- Social Sciences Citation Index (Web of Science).

Search filters were not used, as they may prevent the retrieval of relevant papers. No language or publication data limits were applied.

##### Grey literature sources and handsearching

We consulted the following sources of grey literature, and searched the websites of organizations devoted to the specific topics of loneliness and ageing, to identify relevant unpublished studies and reports, between October 25 and November 29, 2021. Details on the searches can be found in Supporting Information: Appendix 1.

- Grey literature:
  ￮ Grey literature repositories:
    ￭ Grey Literature Report (www.greylit.org);
    ￭ OpenGrey (www.opengrey.eu);
    ￭ ClinicalTrials.gov (ClinicalTrials.gov);
    ￭ International Clinical Trials Registry Platform of the World Health Organisation (ICTRP, apps.who.int/trialsearch/Default.aspx).
  ￮ Other sources of grey literature:
    ￭ Google Scholar (scholar.google.be).
- Loneliness:
  ￮ Campaign to end loneliness in the UK (www.campaigntoendloneliness.org);
  ￮ Age UK (www.ageuk.org.uk/our-impact/policy-research/loneliness-research-and-resources);
  ￮ No Isolation in Norway (www.noisolation.com/global/research/);
  ￮ Together against loneliness by Coalitie Erbij in The Netherlands (In Dutch: Samen tegen eenzaamheid; www.samentegeneenzaamheid.nl);
  ￮ Friends for Good in Australia (www.friendsforgood.org.au).
- Ageing:
  ￮ Age UK (www.ageuk.org.uk/our-impact/policy-research/publications/);
  ￮ Centre for Ageing Better (www.ageing-better.org.uk/publications);
  ￮ International Longevity Centre UK (ILCUK, ilcuk.org.uk/reports/);
  ￮ WHO Ageing and life‐course Program (www.who.int/ageing/data-research/en/);
  ￮ National Ageing Research Institute (NARI) in Victoria, Australia (www.nari.net.au/publications/overview-about-publications).

#### 4.2.2 Searching other resources

##### Other reviews

The reference lists of the above identified systematic reviews on the effectiveness of interventions aiming to reduce loneliness or social isolation were scanned for relevant references.

##### Reference lists

The reference lists of included references were searched. In addition, the ‘Related Articles’ feature of the databases, if present, was used.

##### Contacting experts

This review was conducted in close collaboration with the Social Care Department of Belgian Red Cross. This Department runs a friendly visiting program, in which volunteers pay regular visits to older adults to tackle their feelings of loneliness and social isolation.

Furthermore, the review team also received content support from an external panel of social care experts (Vonk3 research centre of Thomas More University, Expertise centre Dementia Flanders, residential care centres, Public Centre for Social Welfare, Christian health insurance fund). These experts were contacted to help identify other relevant studies.

### 4.3 Data collection and analysis

#### 4.3.1 Selection of studies

All references were imported into the reference manager software EndNote X9 (EndNote, 2013) and duplicates were removed. Study selection was performed independently and in parallel by two evidence reviewers (JL and HS) in EndNote. In a first phase, titles and abstracts of the references identified by the search were screened. Full texts of potentially relevant papers were retrieved, and references that met the selection criteria were included for further analysis. Any relevant retraction statements and errata were examined. In addition, relevant conference abstracts identified through the above‐mentioned searches were included. Studies that met the selection criteria and had the outcomes of interest measured, but did not report these outcome data, were included and are described in the Results section. Any discrepancies between the two reviewers were resolved by consensus, and in case of disagreement a third reviewer was involved (EDB).

A PRISMA study selection flowchart is provided and a table of Characteristics of excluded studies with documented reasons for exclusion is presented.

As this review mainly aims to inform policy decisions, we decided to collect the best available evidence, rather than the highest tier of evidence. Due to the small number of available randomized controlled trials and the very low‐certainty evidence they provided, we therefore chose to include both randomized and non‐randomized controlled trials, in accordance with chapter 24 of the Cochrane Handbook for Systematic Reviews of Interventions (Reeves, 2022) and the recent GRADE guidance (Cuello‐Garcia, 2022).

#### 4.3.2 Data extraction and management

Data concerning the year in which the study was reported, the setting, the study design, and the basic characteristics of the study participants, interventions, and outcome measures were independently extracted by the two reviewers. To ensure consistency in the data collection process, a standardized and piloted data collection form was used (Supporting Information: Appendix 2).

By documenting all eligible available outcome measures in the Characteristics of included studies table, we were able to assess the potential for multiplicity of outcomes within the same study and handle them appropriately, following the guidance of the Cochrane Handbook (McKenzie, 2019).

If multiple methods were used to measure the same outcome within the same study, the reviewers selected the most relevant measure for analysis using the following decision rules:

- Outcomes measured via validated formal scales are more relevant than those measured using a single‐item question.
- Clinician‐rated outcome measures are more relevant than self‐reported measures.

If a single study had measured the same outcome at multiple time points, the reviewers extracted data from one short‐term time point (≤1 month after the intervention has ended), one intermediate‐term time point (>1 and ≤6 months after the intervention has ended) and one long‐term time point (>6 months after the intervention has ended).

During extraction, special attention was paid to ensure that multiple reports of the same study were not treated as multiple studies. For studies containing multiple intervention arms, the reviewers only extracted data on the intervention and control groups that were eligible to this review. For multi‐arm studies reporting on multiple relevant intervention arms, the findings from the different arms were reported and analysed separately. However, due to the low number of included studies and because we wanted to maximize the conclusions we could draw on the effectiveness of friendly visiting interventions, we decided to combine the data from the multiple relevant intervention arms when possible and subsequently compare those data to those of the control group.

For continuous outcomes that could be assumed normally distributed, we extracted means, standard deviations (or information to estimate standard deviations), and the number of participants in each group. We extracted post‐intervention values, unless the study authors only reported change‐from‐baseline scores or unless pre‐intervention values differed considerably between groups. In these cases, change‐from‐baseline scores were extracted or computed. To calculate the SDs for the change scores, we followed the guidance of the Cochrane Handbook (Higgins, 2019) and assumed a correlation coefficient of 0.8 for the outcome of life satisfaction, based on the paper by MacIntyre (1999).

Any discrepancies between the two reviewers were resolved through discussion or consulting other review co‐authors.

#### 4.3.3 Assessment of risk of bias in included studies

Individual studies were assessed for risk of bias, independently by the two reviewers (JL and HS). For randomized controlled trials, the Cochrane Risk of Bias tool was used to identify the methodological quality and potential shortcomings therein (Higgins, 2011). Study quality of non‐randomized experimental studies were assessed using the Risk of Bias In Non‐randomized Studies—of Interventions (ROBINS‐I) tool (Sterne, 2016).

#### 4.3.4 Measures of treatment effect

The two reviewers (JL and HS) independently calculated treatment effects in the Review Manager 5 software (Review Manager, 2014). Continuous outcomes were reported as mean differences (MD) with 95% confidence intervals (CIs). Dichotomous outcomes would have been reported as odds ratios (OR) or risk ratios (RR) with 95% CIs.

#### 4.3.5 Unit of analysis issues

In case of a multiarm study, we paid caution to ensure that the same group of participants was not included twice in a single meta‐analysis. In addition, paired data were analysed appropriately.

#### 4.3.6 Dealing with missing data

In case of missing data, we contacted the authors at least twice to obtain these data, if correspondence details were available.

Where possible, we calculated missing values (e.g., change scores, risk ratios, 95% CI and *p* values) from the available data, using the Review Manager 5 software (Higgins, 2019; Review Manager, 2014). If insufficient data were available to calculate missing values, we only analysed the available data and described the results from the studies with missing data narratively.

The issue of missing data and their potential impact on the findings is discussed in the Discussion.

#### 4.3.7 Assessment of heterogeneity

We had planned to assess heterogeneity by visually inspecting forest plots to investigate overlap in the confidence intervals for the results of the individual studies and by performing *χ* ^2^ tests. However, as no meta‐analyses were performed, this was not applicable.

#### 4.3.8 Assessment of reporting biases

We documented any evidence of potential selective or incomplete reporting in the Risk of bias assessment, and we discussed the extent to which this could potentially influence the findings. Since less than 10 studies were identified, publication bias could not be assessed through funnel plot analyses.

#### 4.3.9 Data synthesis

If two or more studies were identified that had investigated the effect of the same intervention on the same outcome, and data were sufficiently available, the data would have been pooled and random effects meta‐analyses would have been performed. As this was not possible because many studies did not report the necessary data, study findings were synthesized using vote counting based on the direction of effect (McKenzie, 2022). Synthesis was done separately for randomized and non‐randomized controlled trials, following the guidance of the Cochrane Handbook (Reeves, 2022). For each study, the effect of each intervention was categorized as beneficial or harmful based on the direction of effect. In this way, we were also able to avoid any issues concerning the use of different scales to measure the same outcome. Due to the limited number of studies, binomial testing to assess the significance of evidence for the existence of an effect in either direction was deemed inappropriate, due to the inherent uncertainty.

#### 4.3.10 Subgroup analysis and investigation of heterogeneity

Substantial statistical heterogeneity would have been explored by conducting subgroup analyses or meta‐regression to guard against potential issues of confounding. We hypothesized that heterogeneity may occur due to:

1. Housing situation: In contrast to nursing home residents, who experience a certain degree of social interaction with other residents and care personnel on a daily basis, community‐dwelling older adults may live their lives with minimal social interaction. Therefore, it was conceivable that the effect of friendly visiting would be larger in community‐dwelling older adults compared to institutionalized older adults.
2. Activities engaged in during friendly visits: We hypothesized that friendly visiting that includes the use of interactive materials (e.g., playing checkers, dominoes, jigsaw puzzles) would have a more profound beneficial effect on loneliness or social isolation, compared to friendly visiting where the volunteer only engages in conversation and other types of social interaction (e.g., taking a walk) with the older adult.
3. Frequency and duration of visits: Friendly visiting programmes that invest in high‐frequency visiting and/or longer visits by a volunteer may have a more substantial impact on loneliness or social isolation, compared to low‐frequency and/or short‐duration friendly visiting.
4. Diversity at the level of gender, race/ethnicity, culture and geopolitical region: Friendly visiting programmes aimed at alleviating loneliness and social isolation may affect older adults differentially across different gender, race/ethnicity, culture and geopolitical region.

However, because of the limited number of studies, heterogeneity could not be explored further (Deeks, 2019).

#### 4.3.11 Sensitivity analysis

Because of the limited included number of studies, sensitivity analyses with respect to the quality of studies to test the robustness of the meta‐analysis could not be conducted.

#### 4.3.12 Summary of findings and assessment of the certainty of the evidence

Two reviewers (JL and HS) independently assessed the overall certainty of the evidence using the GRADE approach, based on the limitations in study design (risk of bias assessment), imprecision, inconsistency, indirectness, and publication bias (Atkins, 2004; Schünemann, 2013). GRADE assessment was performed separately for randomized and non‐randomized controlled trials.

A Summary of findings table, containing a summary of the results of all the included studies and the overall confidence in the effect estimates for each outcome, was prepared using the GRADEpro software (www.gradepro.org).

## 5 RESULTS

### 5.1 Description of studies

#### 5.1.1 Results of the search

**Figure 1:**
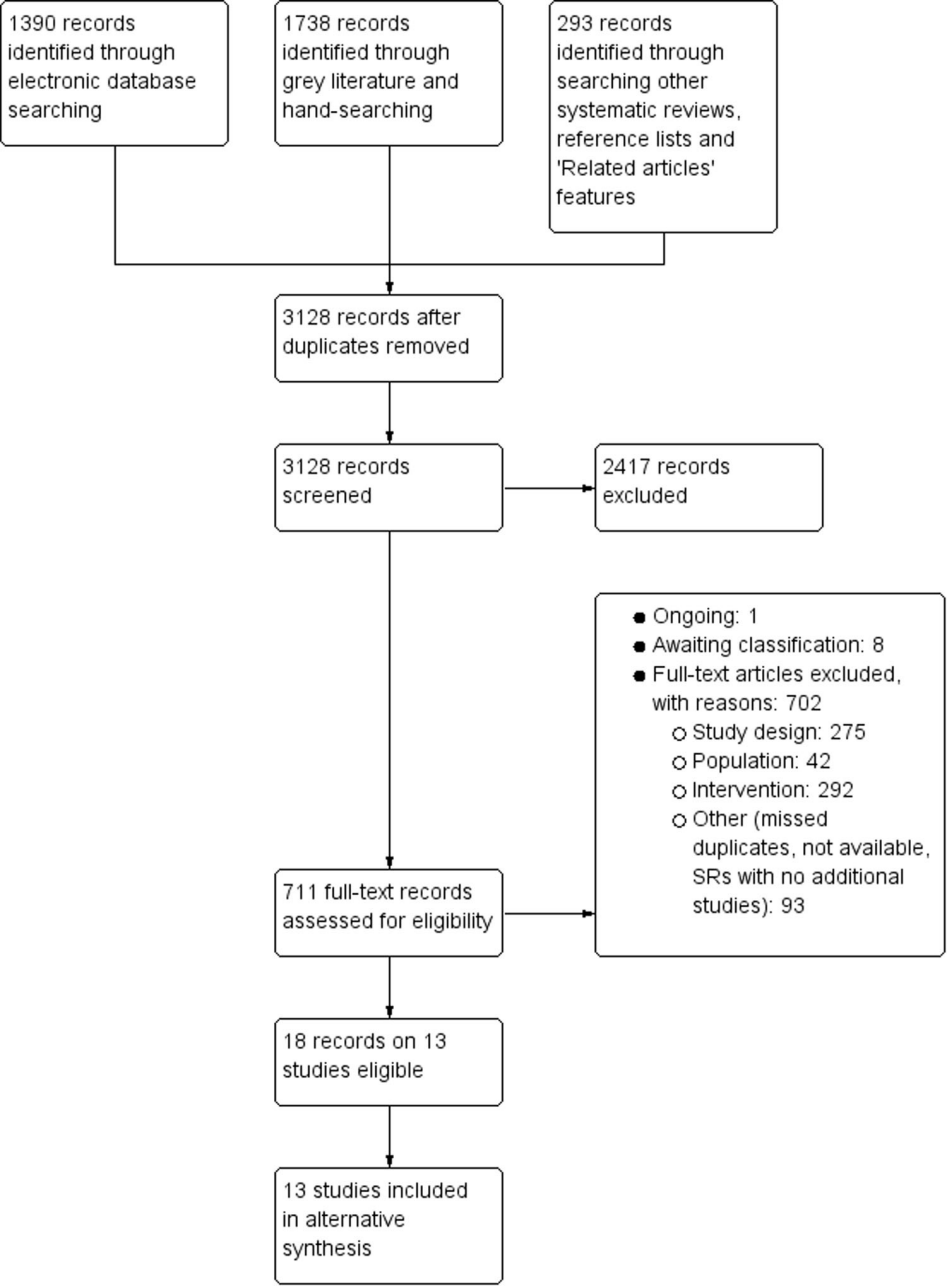
PRISMA study selection flowchart. SRs, stands for systematic reviews.

We identified 1390 references via database searching, 1738 additional records via grey literature and hand‐searching, and 293 records through searching other systematic reviews, reference lists and ‘Related articles’ features. After duplicate removal, titles and abstracts of the remaining 3128 records were screened. After full‐text screening and resolving disagreements, 18 records on 13 unique studies were included. In addition, we identified one ongoing study (Ninesling, 2018) and eight studies awaiting classification (Al‐Khazraji, 1974; Cattan, 2002; Cattan, 2003; ChiCTR1800017915; CTRI/2018/01/011466; NCT03405675; NCT03695133; NCT04224038). Figure 1 illustrates the PRISMA study selection flowchart, including reasons for article exclusion.

#### 5.1.2 Included studies

##### Study design

All 13 included studies were experimental in nature. Nine were randomized controlled trials (Calsyn, 1984; Haight, 1988–1992; Hautzinger, 1992; Keller, 1988; Lawlor, 2014; MacIntyre, 1999; McNeil, 1991–1995; Reinke, 1981; Schulz, 1976–1978), whereas the other four were non‐randomized controlled trials (Arthur, 1973; Bogat, 1983; Kahlbaugh, 2011; Mulligan, 1978).

##### Geographic and temporal setting

The vast majority (69%) of the studies were conducted in the United States (Arthur, 1973; Bogat, 1983; Calsyn, 1984; Haight, 1988–1992; Kahlbaugh, 2011; Keller, 1988; Mulligan, 1978; Reinke, 1981; Schulz, 1976–1978). Two studies took place in Canada (MacIntyre, 1999; McNeil, 1991–1995), one in Germany (Hautzinger 1992) and one in Ireland (Lawlor, 2014).

Overall, there were very little recent studies, with all but two (Kahlbaugh, 2011; Lawlor, 2014) being conducted during the previous century. Three studies were conducted in the 1970s (Arthur, 1973; Mulligan, 1978; Schulz, 1976–1978). Five studies (Bogat, 1983; Calsyn, 1984; Haight, 1988–1992; Keller, 1988; Reinke, 1981) and three studies (Hautzinger, 1992; MacIntyre, 1999; McNeil, 1991–1995) took place in the 1980s and 1990s, respectively.

##### Participants

Nine of the 13 studies included community‐dwelling older adults (Bogat, 1983; Calsyn, 1984; Haight, 1988–1992; Kahlbaugh, 2011; Keller, 1988; Lawlor, 2014; MacIntyre, 1999; McNeil, 1991–1995; Mulligan, 1978), whereas the other four (Arthur, 1973; Hautzinger, 1992; Reinke, 1981; Schulz 1976–1978) studied institutionalized (nursing home) residents. Mean or median ages of the studied participants ranged from 72 to 83 years old. The number of participants per study varied between 20 and 88, with an average number of 35 older adults. The vast majority (10/13) of studies included mainly female participants (between 69% and 91% females). In the remaining three studies, females represented 13% (McNeil, 1991–1995), 50% (Arthur, 1973) or an unknown percentage (Bogat, 1983) of all study participants.

In three studies, all participants were considered socially isolated at the start of the study, as assessed by referral agencies (Calsyn, 1984), home agencies (Keller, 1988) or professional nurses (MacIntyre, 1999). Arthur (1973) described the participants as all being ‘withdrawn, uncooperative, communicating very little, having few visitors’. Both of the studies that aimed to investigate the impact of friendly visiting on loneliness (Kahlbaugh, 2011; Lawlor,, 2014) measured the participants’ loneliness levels before the start of the study. In Lawlor (2014), all participants were lonely, that is, scored three or more on the De Jong Gierveld Loneliness Scale, or answered ‘Yes’ to the question ‘Would you say that much of the time during the past week you felt lonely?’. In Kahlbaugh (2011), mean baseline loneliness levels were 40 ± 9 and 37 ± 10 on the UCLA scale version 3 (score of 20 = not lonely, score of 80 = highest possible for loneliness) in the friendly visiting and control group, respectively.

Similarly, the three studies investigating the impact of friendly visiting on social isolation (Bogat, 1983; Lawlor, 2014; Mulligan, 1978) all measured social isolation levels at the start of the study. In Mulligan (1978), participants scored on average 1.4 and 2.2 on the Past Month Isolation index, where scores of 0 to 2 indicate social isolation. Lawlor (2014) did not report on the baseline social isolation levels, making it unclear how many of the study participants were socially isolated at the start of the study. Bogat (1983) used the number of daily telephone calls, and the number of visitors and visits made as measures for social isolation, which do not allow to determine the degree of social isolation in the study participants.

##### Interventions

Table 1 contains a complete description of each intervention for each individual study, according to the Template for Intervention Description and Replication (TIDieR) checklist (Hoffmann, 2014). Below, we narratively highlight some of the intervention components.

**Table 1:** Summary of intervention components.

Study	Brief name	Why	What**—**Materials	What**—**Procedures	Who provided	How	Where	When and how much	Tailoring	Modifications	How well—Planned	How well—Actually
Arthur 1973	1. Friendly visiting by the same volunteer 2. Friendly visiting by a different volunteer	Volunteer companions may improve the functioning level of the older adults in a nursing home in terms of general morale and personal adjustment. This might differ when visitors rotate.	Before the first visit, the investigator conducted a training session to orient the volunteers to nursing homes, the aged population and their needs, ethical considerations, and activities commonly performed by volunteers. No referral to actual materials used.	1. The older adult was visited by the same volunteer for 1.5 h per week for 10 weeks. No further information available. 2. The older adult was visited by a different volunteer for 1.5 h per week for 10 weeks. No further information available.	10 undergraduate students (median age 20 years, range 18‐29 years; 5 men and 5 women) from Auburn University, trained as mentioned in ‘What—Materials’	Face‐to‐face, individually	Nursing home where the older adults resided	1.5 h per week for 10 weeks	None reported	None reported	Not reported	No drop‐out occurred, nor did any of the friendly visitors depart. No further information on intervention adherence.
Bogat 1983	Friendly visiting	Creating an empathetic and caring atmosphere for the older adults may increase psychological wellbeing (but not as much as a network‐building visiting program; out of the scope of this systematic review)	The volunteers received 3 1.5‐h training sessions before any contact with the older adults. The training sessions (lectures, role plays, and discussions) focused on establishing skills central to helping relationship: understanding one's role in a relationship, learning basic helping skills, and trying out those skills. No referral to actual materials used.	A relationship‐oriented visiting program, in which visitors provided weekly 1‐h visits over a period of 3 months. No further information available.	13 advanced undergraduate university students in community psychology (age range 18‐31 years, 5 men and 8 women), trained as mentioned in ‘What ‐ Materials’. In addition, weekly 1‐h supervision sessions during the 3‐month intervention phase were used to generate resources, strategies, and support for the volunteers.	Face‐to‐face, individually	Not clearly reported, but presumably in the homes of the older adults	1 h per week for 3 months	None reported	None reported	Not reported	One of the older adults requested to discontinue the visits and therefore did not complete the study (reason is not reported). Another older adult died, and two refused to complete part of the questionnaire. No further information on intervention adherence.
Calsyn 1984	Friendly visiting	Companionship‐oriented friendly visiting may improve the life satisfaction of community‐dwelling older adults	Visitor training consisted of 3 4‐h sessions: 1. Biological, psychological and social aspects of aging + ground rules for visiting (keeping appointments, types of activities, potential legal issues such as liability) 2. Learning and practicing communication skills (active listening approach) 3. Learning and practicing communication skills—part 2 No referral to actual materials used.	Visitors engaged in a variety of activities with their clients including helping with activities of daily living such as shopping as well as some advocacy work with agencies such as the Social Security Administration and public aid. However, most of the visiting time was spent in companionship activities, primarily talking about common interests. Visitors turned in a journal sheet on each of their visits with information on activities that occurred during the visits.	21 volunteers (older volunteers referred by senior citizen organizations and undergraduate students enroled in a field placement course), trained as mentioned in ‘What—Materials’. They turned in a journal sheet on each of their visits with information on activities that occurred during the visits, information on any new problems and the volunteer's feelings about the visit. These journal sheets were turned in at bi‐weekly supervision sessions. These supervision sessions were small group sessions of 6 to 8 members led by one of the study authors. Each older adult was discussed at each meeting. Group members tried to give each other support and suggestions on more effective ways of communication, as well as suggestions regarding activities to do on the visit and/or agencies which might provide needed services. The last two sessions focused on termination issues.	Face‐to‐face, individually	Not clearly reported, but presumably in the homes of the older adults	Once a week for a period of 12 weeks. The length of the visit varied from older adult to older adult and from week to week, but generally lasted about 1.5 h per visit.	The researchers attempted to match the older adults and visitors on the basis of expressed interests.	None reported	Not reported	Four people did not complete the post‐test due to death or illness. No further information on intervention adherence.
Haight 1988–1992	Friendly visiting	The structured life review therapy (out of the scope of this systematic review) improves the overall well‐being of older adults, as compared to those older adults people who participate in a friendly visiting or testing process only	Several sessions were held to train the data collectors in Ivy's (1971) microcounseling skills before the visits began. These skills guide the interviewer to respond to the client in an open and accepting manner. No referral to actual materials used.	During the visit, they discussed the weather, health problems, current events and TV shows.	College students trained to be data collectors, as mentioned in ‘What ‐ Materials’. Testing was done by one data collector during the first and last visit, whereas friendly visits and life reviews were conducted by two other data collectors.	Face‐to‐face, individually	In the homes of the older adults	1 h for 6 consecutive weeks	None reported	None reported	Not reported	One each from the control and experimental groups said their children did not want strangers visiting them. No further information on intervention adherence.
Hautzinger 1992 (replication study of Schulz 1976–1978)	1. Friendly visiting controlled by the older adult in time, frequency and duration of visits 2. Friendly visiting where the older adult was informed about the time of the visits 3. Friendly visiting on the basis of a random schedule In this systematic review, data of all 3 friendly visiting interventions were combined and compared to the no visiting control group	Control and predictability of a positive social experience (i.e., the friendly visits) would result in significantly improved well‐being, health, increased activity and participation. Control with no explicit predictability would be significantly different from control with clear predictability. With regard to the comparison condition of random social contacts and the control group without corresponding contacts, no positive effects on health, well‐being and activity were expected.	Each visitor was assigned an older adult in each visiting condition, and was trained to carry out the experiment accurately. No referral to actual materials used.	The older adults were initially told by the visitors that they wanted to gain practical experience in dealing with older people and also knew that the older people would like to have someone with whom they could talk. Next, depending on the condition: 1. The visitor told the older adult that they would stay only as long as they wished (‘Then please tell me when to leave’). Shortly before saying goodbye, the visitor then asked if and if so when another visit would be possible (‘I enjoyed talking to you. When would you like me to come by again?’). 2. Towards the end, the visitor informed when she would be coming to visit next (‘I was very pleased chatting with you. I'll be back next… at… o'clock’). 3. No information was given, but on different days the visitor came by ‘accidentally’ and emphasized this accordingly (‘I thought I could come by today and visit you’). The farewell took place without making arrangements for further visits (‘I really enjoyed talking to you’). The visitor played a relatively passive role when interacting with the subject. All conversations were terminated with the visitor saying: ‘I really enjoyed talking to you’. No further information available.	14 female psychology students, trained as mentioned in ‘What ‐ Materials’. No further information available.	Face‐to‐face, individually	At the private, church‐affiliated and municipal retirement homes the older adults were living in	Once weekly for 40‐50 min during 9 weeks. Older adults were visited an average of 1.3 times per week.	None reported	None reported	Not reported	Not reported
Kahlbaugh 2011	1. Friendly visiting to play a Wii game 2. Friendly visiting to watch TV In this systematic review, data of both friendly visiting interventions were combined and compared to the no visiting control group	Older individuals who play activity simulation Wii games of their choice with a social partner for 1 h a week may report increased physical activity, less loneliness, and greater positive mood compared to those who either watch television with a social partner for 1 h a week but do not play Wii, or those who neither play Wii nor watch television with a partner.	1. Wii games 2. Television	Visitors engaged in either: 1. Playing a Wii game of the older adult's choice (all chose Wii bowling) 2. Watching television programs of the older adult's choice No further information available.	Undergraduate female research assistants were assigned to visit a participant either to play Wii or to watch television, and stayed with that participant over the course of the 10‐week period. The research assistants were encouraged to be socially responsive to their partners.	Face‐to‐face, individually	Not clearly reported, but presumably in the independent living residential apartments of the older adults	1 h per week for 10 weeks	The older adults in the Wii group could choose the game of their choice.	None reported	Not reported	Not reported
Keller 1988	Friendly visiting	Friendly visiting may improve the knowledge of the available health‐related community services (but this knowledge transfer is higher when the visiting visitors are trained on these services; out of the scope of this systematic review)	Visitors participated in an orientation in the form of an intake interview, where the director explained the community program and told the visitors what to expect in the visit situation, how to contact the homebound, procedures to follow in recording hours of volunteer service, and how to contact the office in case of emergency and questions. No referral to actual materials used.	A visiting program, in which visitors provided weekly visits over a period of 12 weeks. No further information available.	Senior citizens selected from a community's directory of persons registered in the Retired Senior Volunteer Program, who underwent the orientation as mentioned in ‘What ‐ Materials’	Face‐to‐face, individually	In the home of the homebound older adult	Once weekly for 12 weeks	None reported	None reported	Not reported	Not reported
Lawlor 2014	Friendly visiting	Home visiting schemes for older people experiencing loneliness in which the visitors are peers of the participants may improve loneliness	All volunteers attended 2 training sessions, which were conducted by the research team. Training content: Introduction to the project Role of the volunteer (including boundaries of their role) Background to loneliness and social isolation Local services for older people Trouble shooting Communication skills Role play Confidentiality No referral to actual materials used.	Initially the aim of these visits was to develop a rapport with the participant. The volunteer then encouraged the participant to identify a social connection they would like to make and that would be sustainable beyond the timeframe of the study. If a participant had difficulty identifying a connection the volunteer helped the participant in the process as they had knowledge of local services and social activities. Potential barriers were identified and feasible ways to overcome the barriers discussed with the participant.	Voluntary visitors were identified via local volunteer services and active retirement groups, based on the following criteria: aged over 55 years old, cognitively intact and with no significant memory problems had the capacity and commitment to undergo the training required had a full understanding of confidentiality agreed to undertake liaising with the research team if problems arose agreed to the Garda (Police) clearance process before taking up the role of volunteer Potential volunteers met with the research team and were asked to provide names of 2 referees before being selected and undergoing the training sessions mentioned in ‘What ‐ Materials’. Volunteers were supported through the following structures: Contact details and explicit support from the research team Feasible time commitment to the project Outline of responsibilities/volunteer policy Adequate training (see ‘What ‐ Materials’) Course handbook and information booklet on services and activities for older people in their locality Telephone call from a member of the research team following each visit Referral system so if a volunteer encountered a problem they will refer the problem to a member of the research team Discussion of problems by the team and decisions on how to best proceed with the referral Social event for all volunteers at the end of the study	Face‐to‐face, individually	In the home of the lonely older adult	Once weekly for 1 h for 10 weeks (over a ± 3‐month period)	None reported	None reported	Not reported	4 people in the intervention group dropped out. The study authors did not ask for any specific reason, so it is unclear if it was related to the intervention. No further information on intervention adherence.
MacIntyre 1999	Friendly visiting	Friendly visiting may improve life satisfaction and social support in the lonely and socially isolated older adults	The initial training programme was specifically designed to meet the needs of the individual volunteer and the older adult. Issues discussed included safety in relation to mobility aids, walkers, canes, wheelchairs and pertinent information related to the older adults’ medical diagnosis. Ongoing educational and training needs were met at the monthly volunteer meetings where topics identified by volunteer were discussed and, where necessary, presentations by professionals in the community added additional information and encouragement. No referral to actual materials used.	Activities during the visits included walks around the house, talking, assisting with care activities, reading, writing letters and often just listening. Clients indicated the visitors provided ‘company’ and gave them ‘something to do’.	Undergraduate students studying gerontology at the local University, who had provided references, and attended an interview process with the Coordinator of the Volunteer Programme so as to clarify their goals, assess their suitability and receive an orientation to theorganization's nurses’ service in the community. Next, they attended the initial training programme and the monthly volunteer meetings as described in ‘Materials—What’. The Coordinator of the VolunteerProgramme monitored the older adult/volunteer match by monthly written reports submitted by the volunteer, contact by telephone calls to older adult and volunteer, as well as their availability for visiting.	Face‐to‐face, individually	Not clearly reported, but presumably in the homes of the older adults	Once weekly for on average 3 h for 6 weeks	Volunteer‐older adult matches related to choice and interests of volunteer were a result of a specifically prepared interview process of each older adult and volunteer. The initial training programme of the volunteer was specifically designed to meet the needs of the older adult. No further information.	None reported	Not reported	3 participants were unable to complete the questionnaire themselves and were therefore excluded from the study. There is no reason to assume that this was caused by the intervention. No further information on intervention adherence.
McNeil 1991–1995	Home visiting with conversation	Accompanied exercise (out of the scope of this systematic review) and home visiting with conversation may reduce psychological depressive symptom experiencing in moderately depressed older adults, whereas accompanied exercise may also reduce somatic symptoms	None reported	The visit was performed by the same volunteer each time and consisted of casual conversation. It was made clear at the outset of the study that, although the student had taken several psychology courses, she was not a professional and could not help them to overcome personal problems in any specific therapeutic way. All older adults underwent a 4‐week termination period that involved the gradual reduction in the number of weekly contacts.	Undergraduate psychology students. No training or support reported.	Face‐to‐face, individually	In the home of the moderately depressed older adult	Two visits each week that gradually increased from 20 to 40 min in duration over a 6‐week period	None reported	None reported	Not reported	There were no drop‐outs, and cancelled visits (usually due to minor illness or social reasons) were rescheduled within several days. No further information on intervention adherence.
Mulligan 1978	Friendly visiting	Friendly visiting was expected to reduce social isolation, improve social adjustment and improve the mental state of the isolated older adults	An interview schedule, designed as a result of a pilot study, was part of each visit to assess the greeting behaviour, grooming, appearance of the living quarters, mental state and cognitive awareness of the older adult. To keep the format of the visit easy and casual, though structured, the visitors were trained to administer the interview schedule in a conversational tone while at the same time showing a friendly interest. No referral to actual materials used.	Friendly conversation, with the hope of reducing the social isolation of the elderly and improving	Five pairs of trained volunteer visitors (graduate students) from Teachers College, Columbia University, trained as mentioned in ‘What—Materials'	Face‐to‐face, individually	In the home of the isolated older adult	1 h‐long visits every 2 weeks for 6 months	None reported	None reported	Not reported	Not reported
Reinke 1981	1. Friendly visiting focusing on conversation 2. Friendly visiting with conversation and cognitive game playing	Both visiting conditions were expected to provide some cognitive and social stimulation (leading to improved morale) in socially isolated older adults, and the addition of games was expected to require more use of cognitive skills.	The visitors received 2 h of training concerning suggested ways of interacting with elderly persons and procedures for conducting each condition before beginning the visitation. No referral to actual materials used.	1. Visitors were instructed to engage residents in normal conversational interaction. Additional forms of social interaction (e.g., taking a walk, pasting photos in an album, making popcorn) were permissible if they did not resemble the playing of games. Weekly reports completed by the visitors indicated that the conversations were usually devoted to previous experiences of the subjects and the activities of the visitors. 2. Each subject was urged to play at least one game each visit in addition to the conversational component. Games judged to require the use of cognitive abilities and strategies were provided, beginning in the second week of visiting. Specifically, the games made available for subjects to choose from were checkers, dominoes, and Tri‐ominoes, concentration (a card game involving memory), ginrummy, crossword puzzles, jigsaw puzzles, and Mastermind. The visitors’ weekly reports indicated that nearly all subjects played a minimum of one game per visit and indeed all subjects played games several times during the program. Some subjects engaged in a greater variety of games than others. The dominoes and card games appeared particularly popular.	38 undergraduate students at the University of Kansas, trained as mentioned in ‘What—Materials’. They also received individual help and supervision as needed throughout the visitation period. Each visitor was assigned to visit two subjects, one in the conversation group and one in the conversation + cognitive game group.	Face‐to‐face, individually	In the nursing homes where the older adults resided	1 h per week for 8 weeks	The older adults in the conversation and cognitive game playing group could choose from a set of games.	None reported	Not reported	One of the older adults requested to discontinue the visits and therefore did not complete the study. The reason for the request is not reported. Due to interpersonal friction, two visitors were reassigned within the first 3 weeks. No further information on intervention adherence.
Schulz 1976–1978	1. Friendly visiting controlled by the older adult in time, frequency and duration of visits 2. Friendly visiting where the older adult was informed about the time of the visits 3. Friendly visiting on the basis of a random schedule	It was predicted that having control and predictability would result in significantly greater positive effects on the physical and psychological status indicators than random visits or no visits.	The visitors were ‘trained to carry out the experiment accurately’ (no further information). No referral to actual materials used.	Regardless of condition, all visitors made their initial contact with subjects by introducing themselves as Duke undergraduates and as friends of the experimenter. They stated that they were interested in having some first‐hand interaction experience with older individuals. They added that they were taking a course on aging at Duke and thought it would be a good experience ‘to get out into the real world and talk to some elderly people’. They also remarked that the experimenter had suggested that they ‘might enjoy having someone to talk to’. After delivering these opening statements, the visitors allowed the subject to control the content of the discussions that ensued. Next, depending on the condition: 1. The visitor told the adult that they would stay only as long as they wished (‘I don't want to take any more of your time today than you can afford to spare. So if you would like to stop at any time, please tell me’). Shortly before leaving, the visitor then asked if and if so when another visit would be possible (‘I enjoyed talking to you. Do you know when would be a good time for me to come back for another visit?’). 2. When arranging a visit, the visitor stated when she would be coming to visit next (‘I'll be at the home… I'll drop by to see you at… o'clock’). 3. No information was given, but on different days the visitor came by ‘accidentally’ and emphasized this accordingly (‘I decided to drop by and pay you a visit today’). The farewell took place without making arrangements for further visits (‘I really enjoyed talking to you’). The visitor played a relatively passive role when interacting with the subject, to keep their behaviour as constant as possible across all conditions. Each visitor was asked to keep a diary in which were recorded the number of visits made per week, the length of each visit, and, on a 9‐point Likert‐type scale, how much they enjoyed each visit.	5 undergraduate students (1 man and 4 women) at Duke University, each assigned an older adult in each visitation condition, and ‘trained to carry out the experiment accurately’ (no further information).	Face‐to‐face, individually	In the private, church‐affiliated retirement home where the older adults resided	An average of 1.3 times per week with the mean length of each visit being 50.8, 49.0, and 50.0 min for the random, predict, and informed groups, respectively	None reported	None reported	Not reported	Not reported

*Note*: Following the Template for Intervention Description and Replication (TIDieR) checklist (Hoffmann, 2014).

The duration of the friendly visiting programmes ranged from 6 to 12 weeks. In 10 of the 13 studies, the program consisted of weekly visits (Arthur, 1973; Bogat, 1983; Calsyn, 1984; Haight, 1988–1992; Hautzinger, 1992, Kahlbaugh, 2011; Keller, 1988; Lawlor, 2014; MacIntyre, 1999; Reinke, 1981). Two studies reported on more frequent visiting: twice per week (McNeil, 1991–1995) and 1.3 times per week (Schulz, 1976–1978). In Mulligan (1978), visits were 2‐weekly over a period of 6 months. The majority of studies employed a visit length of 1 h (Bogat, 1983; Haight, 1988–1992; Kahlbaugh, 2011; Lawlor, 2014; Mulligan, 1978; Reinke, 1981) or 1.5 h (Arthur, 1973; Calsyn, 1984). In McNeil (1991–1995), visits were gradually extended over time from 20 to 40 min. In the study by MacIntyre (1999), older adults were visited on average 3 h at a time. In Hautzinger (1992) and Schulz (1976–1978), visits lasted 40–50 min. These latter two studies aimed at determining the role of control and knowledge concerning the frequency, duration and time of the visits. To do so, the research used three different intervention groups: (1) one in which the older adults could control the time, frequency and duration of the visit; (2) one in which the older adults were informed about the time of the visits; and (3) one in which the older adults were visited on the basis of a random schedule, without being able to control when or how long a visitor came by, or without knowing when the next visit would take place.

In five studies, the friendly visiting interventions consisted of casual conversation (Calsyn, 1984; Haight, 1988–1992; McNeil, 1991–1995; Hautzinger, 1992; Schulz, 1976–1978), for instance talking about common interests or discussing the weather, health problems, current events and TV shows. In Lawlor (2014), visitors were asked to first develop a rapport with the older adult and next encourage them to identify a social connection they would like to make and that would be sustainable beyond the timeframe of the study. In MacIntyre (1999), activities included making walks around the house, talking, assisting with care activities, reading, writing letters and often just listening. The older adults indicated the visitors provided ‘company’ and gave them ‘something to do’.

In addition to a group of volunteers that engaged in casual conversation, Reinke (1981) also included a group of volunteers who both engaged in casual conversation and played at least one cognitive game (e.g., checkers, dominoes, gin rummy, Mastermind) with the older adults. Kahlbaugh (2011) had the volunteers either play Wii games or watching TV with the older adults. In four studies (Arthur, 1973; Bogat, 1983; Keller, 1988; Mulligan, 1978), no information was provided on the actual activities included in the friendly visiting intervention.

In nine studies (Arthur, 1973; Bogat, 1983; Haight, 1988–1992; Hautzinger, 1992; Kahlbaugh, 2011; MacIntyre, 1999; McNeil, 1991–1995; Reinke, 1981; Schulz, 1976–1978), the volunteers performing the friendly visiting were undergraduate students (e.g., in psychology, gerontology). In two studies, the visitors were older adults themselves (Keller, 1988; Lawlor, 2014), whereas one study used both older adults and students (Calsyn, 1984). Mulligan (1978) did not report on the characteristics of their voluntary friendly visitors. In most studies, the older adults were visited by the same volunteer (or a pair of volunteers in the case of Mulligan, 1978; and Reinke, 1981) each time. Arthur (1973) additionally included a group where the older adults were visited by a different volunteer every week.

##### Outcomes

The primary outcomes of loneliness and social isolation were studied in just two studies (Kahlbaugh, 2011; Lawlor, 2014) and three studies (Bogat, 1983; Lawlor, 2014; Mulligan, 1978), respectively. Life satisfaction was the most frequently studied outcome (in seven studies: Arthur, 1973; Bogat, 1983; Calsyn, 1984; Haight, 1988–1992; Kahlbaugh, 2011; MacIntyre, 1999; Reinke, 1981). Other relevant secondary outcomes included depressive symptom experiencing or depression (Haight, 1988–1992; Lawlor, 2014, McNeil, 1991–1995), wellbeing (Haight, 1988–1992, McNeil, 1991–1995), morale (Reinke, 1981), positive and negative mood (Kahlbaugh, 2011), mental health (Hautzinger, 1992) and functional mental disorders (McNeil, 1991–1995).

An overview of the scales used to assess these primary and secondary outcomes, including score ranges and cut‐off values for interpretation, is presented in Table 2.

**Table 2:** Scales used to measure outcomes.

Outcome	Scale	Score range and cut‐off values for interpretation	Study
Loneliness	De Jong Gierveld 11‐item Loneliness Scale	Scores range from 0 to 11: 0–2: not lonely 3–8: moderately lonely 9–10: severely lonely 11: very severely lonely	Lawlor 2014
	UCLA scale version 3	Scores range from 20 to 80: continuum from 20 (not lonely at all) to 80 (as lonely as possible)	Kahlbaugh 2011
Social isolation	10‐item Lubben Social Network Scale	Scores range from 0 to 50: ≤20: small social network 21 to 25: moderate small social network 26 to 30: moderate large social network ≥31: large social network	Lawlor 2014
	5‐item Past Month Isolation Index	Scores range from 0 to 10: 0–2: isolation	Mulligan 1978
Depressive symptom experiencing	8‐item Center for Epidemiologic Studies ‐ Depression Scale (CES‐D 8)	Scores range from 0 to 24: ≥9: clinically significant depressive symptoms	Lawlor 2014
	20‐item Zung's Self‐Rating Depression Scale (SDS)	Raw scores range from 0 to 80: <50: normal 50–59: minimal to mild depression 60–69: moderate to marked major depression >70: severe to extreme major depression Raw scores can be converted into percentiles; subjects with percentiles >50% are clinically depressed	Haight 1988–1992
	21‐item Beck Depression Inventory (BDI)	Scores range from 0 to 63: <10: none or minimal depression 10–18: mild to moderate depression 19–29: moderate to severe depression 30–63: extreme depression	McNeil 1991–1995
Life satisfaction	20‐item Life Satisfaction Index A (LSI‐A)	Scores range from 0 to 40: higher scores indicate a better life satisfaction	Arthur 1973; Bogat 1983; Haight 1988–1992; Kahlbaugh 2011; Reinke 1981
	13‐item Life Satisfaction Index Z (LSI‐Z)	Scores range from 0 to 26: higher scores indicate a better life satisfaction	Calsyn 1984; MacIntyre 1999
Psychological wellbeing	10‐item Affect‐Balance Scale (ABS)	Scores range from 0 to 20: higher scores indicate higher psychological wellbeing	Haight 1988–1992
	24‐item Memorial University of Newfoundland Scale of Happiness (MUNSH)	Scores range from 0 to 48: higher scores indicate higher psychological wellbeing	McNeil 1991–1995
Presence of functional mental disorders	Mental Status Schedule (MSS)	Scores higher than the mean (3.1 at the first visit, and 1.7 at the final visit) indicate the presence of functional mental disorders	Mulligan 1978
Morale	22‐item Philadelphia Geriatric Center Morale Scale (PGC)	Scores range from 0 to 17: <9: low morale 10–12: mid‐range morale 13–17: high morale	Reinke 1981
Positive and negative mood	20‐item Positive and Negative Affect Scale (PANAS)	Scores range from 10 to 50 for both the 10‐item Positive and the 10‐item Negative Affect Scale: higher scores indicate a more positive/negative mood	Kahlbaugh 2011
Mental health (as assessed by a nurse)	NAR subtest of the Nuremberg Age Inventory	No information available	Hautzinger 1992

Two studies did not provide any extractable data on any of the primary/secondary outcomes of interest (Keller, 1988; Schulz, 1976–1978). In the randomized controlled trial by Keller (1988), the study authors investigated the impact of a friendly visiting program on the older adults’ knowledge of eight community services (e.g., visiting nurses, home delivered meals, homemaker health aides). In their randomized controlled trial, Schulz 1976–1978 studied the impact of friendly visiting on loneliness, activity (e.g., number of visits, number of phone calls made), zest for life, level of hope, happiness and usefulness levels. As the authors did not report or analyse the data of the four groups separately (only analyses reported compared the no treatment + random groups to the predict + control groups), we were not able to extract data on these primary and secondary outcomes.

See Characteristics of included studies for additional details on the study characteristics can be found on page 40.

#### 5.1.3 Excluded studies

We identified 49 studies that at first sight appeared to meet the inclusion criteria (and a reader might plausibly expect to see among the included studies), but were excluded nonetheless on further inspection. The large majority of these studies (36/49) were excluded on the basis of intervention. The most frequent reason for exclusion was that the volunteers engaged in much more than friendly talking, playing games and/or reminiscing, and the goal of their visits greatly exceeded the purpose of reducing loneliness, social isolation, depressive symptoms, and/or improving life satisfaction and/or mental health. For example, volunteers provided domiciliary care services, such as assistance with eating, shopping, exercising, taking medication, liaising with local health workers, etc. to improve (physical) health. Detailed reasons for exclusion of each of the 49 studies are provided in the Characteristics of excluded studies can be found on page 50.

### 5.2 Risk of bias in included studies

**Figure 2:**
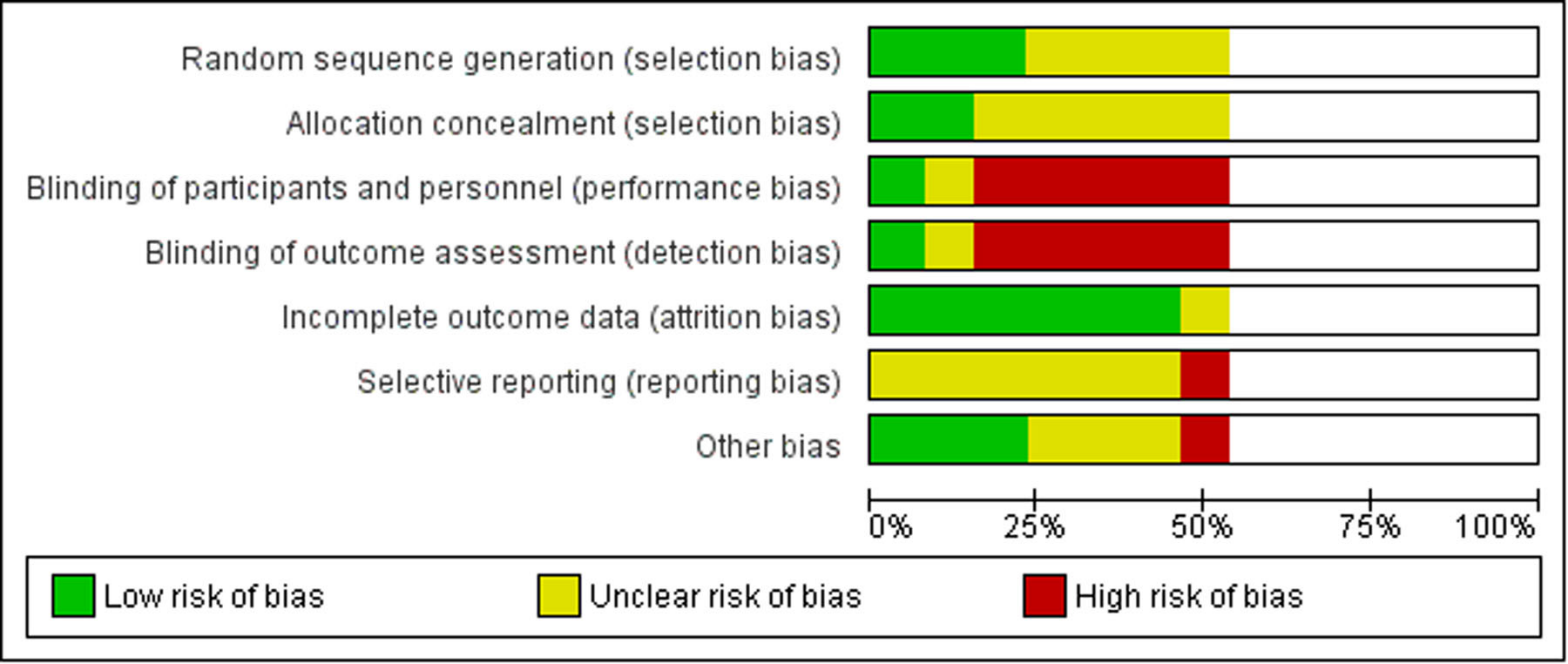
Risk of bias graph for the seven randomized controlled trials that provided extractable data on any of the primary/secondary outcomes.

**Figure 3:**
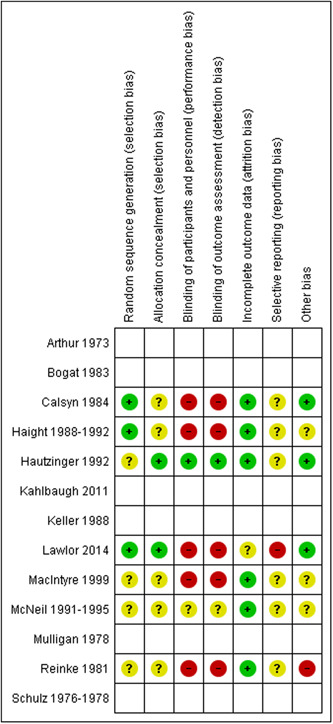
Risk of bias summary for the seven randomized controlled trials that provided extractable data on any of the primary/secondary outcomes.

For the randomized controlled trials, risk of bias was assessed using the Cochrane Risk of Bias tool (Higgins, 2011). The results are presented in Figures 2 and 3 and are summarized narratively in the sections below. Detailed judgements by domain can be found in the Characteristics of included studies. For the two studies that did not provide any extractable data on any of the primary/secondary outcomes (Keller, 1988; Schulz, 1976–1978), no risk of bias assessment was performed.

**Figure 4:**
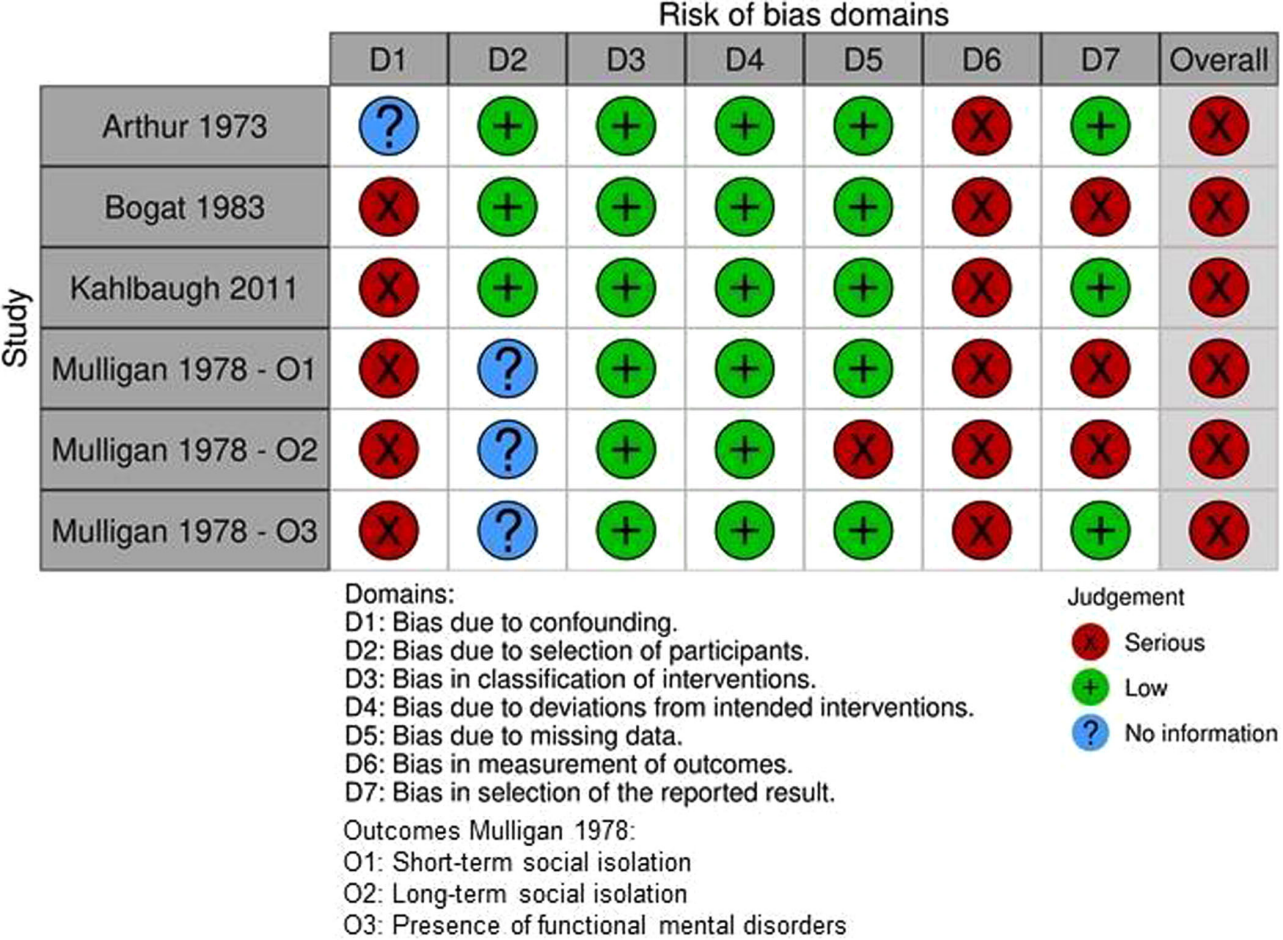
Traffic light plot ROBINS‐I assessment.

For the four non‐randomized controlled trials (Arthur, 1973; Bogat, 1983; Kahlbaugh, 2011; Mulligan, 1978), the Risk of Bias tool assessment was not applicable and boxes were left blank in Figure 3. For these trials, a ROBINS‐I assessment was done instead. The results are presented in a traffic light plot in Figure 4. Detailed judgements, together with the overall risk of bias judgements, can be found in Supporting Information: Appendix 3. In the paragraphs below, we provide a narrative synthesis for the seven domains. For the study of Mulligan (1978), judgements were different across the different outcomes, which explains why there are multiple lines for this study in the traffic light plot in Figure 4.

Three of the four studies (Bogat, 1983; Kahlbaugh, 2011; Mulligan, 1978) were found to be at serious risk of bias due to confounding due to the overall or partial lack of randomization. The fourth study (Arthur, 1973) did not provide sufficient information to make a proper judgement.

Bias due to selection of participants, bias in classification of interventions, bias due to deviations from intended interventions and bias due to missing data were all judged to be low for three of the four trials (Arthur, 1973; Bogat, 1983; Kahlbaugh, 2011).

Mulligan (1978) was at low risk of bias in classification of interventions and bias due to deviations from intended interventions, but at serious risk of bias due to missing data for the outcome of long‐term social isolation. This serious risk of bias resulted from substantial drop‐out between the last visit to the 1‐year follow‐up time point, which was also imbalanced between the intervention and control group. The study provided insufficient information to make a proper judgement on bias due to selection of participants.

All four studies were at serious risk of bias in measurement of outcomes. As friendly visiting was part of the intervention, it was impossible to blind the older adults, who were also often the outcome assessors themselves. Because most of the data were self‐reported and subjective, this lack of blinding may have affected the results due to social desirability bias. In addition, in the study by Bogat (1983), outcomes were collected in different manners in the intervention and control group.

Risk of bias in the selection of the reported result was serious for Bogat (1983), and for the outcomes of short‐term and long‐term social isolation in the study by Mulligan (1978). In Bogat (1983), some of the data were reported as post‐test means adjusted for pre‐test scores, whereas others were shown as mean changes between post‐ and pre‐test scores. In the Methods section, Mulligan (1978) mentioned the use of two scales for the measurement of social isolation, that is, the Adulthood Isolation Index and the Past Month Isolation Index. However, only one of these measures was reported in the Results section. The risk of bias in the selection of the reported result was low in Arthur (1973), Kahlbaugh (2011) and for the outcome of presence of functional mental disorders in the study by Mulligan (1978).

As all four studies were judged to be at serious risk of bias in at least one domain, but not at critical risk of bias in any domain, a judgement of serious risk of bias was assigned to these non‐randomized controlled trials.

#### 5.2.1 Allocation (selection bias)

In the majority (4/7) of the randomized controlled trials, randomization sequence generation was inadequately reported (Hautzinger, 1992; MacIntyre, 1999; McNeil, 1991–1995; Reinke, 1981). Five studies failed to report adequately on allocation concealment (Calsyn, 1984; Haight, 1988–1992; MacIntyre, 1999; McNeil, 1991–1995; Reinke, 1981). In just one RCT (Lawlor, 2014), both aspects were performed and reported adequately.

#### 5.2.2 Blinding (performance bias and detection bias)

As friendly visiting was part of the intervention, it was impossible to blind the older adults, who were also often the outcome assessors themselves. This lack of blinding may have affected the results due to social desirability bias. Likewise, blinding of the visitors was not possible either. In studies where the visiting volunteers themselves gathered the data, it is conceivable that they have (subconsciously) influenced the responses of the older adults.

As a result, all randomized controlled trials were judged to be at high risk of performance and detection bias, except for Hautzinger (1992) and McNeil (1991–1995). The latter provided insufficient information to make an appropriate judgement. Hautzinger (1992) was at low risk of performance and detection bias. In this study, the lack of blinding will not have influenced the data on clinician‐rated mental health, as these were rated by a researcher who was not involved in the study and was blinded to the allocation of the participants.

#### 5.2.3 Incomplete outcome data (attrition bias)

All but one of the randomized controlled trials were at low risk of attrition bias. Lawlor (2014) provided insufficient information to make an appropriate judgement on the completeness of the outcome data.

#### 5.2.4 Selective reporting (reporting bias)

One randomized controlled trial was at high risk of reporting bias. Lawlor (2014) did not report the results of multiple outcomes, although they were listed in the Methods section. The authors explained that ‘This was a short report for the funders reporting the significant findings’ and that they did no longer have access to the results (email conversation with Gillian Paul). All other studies were assessed as at unclear risk of reporting bias, as study protocols were not available and it was not convincingly clear that all the expected outcomes were included.

#### 5.2.5 Other potential sources of bias

We assessed one randomized controlled trial as being at high risk of bias for reasons other than those mentioned above. In Reinke (1981), all 49 residents expressed an interest in being visited, which may have biased the results. Second, it was unclear which types of social interaction (e.g., taking a walk, pasting photos in an album, making popcorn) were used by the ‘conversation’ visitors. Therefore, it is unclear if the effects should be attributed to the conversation or to another type of social interaction. Third, the results of the analyses in this article were questioned by one of the authors, who reanalysed the data in a second article (Denney, 1988). The other authors, Reinke and Holmes, replied to the comments of Denney in a third article (Reinke, 1988), pointing out errors made in the reanalyses and possible reasons for the differences in findings. Therefore, the results of this article are of questionable quality.

### 5.3 Effects of interventions

See also: Summary of findings Table 1 for the short‐term impact of friendly visiting by a volunteer; Table 3 for the extracted data; and Table 4 for the synthesis by vote counting based on direction of effect.

**Table 3:** Extracted data.

Outcome	Comparison	Effect size	#studies, # participants	Reference
**PRIMARY OUTCOMES**				
***Short‐term loneliness***				
*RCTs*				
Loneliness (one‐month total score)	Friendly visiting vs. no friendly visiting	5.3 vs. 6.7 MD: −1.4 MD adjusted for baseline levels: −1.1, 95% CI [−2.10; −0.10] (*p* = 0.027)	1, 40 vs. 48§	Lawlor, 2014
*Non‐RCTs*				
Loneliness (change score between first and final visit)	Friendly visiting (playing Wii or watching TV) vs. no friendly visiting	0.16 ± 6.69* vs. 3.0 ± 9.1** MD: −2.84, 95% CI [−10.0;4.3] (*p* = 0.36)***	1, 28 vs. 7§	Kahlbaugh, 2011
***Intermediate‐term loneliness***				
*RCTs*				
Loneliness (3‐month total score)	Friendly visiting vs. no friendly visiting	5.3 vs. 7.0 MD: −1.7 MD adjusted for baseline levels: −1.4, 95% CI [−2.3; −0.5] (*p* = 0.003)	1, 39 vs. 47§	Lawlor, 2014
***Short‐term social isolation***				
*RCTs*				
Social isolation (one‐month total score)	Friendly visiting vs. no friendly visiting	23.3 vs. 21.5 MD: 1.8 MD adjusted for baseline levels: 2.2, 95% CI [−0.05; 4.5] (*p* = 0.055)	1, 40 vs. 48§	Lawlor, 2014
*Non‐RCTs*				
Social isolation (mean change score between first and final visit)	Friendly visiting vs. no friendly visiting	0.1 vs. 0.3 MD: −0.2£ ^,^ † (*p* > 0.05)	1, 11 vs. 11§	Mulligan, 1978
Number of daily telephone calls (mean change between post‐test and pre‐test)		18.95 vs. 7.19 MD: 11.76£ ^,^ † *F*(2, 30) = 1.13 (*p* = 0.34)****	1, 12 vs. 12§	Bogat, 1983
Number of visitors and visits made (mean change between post‐test and pre‐test)		13.90 vs. 5.04 MD: 8.86£ ^,^ † *F*(2, 30) = 0.18 (*p* = 0.84)****		
Current networks (post‐test mean adjusted for pre‐test score)		9.54 vs. 11.41 MD: −1.87£ ^,^ † F(2, 31) = 0.65 (*p* = 0.53)****		
***Intermediate‐term social isolation***				
*RCTs*				
Social isolation (3‐month total score)	Friendly visiting vs. no friendly visiting	23.8 vs. 22.2 MD: 1.6 MD adjusted for baseline levels: 2.1, 95% CI [−0.1; 4.2] (*p* = 0.065)	1, 39 vs. 47§	Lawlor, 2014
*Non‐RCTs*				
Social isolation (mean change score between final and 6‐month follow‐up visit)	Friendly visiting vs. no friendly visiting	2.1 vs. 0.1 MD: 2£ (*p* < 0.05)	1, 8 vs. 5§	Mulligan, 1978
**SECONDARY OUTCOMES**				
***Short‐term depressive symptom experiencing***				
*RCTs*				
Depression at 8‐week post‐test (with pre‐test as covariate)	Friendly visiting vs. no friendly visiting	££ ^,^ † F(2,48): 1.22 (*p* = 0.30)	1, 16 vs. 19§	Haight, 1988
Depressive symptom experiencing (1‐month total score)		2.8 vs. 3.6 MD: −0.8 MD adjusted for baseline levels: −0.51, 95% CI [−1.47; 0.5] (*p* = 0.314)	1, 40 vs. 48§	Lawlor, 2014
Depressive symptoms (total BDI score at post‐test)		11.8 ± 4.0 vs. 14.7 ± 3.7 MD: −2.9£££ (*p* < 0.05)	1, 10 vs. 10§	McNeil, 1991
***Intermediate‐term depressive symptom experiencing***				
*RCTs*				
Depressive symptom experiencing (3‐month total score)	Friendly visiting vs. no friendly visiting	2.7 vs. 3.8 MD: −1.1 MD adjusted for baseline levels: −0.6, 95% CI [−1.4; 0.2] (*p* = 0.229)	1, 39 vs. 47§	Lawlor, 2014
***Long‐term depressive symptom experiencing***				
*RCTs*				
Depression at 1‐year post‐test (mean ± SD)	Friendly visiting vs. no friendly visiting	22.5 ± 16.68 vs. 16.6 ± 8.64 MD: 5.9, 95% CI [−4.4; 16.2]¥ (*p* = 0.26)***	1, 13 vs. 12§	Haight, 1992
***Short‐term life satisfaction***				
*RCTs*				
Life satisfaction (post‐test data, ±2 weeks after final visit)	Friendly visiting vs. no friendly visiting	12.62 ± 5.03 vs. 13.85 ± 3.08 MD: −1.23, 95% CI [−3.96; 1.50] (*p* = 0.43)***	1, 21 vs. 13§	Calsyn, 1984
Life satisfaction (change score between 8‐week post‐test and pre‐test)		−1.50 vs. 1.26 MD: −3.76£ ^,^ † F(2,33): 0.10 (*p* = 0.91)	1, 16 vs. 19§	Haight, 1988
Life satisfaction (change score between post‐test and pre‐test)		2.83 ± 1.7 vs. −3.3 ± 5.6 MD: 6.13, 95% CI [2.53; 9.73] (*p* = 0.0017)***	1, 12 vs. 10§	MacIntyre, 1999
Life satisfaction	Friendly visiting with focus on conversation vs. no friendly visiting	11.32 vs. 9.09 MD: 2.23£ (*p* < 0.05)	1, 12 vs. 12§	Reinke, 1981
	Friendly visiting with conversation and cognitive game playing vs. no friendly visiting	11.19 vs. 9.09 MD: 2.10£ ^,^ † (*p* > 0.05)	1, 15 vs. 12§	
*Non‐RCTs*				
Life satisfaction (change score)	Friendly visiting by the same volunteer each week vs. no friendly visiting	(*p* < 0.06)££ ^,^ †	1, 10 vs. 10§	Arthur, 1973
	Friendly visiting by a different volunteer each week vs no friendly visiting	(*p* < 0.005)££	1, 10 vs. 10§	
Life satisfaction (post‐test mean adjusted for pre‐test score)	Friendly visiting vs. no friendly visiting	8.95 vs. 9.49 MD: −0.54£ ^,^ † *F*(2, 31) = 1.21 (*p* = 0.31)****	1, 12 vs. 12§	Bogat, 1983
Life satisfaction (post‐test data)	Friendly visiting (playing Wii or watching TV) vs. no friendly visiting	11.83 ± 4.72* vs. 12.43 ± 3.3 MD: −0.6, 95% CI [−3.60; 2.40] (*p* = 0.75)***	1, 28 vs. 7§	Kahlbaugh, 2011
***Long‐term life satisfaction***				
*RCTs*				
Life satisfaction at 1−year post‐test (mean ± SD)	Friendly visiting vs. no friendly visiting	21.5 ± 7.36 vs. 21.7 ± 7.61 MD: −0.2, 95% CI [−6.1; 5.7] (*p* = 0.95)***	1, 13 vs. 12§	Haight, 1992
***Short‐term mental health (*** = ***psychological wellbeing, mental health, presence of functional mental disorders, morale, positive mood, negative mood)***				
*RCTs*				
Psychological well‐being (MUNSH at post‐test, controlled for pre‐test differences)	Friendly visiting vs. no friendly visiting	29.8 ± 8.6 vs. 27.7 ± 8.9 MD: 2.1££££ (*p* < 0.05)	1, 10 vs. 10§	McNeil, 1995
Psychological well‐being (change score between 8‐week post‐test and pre‐test)		−0.94 vs. 1.11 MD: −2.04£ ^,^ † *F*(2,33): 0.30 (*p* = 0.74)	1, 16 vs. 19§	Haight, 1988
Mental health as assessed by a nurse (change score)		1.59 ± 6.71* vs. −4.3 ± 6.8** MD: 5.89, 95% CI [1.16; 10.62] (*p* = 0.0188)***	1, 28 vs. 11§	Hautzinger, 1992
Morale (post‐test means)	Friendly visiting with focus on conversation vs. no friendly visiting	27.46 vs. 29.21 MD: −1.75£ ^,^ † (*p* > 0.05)	1, 12 vs. 12§	Reinke, 1981
	Friendly visiting with conversation and cognitive game playing vs. no friendly visiting	28.27 vs. 29.21 MD: −0.94£ ^,^ † (*p* > 0.05)	1, 15 vs. 12§	
*Non‐RCTs*				
Functional mental disorders (change in mean MSS count between first and final visit)	Friendly visiting vs. no friendly visiting	−1.4 vs. 0.2 MD: −1.6£££££	1, 11 vs. 11§	Mulligan, 1978
Positive mood (post‐test data)	Friendly visiting (playing Wii or watching TV) vs. no friendly visiting	32.16 ± 7.20* vs. 27.14 ± 8.90 MD: 5.02, 95% CI [−2.09; 12.13] (*p* = 0.12)***	1, 28 vs. 7§	Kahlbaugh, 2011
Negative mood (Post‐test data)		12.60 ± 4.41* vs. 14 ± 4.65 MD: −1.4, 95% CI [−5.21; 2.41] (*p* = 0.46)***		
***Long‐term mental health (*** = ***psychological wellbeing, mental health, presence of functional mental disorders, morale, positive mood, negative mood)***				
*RCTs*				
Psychological well‐being at 1‐year post‐test (mean ± SD)	Friendly visiting vs. no friendly visiting	9.9 ± 5.95 vs. 9.3 ± 7.37 MD: 0.6, 95% CI [−4.7; 5.9]¥ (*p* = 0.82)***	1, 13 vs. 12§	Haight, 1992

*Note*: Mean ± SD (unless otherwise indicated).

Abbreviations: CI, confidence interval; MD, mean difference; OR, odds ratio; RR, risk ratio; SD, standard deviation.

* Data for two or three groups combined by the reviewers using formulae from the Cochrane Handbook for Systematic Reviews on Interventions (Table 6.5.a).

** SD for change score calculated according to the Cochrane Handbook for Systematic Reviews on Interventions (https://training.cochrane.org/handbook/current/chapter-06#section-6-5-2-8), assuming a correlation coefficient of 0.8 for the outcome of life satisfaction (based on MacIntyre, 1991).

*** Calculations (MD, 95% CI and *p* value) done by the reviewers using Review Manager software.

**** Calculations of *p* value of *F* test done by the reviewers using online tool (https://www.socscistatistics.com/pvalues/fdistribution.aspx).

^£^ No SDs available, CI cannot be calculated.

^££^ No raw data available, effect size and CI cannot be calculated.

^£££^ A *t*‐test of post‐test group means would not indicate a significant difference between intervention and control (*p* = 0.11). Pre‐test scores were probably taken into account for statistical testing of effect, hence the *p* < 0.05 mentioned in the paper.

^££££^ SDs of post‐test group means cannot be used to calculate CI of effect, because pre‐test scores were taken into account for statistical testing of effect.

^£££££^ No formal test was performed by the authors. Data are lacking for any calculations by the reviewers.

^¥^ Imprecision (large variability of results).

^†^ Imprecision (lack of data).

^§^ Imprecision (limited sample size).

**Table 4:** Synthesis by vote counting based on direction of effect.

Outcome	Number of studies and study design	Number of effects in favour of friendly visiting	Number of effects in favour of control	Number of unknown effects	Probability (%)
Social isolation (short‐term)	2 non‐RCTs	3	1	0	75
Depressive symptom experiencing (short‐term)	3 RCTs	2	0	1	100
Life satisfaction (short‐term)	4 RCTs	3	2	0	60
	3 non‐RCTs	1	2	0	33
Mental health (short‐term)	4 RCTs	2	3	0	40
	2 non‐RCTs	2	0	1	100

Abbreviation: RCT, randomized controlled trial.

#### 5.3.1 Primary outcomes

##### Loneliness

###### Short‐term (≤1 month after the end of the intervention)

Two studies with 123 older adults reported on the impact of friendly visiting on short‐term loneliness (Kahlbaugh, 2011; Lawlor, 2014). A third study, the randomized controlled trial by Schulz (1976–1978), did not provide usable data on the effect of friendly visiting on loneliness. It is unclear how these results would have impacted the results and conclusions.

In the randomized controlled trial by Lawlor (2014), loneliness at the 1‐month timepoint was on average 1.1 lower (95% CI 2.10 to 0.10 lower, *p* = 0.027) in the friendly visiting group compared to the control group. Mean loneliness scores at this time were 5.3 in the friendly visiting group and 6.7 in the control group, indicating that the older adults in both groups remained ‘moderately lonely’ (see Table 2). Evidence was of very low certainty.

In the non‐randomized controlled trial by Kahlbaugh (2011), the mean increase in loneliness between the first and final visit was 2.84 lower (95% CI 10 lower to 4.3 higher, *p* = 0.36; Analysis 1.1) in the friendly visiting (with either Wii‐playing or TV‐watching) group compared to the control group. Evidence was of very low certainty.

###### Intermediate‐term (>1 and ≤6 months after the end of the intervention)

In the randomized controlled trial by Lawlor (2014), loneliness at the 3‐month timepoint was on average 1.4 lower (95% CI 2.3 to 0.5 lower, *p* = 0.003) in the friendly visiting group compared to the control group. Mean loneliness scores remained 5.3 in the friendly visiting group (same as at the 1‐month timepoint) and was 7 in the control group, indicating that the older adults in both groups remained ‘moderately lonely’. Evidence was of very low certainty.

###### Long‐term (>6 months after the end of the intervention)

None of the studies reported on long‐term loneliness.

##### Social isolation

###### Short‐term (≤1 month after the end of the intervention)

Short‐term social isolation was an outcome of interest for the studies of Bogat (1983), Lawlor (2014) and Mulligan (1978), including 134 older adults in total. A fourth study, the randomized controlled trial by Schulz (1976–1978), did not provide usable data on the effect of friendly visiting on social isolation. It is unclear how these results would have impacted the results and conclusions.

In the randomized controlled trial by Lawlor (2014), mean social isolation levels at the 1‐month timepoint adjusted for baseline scores were 2.2 higher (95% CI 0.05 lower to 4.5 higher, *p* = 0.055) in the friendly visiting group compared to the control group. Mean scores on the Lubben Social Network Scale at this timepoint were 23.3 in the friendly visiting and 21.5 in the control group, indicating moderate small social network sizes in both groups. Evidence was of very low certainty.

The non‐randomized controlled trial of Mulligan (1978), including 22 older adults in total, showed a lower mean change in social isolation between the first and final visit (MD: –0.2, no 95% CI reported, *p* > 0.05) in the friendly visiting group. Mean scores on the Past Month Isolation Index increased from 1.4 at the first visit to 1.5 at the final visit, indicating that the older adults remained socially isolated.

In a second non‐randomized controlled trial by Bogat (1983), the mean change in the number of daily telephone calls, and the number of visitors and the number of visits made, was higher in the 12 older adults that received friendly visits compared to the 12 older adults in the control group. In contrast, the mean number of current networks used by the older adults after the intervention had ended was 1.87 lower (no 95% CI reported, *F*‐test: 0.65, *p* = 0.53) in the friendly visiting group than in the control group.

Synthesizing the results of these two non‐randomized controlled trials (Bogat, 1983; Mulligan, 1978) by vote counting based on the direction of effect (see Table 4 for tabulated form), three of the four effects (75%) favoured the friendly visiting intervention. Evidence was of very low certainty.

###### Intermediate‐term (>1 and ≤6 months after the end of the intervention)

In the randomized controlled trial by Lawlor (2014), social isolation levels at the 3‐month timepoint adjusted for baseline levels were on average 2.1 higher (95% CI 0.1 lower to 4.2 higher, *p* = 0.065) in the friendly visiting group compared to the control group. Mean scores on the Lubben Social Network Scale at this timepoint were 23.8 in the friendly visiting and 22.2 in the control group, indicating moderate small social network sizes in both groups. Evidence was of very low certainty.

In the non‐randomized controlled trial by Mulligan (1978), the improvement in social isolation between the final and the 6‐month follow‐up visit was on average 2 larger (no 95% CI reported, *p* < 0.05) in the friendly visiting group, compared to the control group. Mean scores on the Past Month Isolation Index increased from 1.5 at the final visit to 3.6 at the 6‐month follow‐up visit in the friendly visiting group, whereas the mean score of the control group went from 2.3 to 2.4. Evidence was of very low certainty.

###### Long‐term (>6 months after the end of the intervention)

None of the studies reported on long‐term social isolation.

#### 5.3.2 Secondary outcomes

##### Depressive symptom experiencing

###### Short‐term (≤1 month after the end of the intervention)

Three randomized controlled trials including 143 older adults investigated the effect of friendly visiting on short‐term depression or depressive symptom experiencing (Haight, 1988–1992; Lawlor, 2014; McNeil, 1991–1995). As Haight 1988‐1992 did not report raw data and Lawlor (2014) did not report standard deviations, we were unable to conduct a meta‐analysis.

In Haight (1988–1992), no differences in depression were found between the friendly visiting group and the control group immediately after the intervention (*F*‐test: 1.22, *p* = 0.30). In the study by Lawlor (2014), depressive symptom experiencing at the 1‐month timepoint adjusted for baseline scores was on average 0.51 lower (95% CI 1.47 lower to 0.5 higher, *p* = 0.314) in the friendly visiting group than in the control group. Mean scores at this timepoint on the CES‐D 8 scale were 2.8 and 3.6 in the friendly visiting and control group, respectively, indicating no clinically significant depressive symptoms in either of the groups. In McNeil (1991–1995), friendly visiting resulted in lower depressive symptom scores, compared to no friendly visiting (on average 2.9 lower, no 95% CI reported, *p* < 0.05). Mean scores on the Beck Depression Inventory were 11.8 and 14.7 in the friendly visiting and control group, respectively, indicative of mild to moderate depression.

Combining the results of these three randomized controlled trials (Haight, 1988–1992; Lawlor, 2014; McNeil, 1991–1995), two of the two effects (100%) favoured the friendly visiting intervention. Evidence was of very low certainty.

###### Intermediate‐term (>1 and ≤6 months after the end of the intervention)

In the randomized controlled trial of Lawlor (2014), depressive symptom experiencing at the 3‐month timepoint adjusted for baseline scores was on average 0.6 lower (95% CI 1.4 lower to 0.2 higher, *p* = 0.229) in the friendly visiting group than in the control group. Mean scores at this timepoint on the CES‐D 8 scale were 2.7 and 3.8 in the friendly visiting and control group, respectively, again indicating no clinically significant depressive symptoms in either of the groups. Evidence was of very low certainty.

###### Long‐term (>6 months after the end of the intervention)

In the randomized controlled trial of Haight (1988–1992), depression levels after 1 year were on average 5.9 higher (95% CI 4.4 lower to 16.2 higher, *p* = 0.26; Analysis 1.2) in the friendly visiting group than in the control group. Mean scores on the Self‐Rating Depression Scale were 22.5 and 16.6 in the friendly visiting and control group, respectively, which are both considered ‘normal’ (i.e., not depressed). Evidence was of very low certainty.

##### Life satisfaction

###### Short‐term (≤1 month after the end of the intervention)

Short‐term life satisfaction was assessed by seven studies with 219 older adults in total (Arthur, 1973; Bogat, 1983; Calsyn, 1984; Haight, 1988–1992; Kahlbaugh, 2011; MacIntyre, 1999; Reinke, 1981).

Of the four randomized controlled trials (Calsyn, 1984; Haight, 1988–1992; MacIntyre, 1999; Reinke, 1981), only two (Calsyn, 1984; MacIntyre, 1999) provided sufficient data to perform meta‐analysis. Nevertheless, this meta‐analysis resulted in an effect estimate with considerable heterogeneity (see Analysis 1.3; *p* = 0.001, *I* ^2^ = 90%). Therefore, we decided not to report the pooled value (as we judged it to be misleading) and instead used synthesis by vote counting based on the direction of effect. Combining the results of these four randomized controlled trials, three of the five effects (60%) favoured friendly visiting. Evidence was of very low certainty.

Due to limited reporting of the raw data, we were not able to conduct meta‐analysis on the results of the three non‐randomized controlled trials (Arthur, 1973; Bogat, 1983; Kahlbaugh, 2011). In Arthur (1973), the group of participants that were visited by a different volunteer each week displayed a higher increase in life satisfaction compared to the control group (no raw data reported, *p* < 0.005). This could not be shown for the participants that were visited by the same volunteer each week (no raw data reported, *p* < 0.06). In the study by Bogat (1983), life satisfaction after the intervention was on average 0.54 lower in the friendly visiting group than in the control group (mean score 8.95 vs. 9.49 out of a possible 40, with higher scores indicating better life satisfaction; *F*‐test: 1.21, *p* = 0.31). In Kahlbaugh (2011), life satisfaction levels were on average 0.6 lower (95% CI 3.6 lower to 2.4 higher, *p* = 0.75; Analysis 1.4) in the friendly visiting group than in the control group (mean score 11.83 vs. 12.43 out of a possible 40).

Taken together, one of the three effects (33%) favoured the friendly visiting intervention, with a 95% confidence interval of 1 to 91, and a *p* value of 1. Again, these results show that there is insufficient evidence to say that friendly visiting has an effect on short‐term life satisfaction compared to control. Evidence was of very low certainty.

###### Intermediate‐term (>1 and ≤6 months after the end of the intervention)

None of the studies reported on intermediate‐term life satisfaction.

###### Long‐term (>6 months after the end of the intervention)

In Haight (1988–1992), life satisfaction levels after 1 year were on average 0.2 lower (95% CI 6.1 lower to 5.7 higher, *p* = 0.95; Analysis 1.5) in the friendly visiting group than in the control group (mean score 21.5 vs. 21.7 out of a possible 40). Evidence was of very low certainty.

##### Mental health

###### Short‐term (≤1 month after the end of the intervention)

Psychological wellbeing was an outcome of interest for two randomized controlled trials with 55 older adults (Haight, 1988–1992; McNeil, 1991–1995). As Haight (1988–1992) did not report standard deviations, we were unable to pool these data. Mental health as rated by a nurse was measured and reported by the randomized controlled trial of Hautzinger (1992). The presence of functional mental disorders and morale were reported by the non‐randomized controlled trial of Mulligan (1978) and the randomized controlled trial by Reinke (1981), respectively. Finally, the non‐randomized controlled trial of Kahlbaugh (2011) looked at the effect of friendly visiting on positive and negative mood. Since it was not possible to perform meta‐analyses, we decided to use synthesis by vote counting based on the direction of effect for all of these short‐term mental health‐related outcomes combined.

Unfortunately, the randomized controlled trial by Schulz (1976–1978), did not provide usable data on the effect of friendly visiting on zest for life, level of hope, happiness and usefulness. It is unclear how these results would have impacted the results and conclusions.

In McNeil (1991–1995), friendly visiting resulted in increased psychological wellbeing after the intervention compared to no friendly visiting (mean scores 29.8 vs. 27.7 out of a possible 48, MD: 2.1, no 95% CI reported, *p* < 0.05). In Hautzinger (1992), who included 39 older adults, friendly visiting resulted in an improvement in mental health as rated by a nurse compared to no friendly visiting (MD: 5.89, 95% CI [1.16; 10.62], *p* = 0.0188; Analysis 1.6). In contrast, in Haight (1988–1992), friendly visiting caused a mean decrease of 2.04 in psychological wellbeing at 8 weeks compared to before the intervention (mean change score on the Affect Balance Scale of –0.94 vs. 1.11, *F*‐test: 0.30, *p* = 0.74). Similarly, in Reinke (1981), when compared to the control group, the morale of the participants after the intervention was on average 1.75 lower (no 95%CI reported, *p* > 0.05) in the participants that received visits with focus on conversation and on average 0.94 lower (no 95% CI reported, *p* > 0.05) in those that received visits with focus on conversation and cognitive game playing.

Combining the results of the four randomized controlled trials (Haight. 1988–1992; Hautzinger, 1992; McNeil, 1991–1995; Reinke, 1981), two of the five effects (40%) favoured friendly visiting. Evidence was of very low certainty.

As the non‐randomized controlled trial by Mulligan (1978) did not report standard deviations of the change in functional mental disorders in the 22 included older adults, we could not judge the potential impact of friendly visiting on this outcome. In Kahlbaugh (2011), positive mood levels and negative mood levels were on average 5.02 higher (mean scores 32.16 vs. 27.14 out of a possible 50, 95%CI 2.09 lower to 12.13 higher, *p* = 0.12; Analysis 1.7) and 1.4 lower (mean scores 12.6 vs. 14 out of a possible 50, 95% CI 5.21 lower to 2.41 higher, *p* = 0.46; Analysis 1.8), respectively, in the friendly visiting participants compared to the control participants. Combining the results of these two non‐randomized controlled trials, two of the two effects (100%) favoured friendly visiting. Evidence was of very low certainty.

###### Intermediate‐term (>1 and ≤6 months after the end of the intervention)

None of the studies reported on intermediate‐term mental health.

###### Long‐term (>6 months after the end of the intervention)

In Haight (1988–1992), psychological wellbeing levels after 1 year were on average 0.6 higher (mean scores of 9.9 vs. 9.3 out of a possible 20, 95% CI 4.7 lower to 5.9 higher, *p* = 0.82; Analysis 1.9) in the friendly visiting group than in the control group. Evidence was of very low certainty.

Unfortunately, the randomized controlled trial by Schulz (1976–1978), did not provide usable data on the effect of friendly visiting on the older adults’ zest for life at 24‐, 30‐ and 42‐months follow‐up. It is unclear how these results would have impacted the results and conclusions.

## 6 DISCUSSION

### 6.1 Summary of main results

To the best of our knowledge, this is the first systematic review that has collected and synthesized the available data on the effectiveness of volunteers providing friendly face‐to‐face visits to alleviate loneliness or social isolation, or both, in older adults. We have identified nine randomized and four non‐randomized controlled trials that were relevant to elucidate this research question.

At the moment, the evidence is very uncertain about the effect of friendly face‐to‐face visiting by a volunteer on improving loneliness, social isolation and wellbeing in older adults. Overall, the identified evidence is scarce and of very low certainty, which precludes any conclusions about the added value of friendly face‐to‐face visiting by a volunteer.

### 6.2 Overall completeness and applicability of evidence

In total, we identified 13 studies (nine randomized and four non‐randomized controlled trials) that investigated the impact of friendly face‐to‐face visiting by a volunteer on our outcomes of interest in older adults. For each of these outcomes, with the exception of life satisfaction (seven studies), the number of studies was limited to one to three. The vast majority of the studies were conducted in the United States (10 studies), included community‐dwelling older adults (nine studies) and concerned intergenerational visiting by volunteering undergraduate students (nine studies). Due to the scarcity of evidence for each outcome, we were not able to perform subgroup analyses with regard to geopolicital region, culture, race/ethnicity or housing situation (i.e., community‐dwelling vs. institutionalized). Also, we were not able to investigate the possible influences of diversity across gender, the frequency and duration of the visits, or the activities engaged in during the friendly visits (i.e., using interactive materials vs. social interaction only). Regarding the latter, subgroup analyses would have been very difficult nonetheless, due to the lack of clear descriptions of the interventions in the currently available studies. In the Implications for research, we discuss what type of future research is needed, and how it can best be designed and executed, to increase the overall completeness and applicability of the evidence.

### 6.3 Quality of the evidence

The vast majority (5/7) of the randomized controlled trials with usable data were at high risk of performance and detection bias. In addition, six of the seven trials were at unclear risk of selection bias. Therefore, the certainty of the evidence provided by the randomized controlled trials was downgraded by two levels due to risk of bias. Subsequently, the certainty of the evidence was downgraded by one levels for imprecision because of the limited sample sizes, wide confidence intervals and/or lack of data. We did not downgrade further for indirectness, inconsistency or publication bias, resulting in very low‐certainty evidence.

With regard to the four non‐randomized controlled trials, for each of the outcomes, the certainty of evidence was downgraded by two levels due to serious risk of bias, and by an additional level because of imprecision. We did not downgrade for indirectness, inconsistency or publication bias, resulting in very low‐certainty evidence.

Although we acknowledge that non‐randomized controlled trials are at larger risk of selection bias (see also Implications for research), we feel that in this case, the problem of inadequate blinding overshadows the lack of randomization. Therefore, we judged that the randomized and the non‐randomized controlled trials provide evidence of equal certainty.

### 6.4 Potential biases in the review process

By pre‐defining and documenting the review objectives and study eligibility criteria a priori in the published review protocol (Laermans, 2020), we minimized the potential for bias in the review process. In addition, both review authors (JL, HS) were methodologists, and not content experts, which further decreases the risk of bias. However, making decisions to in‐ or exclude studies on the basis of the aim of the intervention (‘with the sole purpose of reducing loneliness, social isolation, depressive symptoms, and/or improving life satisfaction and/or mental health’) required some judgement on their behalf, as many of the studies did not report this explicitly. It is therefore conceivable that some studies who did not (clearly) report on their specific aim were inappropriately in‐ or excluded. Nevertheless, we feel that by contacting the study authors in case of missing information, we have done everything possible to avoid this. Moreover, when in serious doubt and attempts to obtain additional information from the authors were unsuccessful, we classified the studies as ongoing.

Although we used a comprehensive search strategy, we might have missed studies because we included outcome‐specific search terms (e.g., loneliness, social isolation) in our search strings. Also, whilst we also searched for grey literature, we may have missed unpublished program reports that were not made publicly available by government agencies or non‐profit organizations.

Other potential bias in our review might have arisen from the poor reporting in many of the included studies. Often, data were missing, and a fair number of our attempts to contact the authors were unsuccessful because contact details were not available, authors did not respond or data were no longer available. This may have affected the completeness of our data as well as our risk of bias assessment, which may for some studies be harsher than necessary. In addition, the incomplete reporting only allowed us to synthesize the data through vote counting based on the direction of effect. Although this synthesis method is considered acceptable and may be considered superior to a narrative, it is less powerful than methods that combine p‐values of studies. The method does not provide information on the magnitude of the effects, and does not account for differences in the relative sizes of the different studies (McKenzie, 2022).

### 6.5 Agreements and disagreements with other studies or reviews

As mentioned in Why it is important to do this review, several existing systematic reviews have looked at the effectiveness of a wide range of interventions aiming to reduce loneliness or social isolation.

In the systematic review of Cattan (2005), ‘one‐to‐one’ interventions included home visits by professionals providing health assessments or services, telephone support‐therapy by social services, friendly telephone calls by peers and social support visits by volunteers. For the majority of these interventions, the reviewers were unable to demonstrate a significant effect in reducing social isolation and loneliness. Although the review only included one study on friendly visiting by a volunteer (Mulligan, 1978), we have reached the same conclusion that the effectiveness of home visiting and befriending schemes remains unclear.

In the McDaid (2015) review, five studies provided moderate evidence that friendship programmes can enhance various aspects of older peoples’ mental wellbeing and address issues of loneliness and isolation. Just one of these five studies fit our eligibility criteria and was included in our review (Lawlor, 2014). The other four were excluded from this review on the basis of study design (Pope, 2013) or intervention (Butler, 2006; Martina, 2006; Stevens, 2006 see Characteristics of excluded studies for the first two). Therefore, it is hard to compare the review results against ours. It shows however, that depending on the choices made on how to cluster interventions, systematic reviewers may reach different conclusions entirely.

Siette (2017) investigated the effectiveness of a wide range of befriending interventions (social support delivery through face‐to‐face encounters at home, in support groups, or via telephone contact) in a very diverse population of interest (adults of any age with any type of physical or mental condition). The review authors were unable to provide firm conclusions on the effect of befriending on loneliness, depression and quality of life. They concluded that there was moderate‐certainty evidence from seven of the 14 studies that showed small improvements in combined patient‐reported primary outcomes. Since just two of the 14 included studies fit our eligibility criteria (MacIntyre, 1999; McNeil, 1991–1995), we are not able to point out agreements and disagreements with this review.

In their integrative review, Gardiner (2018) included a wide range of interventions to reduce social isolation and loneliness among older people. The majority of interventions reported some success in reducing social isolation and loneliness, but the quality of evidence was generally weak. None of the four included befriending studies fitted our eligibility criteria.

None of these systematic reviews allowed to make clear statements on the effectiveness of friendly face‐to‐face visiting by a volunteer to the generalizable older population, that is, older adults that do not suffer from any serious physical or mental illness. Therefore, with our systematic review, we aimed for a systematic collection, extraction and analysis of studies looking specifically at the effectiveness of friendly visiting by a volunteer is effective in reducing loneliness or social isolation, or both, in older, otherwise healthy, adults. Unfortunately, the currently available data did not allow us to make clear statements either. At the root of the problem lie several issues that were already highlighted in an overview of 14 systematic reviews by Victor (2018), that is, the limited number of studies, the low sample sizes and the lack of clear (numerical) reporting, and that are confirmed by our systematic review. In the Implications for research, we discuss how these issues may be tackled in the future.

It will be interesting to see if the ongoing systematic review of Landeiro (2017), on the effectiveness of health promotion interventions on social isolation or loneliness in older people, reaches similar conclusions. In addition, the ongoing systematic review and network meta‐analysis by Lee (2021), that aims to determine the comparative efficacy of interventions to alleviate social isolation and loneliness of community‐dwelling older adults by comparing direct and indirect interventions, may shed further light on the effectiveness of friendly visiting interventions, and the individual components of these complex interventions, by a volunteer.

## 7 AUTHORS’ CONCLUSIONS

### 7.1 Implications for practice

Due to the very low‐certainty evidence, we are unsure about the effectiveness of face‐to‐face friendly visiting by a volunteer with regard to improving loneliness, social isolation, depressive symptom experiencing, life satisfaction and mental health outcomes in older adults. Decision‐makers that consider implementing face‐to‐face friendly visiting as a way to alleviate loneliness or social isolation, or both, in older adults should take into account this uncertainty.

### 7.2 Implications for research

First of all, this review highlights the need for additional randomized controlled trials. Interestingly, a previous meta‐analysis investigated whether the success of certain loneliness reduction interventions could be attributed to study design, rather than to the quality of the intervention (Masi, 2011). This revealed that uncontrolled before‐after studies and non‐randomized controlled trials studies yielded larger mean effect sizes as compared to randomized controlled trials. However, this finding might originate from a combination of regression towards the mean and selection bias, which uncontrolled before‐after studies and non‐randomized controlled trials are prone to. Therefore, randomized controlled trials, that minimize selection bias and the effect of regression towards the mean, remain the preferred study design.

These randomized controlled trials should include more participants and should be better designed and executed. Special attention should be given to minimizing the risk of social desirability bias, which arises from the fact that blinding the older adults from the visiting intervention is impossible, and that loneliness and life satisfaction are very subjective outcomes. One option is to use third‐party, non‐involved interviewers. With regard to more objectifiable outcomes, such as social isolation and mental health, researchers should focus on using scales that can be administered by (non‐involved) clinicians or professionals to measure these outcomes. Additionally, future research should include the cost‐effectiveness of these interventions, and also consider studying any potentially harmful effects of withdrawal from the intervention.

In addition, to increase the applicability of the evidence, more studies should be conducted outside of the United States. It would be interesting to see if studies can detect differences between the effectiveness of the interventions between community‐dwelling and institutionalized older adult, as well as across culture, race/ethnicity and gender. Also, additional studies may wish to use different frequencies and durations of the visits, or have volunteers engage in different types of activities during the friendly visits.

Finally, future studies should provide clear information about every aspect of the friendly visiting interventions according to the items in the TIDieR checklist, including the specific aims and planned and actual intervention adherence, to allow reliable implementation and replication of research findings. Also, they should make sure to report all raw data transparently.

On another note, during our review process, we encountered several studies that investigated the effectiveness of providing social support in non‐face‐to‐face ways (e.g., through frequent telephone or video call contact) during the global COVID‐19 pandemic. Future systematic review teams may wish to consider collecting evidence on the effectiveness of this type of contact to stay socially connected. This evidence may provide useful information to policy‐makers, for example, in dealing with future pandemic outbreaks that require social distancing.

## CONTRIBUTIONS OF AUTHORS

Jorien Laermans (JL): developed search strings, performed study selection, extracted and interpreted data, drafted the manuscript, critically revised and approved the final manuscript. Hans Scheers (HS): developed search strings, performed study selection, extracted and interpreted data, critically revised and approved the final manuscript. Philippe Vandekerckhove (PV): assisted with data interpretation, critically revised and approved the final manuscript. Emmy De Buck (EDB): designed the research question, assisted with data interpretation, critically revised and approved the final manuscript.

## DECLARATIONS OF INTEREST

JL, HS, PV and EDB are employees of the Belgian Red Cross and have no further interests to declare. One of the activities of the Belgian Red Cross is to run a friendly visiting program, in which volunteers pay regular visits to older adults to tackle their feelings of loneliness and social isolation.

## DIFFERENCES BETWEEN PROTOCOL AND REVIEW

In our original protocol (Laermans, 2020), we stated that interventions delivered via computerized systems or telephone would be excluded. However, in performing the review, we decided that combined interventions were eligible for inclusion as well, as long as the studies included a control group that allowed to analyse the impact of face‐to‐face friendly visits. For example, a study comparing telephone support + face‐to‐face visits to telephone support only would be included, as it would allow to determine the effect of the face‐to‐face visit component of the intervention.

With regard to the secondary outcomes, we decided to only include studies that used instruments that allowed for the analysis of depressive symptom experiencing, life satisfaction, and/or mental health outcomes. If a certain measurement instrument contained multiple items or subscales that covered outcomes that were not of interest, the study was excluded.

For this reason, the following scales were not deemed eligible for inclusion:

- Revised Social Dysfunction Rating Scale (RSDRS, Arthur, 1973): measures social interaction, which is not a direct sign of mental well‐being
- Blau's scale (Bogat, 1983): measures working, leisure, eating, sleeping, social contact, earning, parenting, loving, environment and self‐acceptance

Methods not implemented:

- As we did not include studies that only reported a composite measure of two or more of the outcomes of interest, extraction and analysis of the composite measure was not performed. Similarly, we did not include studies that contained data on overall scale findings, but also on the different dimensions addressed by the scale. Therefore, extraction of just the overall scale results was not necessary.
- Although we had planned to analyse the data from experimental studies (i.e., randomized controlled trials, quasi‐ or non‐randomized controlled trials, controlled before and after studies or controlled interrupted time series) and observational studies (i.e., cohort studies, case‐control studies, controlled before and after studies, controlled interrupted time series, cross‐sectional studies) separately, we only identified experimental studies. Therefore, there was no need to perform separate analyses.
- As none of the studies contained skewed continuous data, there was no need to extract medians, ranges, and *p* values of non‐parametric tests.
- As we did not encounter controlled before and after studies, it was not necessary to extract mean or median change‐from‐baseline scores, or to compute them ourselves.
- No dichotomous outcomes were reported in the included studies in this review. Hence, there was no need to extract the number of events and the number of participants in each (intervention or control) group. Extraction of odds ratios or risk ratios (both crude and adjusted ratios, if available), along with their 95% confidence intervals (CIs) and *p* values, was not applicable either. In addition, there was no need to collect missing data from the study authors with regard to combining dichotomous and continuous data for the same outcome or predictor.
- No cluster‐randomized trials were identified. Therefore, recalculation of effects, taking into account the clustering effect, was not necessary.
- For the outcome of short‐time life satisfaction, we initially performed a meta‐analysis of two studies (Analysis 1.3). Because of substantial heterogeneity (*I*² = 90%) and inconsistency in the direction of effect, we decided not to do a meta‐analysis, which is consistent with the recommendations made in the Cochrane Handbook (Deeks, 2019). As there were only two studies, heterogeneity could not be explored further by conducting subgroup analyses or meta‐regression. For the other outcomes, we were not able to conduct meta‐analyses because of the limited number of studies and the incompleteness of the reported data. Because of the same reasons, we were not able to assess reporting bias by using a funnel plot or conduct sensitivity analyses, as planned in the protocol.

## CHARACTERISTICS OF STUDIES

**Characteristics of included studies**

Arthur 1973

**Table:** 

**Methods**	Experimental: Non‐randomized controlled trial
**Participants**	30 older adults (mean age 77 years, range 55–90 years; 15 men and 15 women) residing in a nursing home, described to be ‘withdrawn, uncooperative, communicated very little, had few visitors, were preoccupied with increasing age and various illnesses, and generally manifested little desire to live’
**Interventions**	1. *Friendly visiting by the same volunteer each week* (*n* = 10) in which the older adult was visited by the same volunteer for 1.5 h per week for 10 weeks 2. *Friendly visiting by a different volunteer each week* (n = 10) in which the older adult was visited by a different volunteer for 1.5 h per week for 10 weeks 3. *No friendly visiting (comparator)* (*n* = 10) Volunteers were 10 undergraduate university students (median age 20 years, range 18–29 years; 5 men and 5 women) who received a training session to orient them to nursing homes, the aged population and their needs, ethical considerations, and activities commonly performed by volunteers.
**Outcomes**	Primary: none Secondary: *Life satisfaction* according to the original 20‐item Life Satisfaction Index A (LSI‐A): change score between post‐test (at the end of the 8th week) and pre‐test (before the program)
**Notes**	The LSI‐A, although self‐administering, was presented individually to each subject. All items were read to each subject individually. No raw data reported, only *p* values. Risk of bias items not applicable, see Supporting Information: Appendix 3 for ROBINS‐I assessment.

Bogat 1983

**Table:** 

**Methods**	Experimental: Non‐randomized controlled trial
**Participants**	26 older adults (≥62 years) who were on a waiting list of a Friendly visitor program in northern Chicago were randomly assigned to either of 2 experimental groups: 1. Friendly visiting 2. Network‐building visiting A non‐equivalent control group of 13 older adults from Chicago was identified by a nun who delivered communion to these persons during home visits. These older adults: Expressed desire for a friendly visitor but had no such contact at present Gave verbal consent to complete the pre‐ and post‐test questionnaires As the aim of the network‐building visiting condition goes beyond merely friendly visiting, data on this group of participants were not extracted.
**Interventions**	1. *Friendly visiting*: a relationship‐oriented visiting program, in which visitors provided weekly 1‐h visits over a period of 3 months. 2. *No friendly visiting* Volunteers were 13 advanced undergraduate university students in community psychology (age range 18–31 years, 5 men and 8 women) who received 3 1.5‐h training sessions before any contact. The training sessions (lecture and role play) focused on understanding one's role in a relationship, learning basic helping skills, trying out those skills. Weekly 1‐h supervision sessions during the 3‐month intervention phase were used to generate resources, strategies, and support for the 13 students.
**Outcomes**	Primary: ￮ Number of incoming and outgoing daily *telephone calls* for 1 week: change between post‐test and pre‐test ￮ Number of *visitors and* the number of *visits made* by the older adult each day for 1 week: change between post‐test and pre‐test ￮ *Networks* in which the older adults are currently participating (Network Survey 1—Current Networks) Secondary: ￮ *Life satisfaction* according to the original 20‐item Life Satisfaction Index A (LSI‐A): change between post‐test and pre‐test ￮ *Depression* according to the Depression Adjective Check List: change between post‐test and pre‐test
**Notes**	Pre‐test data for experimental groups were collected by the student visitor during the first visit. To control for student expectations and social desirability responses, post‐test data were collected by testers unknown to the older adults. Pre‐ and post‐test data for the control group were collected by the nun who delivered home communion. No raw data reported, only group means, *F*‐values and indication of statistical significance reported. Risk of bias items not applicable, see Supporting Information: Appendix 3 for ROBINS‐I assessment.

Calsyn 1984

**Table:** 

**Methods**	Experimental: Randomized controlled trial
**Participants**	58 non‐institutionalized socially isolated (as indicated by referral agencies such as Meals on Wheels and County Older Residents Program) older adults (mean age 76.77 years, 47 women and 11 men) living alone or with someone, were put in blocks based on their preferences regarding the visitor's race and/or gender and on the visitor's geographic preference, before being randomly assigned to either of 3 groups
**Interventions**	1. *Face‐to‐face visiting* (final *n* = 21): one of 21 (14 women and 7 men) trained volunteers (older volunteers referred by senior citizen organizations and undergraduate students enroled in a field placement course) provided visits to 1 isolated older adult once a week for a period of 12 weeks. The length of the visit varied from older adult to older adult and from week to week, but generally lasted about 1.5 h per visit. Most of the visiting time was spent in companionship activities, primarily talking about common interests. Visitors turned in a journal sheet on each of their visits with information on activities that occurred during the visits, information on any new problems and the volunteer's feelings about the visit. 2. *Phone visiting* (final *n* = 16) 3. *No treatment* (final n = 13): one face‐to‐face visitor at the end of the study As this systematic review only looks at face‐to‐face visiting, data concerning the phone visiting group were not extracted. Visitor training consisted of three 4‐h sessions: 1. Biological, psychological and social aspects of aging + ground rules for visiting (keeping appointments, types of activities, potential legal issues such as liability) 2. Learning and practicing communication skills (active listening approach) 3. Learning and practicing communication skills—part 2
**Outcomes**	Primary: none Secondary: *Life satisfaction* according to the 3rd version of the Life Satisfaction Index (=LSIZ) with 13 of the 20 items of the original LSIA: Post‐test data (±2 weeks after the last visit)
**Notes**	Personal history project (study 2) goes beyond mere friendly visiting and was therefore not extracted by the reviewers.

Risk of bias table

**Table:** 

Bias	Authors’ judgement	Support for judgement
Random sequence generation (selection bias)	Low risk	Block randomization was used
Allocation concealment (selection bias)	Unclear risk	No information provided
Blinding of participants and personnel (performance bias)	High risk	Participants: It was impossible to blind the participants, as the visiting is part of the intervention. This lack of blinding may have affected the results of the LSIZ (social desirability bias). Study personnel: It was impossible to blind the volunteers who deliver the intervention, as their visiting is the intervention. It is conceivable that the volunteers have influenced the older adults with regard to the LSIZ.
Blinding of outcome assessment (detection bias)	High risk	It was impossible to blind the participants who are also the outcome assessors, as the visiting is part of the intervention. This lack of blinding may have affected the results of the LSIZ (social desirability bias).
Incomplete outcome data (attrition bias)	Low risk	4 people in the phone condition dropped out of the program after only a few visits and 4 people did not complete the post‐test due to death or illness
Selective reporting (reporting bias)	Unclear risk	Insufficient information available (no study protocol, not clear that all the expected outcomes are included in the paper)
Other bias	Low risk	No indication

Haight 1988–1992

**Table:** 

**Methods**	Experimental: Randomized controlled trial Two publications on the same RCT: one in 1988 (short‐term effects of intervention) and one in 1992 (long‐term effects of intervention)
**Participants**	*1988* 60 homebound older adults (>50 years of age, 47 women) randomly selected from Meals‐On‐Wheels recipients and home health services lists (majority disabled) were randomly assigned to one of 3 groups (mean ages in the 3 groups ranging from 73 to 79 years). *1992* 35 participants that had survived 1 year later (26 women and 9 men, mean age 77 years)
**Interventions**	1. Structured life review therapy 2. Friendly visiting (*n* = 16 completing the study) in which the older adult was visited for 1 h for 6 consecutive weeks by a paid college student. During the visit, they discussed the weather, health problems, current events and TV shows. 3. No treatment (*n* = 19 completing the study): participants only underwent pre‐ and post‐testing As the aim of structured life review therapy differs from reminiscence therapy and the therapy must be administered by a professional, data concerning the first group were not extracted.
**Outcomes**	Primary: none Secondary: ￮ *Life satisfaction* according to the original 20‐item Life Satisfaction Index A (LSI‐A): ￭ 1988: change score between post‐test (8 weeks) and pre‐test ￭ 1992: post‐test data at 1 year ￮ *Psychological well‐being* according to the Affect‐Balance Scale (ABS): ￭ 1988: change score between post‐test (8 weeks) and pre‐test ￭ 1992: post‐test data at 1 year ￮ *Depression* according to the Self‐Rating Depression Scale (SDS): ￭ 1988: post‐test mean adjusted for pre‐test score ￭ 1992: post‐test data at 1 year
**Notes**	1988: Ivy's microcounseling skills were used for all visits in all groups. These skills guide the interviewer to respond to the client in an open and accepting manner. Testing was done by one data collector during the first and last visit, whereas friendly visits and life reviews were conducted by two other data collectors. 1992: One of the primary research assistants conducted the follow‐up study 1 year later and visited the participants at home for approximately 90 min. During the first 45 min, the subject and the research assistant visited and recalled their original acquaintance. No other form of reminiscing took place. Both the subject and the researcher stated they enjoyed the time spent catching up. The last 45 min of the visit were used to answer the questions from the outcome measures.

Risk of bias table

**Table:** 

Bias	Authors’ judgement	Support for judgement
Random sequence generation (selection bias)	Low risk	Participant names were drawn alternately from a master list by the data collectors. The subjects in each group were visited in the order in which their names were drawn. The first, second and third subject were asked to participate in the life review, friendly visiting and no‐treatment group, respectively.
Allocation concealment (selection bias)	Unclear risk	No information provided
Blinding of participants and personnel (performance bias)	High risk	Participants: It was impossible to blind the participants, as the visiting is part of the intervention. Although the authors mention in the 1988 publication that the measurements were performed by independent research assistants, this lack of blinding may have affected the results (social desirability bias). In addition, at the 1‐year post‐test measurement (in the 1992 publication), interviews were administered by a research assistant who was somewhat acquainted with all participants, as (s)he visited the older adults 1 year earlier. This increases the risk of social desirability bias. Personnel: It was impossible to blind the volunteers who deliver the intervention, as their visiting is the intervention. It is conceivable that the volunteers have influenced the older adults with regard to the test results. However, in 1988, the chief investigator and those doing the testing remained ignorant of group assignment until the scores were tallied. In contrast, in 1992, the visiting research assistant was no longer blinded to the intervention, which might have influenced the way (s)he interacted with the older adults during this follow‐up interview.
Blinding of outcome assessment (detection bias)	High risk	It was impossible to blind the participants who are also the outcome assessors, as the visiting is part of the intervention. This lack of blinding may have affected the results (social desirability bias).
Incomplete outcome data (attrition bias)	Low risk	1988: The authors state that rejection and dropout rates were equally distributed among groups (*n* = 3 in each group). Of these, one each from the control and experimental groups said their children did not want strangers visiting them. Others became ill, hospitalized, or moved away, and one subject died before completing the study. 1992: Drop‐out was substantial (51%) due to death, illness and increased frailty (e.g., Alzheimer's disease, distraught). However, when the scores of the dropouts on the four outcome measures of depression, life satisfaction, psychological well‐being, and activities of daily living were examined and compared with those scores of the survivors, there were only small differences between the dropouts and remaining subjects. The dropout group had lower pre‐test scores and improved their scores less on the 8‐week post‐test than the surviving group that remained in the study. This was true for members of all three treatment groups. However, because there were no significant differences between dropouts from the original study and survivors in this study, in terms of age and health, being a survivor should not have significant effects on the outcome measures.
Selective reporting (reporting bias)	Unclear risk	Insufficient information available (no study protocol, not clear that all the expected outcomes are included in the paper)
Other bias	Unclear risk	The visitors/data collectors were 3 paid college students. Therefore, it does not concern true volunteers delivering the visit. It is unclear if this may have impacted the friendly visiting itself.

Hautzinger 1992

**Table:** 

**Methods**	Experimental: Randomized controlled trial
**Participants**	39 institutionalized older adults (mean age 78.9 ± 9.2 years, range 59–98 years; 8 men and 31 women) living in private, church‐affiliated and municipal retirement homes were randomly assigned to one of 4 groups
**Interventions**	1. *Controlling the time, frequency and duration of visits* (*n* = 10): the visitor reminded the older adult at each visit ‘not to let me stay any longer than you want me to’. Shortly before leaving, the visitor asked the older adult when would be a good time to come back for another visit. 2. *Informed about the time of visits* (*n* = 9): at the end of each visit, the visitor informed the older adult when she would be coming back (on day X at time Y). 3. *Visited on the basis of a random schedule* (*n* = 9): the older adults were not given the opportunity to control either when a visitor came or how long she stayed. Nor were they notified when a visitor was coming (‘I decided to drop by and pay you a visit today’). No appointments for further visits were made. 4. *Not visited* (*n* = 11) 14 female Psychology students visited the residents once weekly for a period of 40–50 min for 9 weeks. Each was assigned an older adult in each visitation condition, and trained to carry out the experiment accurately. The visitor played a relatively passive role when interacting with the subject. All conversations were terminated with the visitor saying: ‘I really enjoyed talking to you’. Older adults were visited an average of 1.3 times per week.
**Outcomes**	Primary: none Secondary: ￮ *Mental health as assessed by a nurse* according to the NAR subtest of the Nuremberg Age Inventory: change between post‐test and pre‐test ￮ *Self‐rated mental health* measured via a mood scale (Befindlichkeits‐Skala, von Zerssen 1976) and a questionnaire on psychological and somatic complaints of older people (Hautzinger 1984): change between post‐test and pre‐test Given that the same outcome (mental health) is measured via multiple methods (clinician‐rated and self‐rated), and clinician‐rated outcome measures are considered more relevant than self‐reported measures (see protocol), the reviewers only extracted the clinician‐rated data.
**Notes**	Replication study of Schulz 1976

Risk of bias table

**Table:** 

Bias	Authors’ judgement	Support for judgement
Random sequence generation (selection bias)	Unclear risk	Although the authors mention that sealed envelopes were created before the study started (email conversation with Martin Hautzinger), they mention in the paper that ‘residents who were in close contact with one another or who lived next to each other were not assigned different conditions if they met the admission criteria and were willing to participate’. Therefore, it is unclear if this truly is a randomized study.
Allocation concealment (selection bias)	Low risk	no, sealed envelopes were used (email conversation with Martin Hautzinger)
Blinding of participants and personnel (performance bias)	Low risk	Participants: although it was impossible to blind the participants, as the visiting is part of the intervention, this lack of blinding will not have influenced the data on clinician‐rated mental health. Personnel: although it was impossible to blind the visitors, this lack of blinding will not have influenced the data on clinician‐rated mental health, as these were rated by a researcher who was not involved in the study and was blinded to the allocation of the participants.
Blinding of outcome assessment (detection bias)	Low risk	Data on clinician‐rated mental health, as these were rated by a researcher who was not involved in the study and was blinded to the allocation of the participants
Incomplete outcome data (attrition bias)	Low risk	No drop‐out occurred (email conversation with Martin Hautzinger)
Selective reporting (reporting bias)	Unclear risk	Insufficient information available (no study protocol, not clear that all the expected outcomes are included in the paper)
Other bias	Low risk	No indication

Kahlbaugh 2011

**Table:** 

**Methods**	Experimental: Non‐randomized controlled trial
**Participants**	36 older adults (mean age 82 ± 9.8 years, 4 men and 32 women) residing in independent living residential apartments. Of these, 28 were randomly assigned to either of 2 groups: 1. Visit + Wii (*n* = 16) 2. Visit + TV (*n* = 12) Resident directors recruited 7 participants willing to serve as the no visit control group.
**Interventions**	1. *Visit + Wii* in which the same undergraduate female research assistant visited an older adult to play a Wii game of their choice (everyone chose Wii bowling) for 1 h per week for 10 weeks 2. *Visit + TV* in which the same undergraduate female research assistant visited an older adult to watch television programs of their choice for 1 h per week for 10 weeks 3. *No visit*. These older adults completed all measures at Week 1 and Week 10.
**Outcomes**	Primary: *Loneliness* according to the UCLA scale version 3 (Russell, 1988): change score between first and final visit Secondary: ￮ *Positive and negative mood* according to the Positive and Negative Affect Scale (PANAS; Watson 1988): post‐test data ￮ *Life satisfaction* according to the original 20‐item Life Satisfaction Index A (LSI‐A)(Neugarten, 1961): post‐test data
**Notes**	The study authors did not report the results of multiple outcomes, although they are listed in the Methods section. The data were, however, provided kindly by the authors upon request (email communication with Patricia Kahlbaugh). Risk of bias items not applicable, see Supporting Information: Appendix 3 for ROBINS‐I assessment.

Keller 1988

**Table:** 

**Methods**	Experimental: Randomized controlled trial
**Participants**	81 homebound older adults (≥60 years (12 aged 60–69 years, 26 aged 70–79 years and 43 aged 80‐94 years), 64 women and 17 men, 73% living alone) in need of increased social contact (as indicated by homecare agencies) were randomly assigned to one of 3 groups: 1. Friendly visiting (*n* = 16) 2. Friendly visiting + delivery of health‐related community services (*n* = 41) 3. Control (*n* = 24) As this systematic review does not include visiting aimed at delivering information on community services, data concerning the second group were not extracted.
**Interventions**	1. *Friendly visiting program* in which older adults registered in the Retired Senior Volunteer Program provided weekly visits for a period of 12 weeks. Visitors participated in an orientation in the form of an intake interview. 2. *No visiting* of any kind
**Outcomes**	Primary: none Secondary: none
**Notes**	The outcome studied by the authors is knowledge of 8 community services: 1. Visiting nurses 2. Congregate meals 3. Home delivered meals 4. The Retired Senior Volunteer Program 5. Homemaker health aides 6. Telephone reassurance 7. Adult day care 8. Carrier alert.

Lawlor 2014

**Table:** 

**Methods**	Experimental: Randomized controlled trial
**Participants**	100 community‐dwelling older adults (>60 years) experiencing loneliness (score ≥3 on the De Jong Gierveld Loneliness Scale or answer ‘Yes’ to the question ‘Would you say that much of the time during the past week you felt lonely?’) were identified by people working with older people in the community (e.g., GPs, public health nurses, parish staff). They were randomly assigned to the intervention (*n* = 49) or control group (*n* = 51). Demographics of the group who completed the study (*n* = 88): Intervention: *n* = 40, 30 women and 10 men, median age 80 years (IQR: 9) Control: *n* = 48, 37 women and 11 men, median age 81.5 years (IQR: 13.5)
**Interventions**	1. *Friendly visiting program* in which volunteers (older adults themselves, >55 years) were matched to an intervention participant and provided 1‐h weekly home visits for 10 weeks over a ± 3‐month period. Initially the aim of these visits was to develop a rapport with the participant. The volunteer then encouraged the participant to identify a social connection they would like to make and that would be sustainable beyond the timeframe of the study. 2. *No friendly visiting*. Just three home visits for data collection at baseline, at the 1‐month and the 3‐month follow‐up time point. At the final data collection time point, 3 months, each control participant was offered an information booklet on services and activities for older people in their locality and a discussion with the member of the research team regarding what activity might suit them. All control participants were invited to a social event following the completion of the study. All volunteers attended 2 training sessions on: Role of the volunteer (including boundaries) Background to loneliness and social isolation Local services for older people Trouble shooting Communication skills Role play Confidentiality Volunteers were supported in their role.
**Outcomes**	Primary: ￮ *Loneliness* according to the De Jong Gierveld 11‐item Loneliness Scale: ￭ 1‐month total scores (adjusted for baseline values) ￭ 3‐month total scores (adjusted for baseline values) ￭ [Subscores on social loneliness and emotional loneliness were not extracted] ￮ *Social isolation* according to the 10‐item Lubben Social Network Scale: ￭ 1‐month total scores (adjusted for baseline values) ￭ 3‐month total scores (adjusted for baseline values) Secondary: ￮ *Depressive symptom experiencing* according to the 8‐item Center for Epidemiologic Studies ‐ Depression Scale (CES‐D 8) scale: ￭ 1‐month total scores (adjusted for baseline values) ￭ 3‐month total scores (adjusted for baseline values) ￭ [Subscores on items 5 (loneliness) and 7 (sadness were not extracted]
**Notes**	The study authors do not report the results of multiple secondary outcomes, although they are listed in the Methods section: Relevant to this systematic review: ￮ Hospital Anxiety and Depression Scale (HADS) Not relevant to this systematic review: ￮ Montreal Cognitive Assessment Scale (MOCA) ￮ Control, Autonomy, Self‐realization and Pleasure (CASP‐19) ￮ Pittsburgh Sleep Quality Index – item 6 ￮ Oslo Social Support Scale (OSS), since it concerns a subjective measurement of social support

Risk of bias table

**Table:** 

Bias	Authors’ judgement	Support for judgement
Random sequence generation (selection bias)	Low risk	Block randomization was conducted and a computer‐ generated random sequence list was used to randomly allocate participants
Allocation concealment (selection bias)	Low risk	Group allocation was concealed from both participants and the researchers until after baseline data collection was conducted
Blinding of participants and personnel (performance bias)	High risk	Participants: It was impossible to blind the participants, as the visiting is part of the intervention. Although the authors mention that a member of the research team collected data, this lack of blinding may have affected the results (social desirability bias). Personnel: It was impossible to blind the volunteers who deliver the intervention, as their visiting is the intervention. It is conceivable that the volunteers have influenced the older adults with regard to the test results.
Blinding of outcome assessment (detection bias)	High risk	It was impossible to blind the participants, who were also the outcome assessors, as the visiting is part of the intervention. Although the authors mention that a member of the research team collected data, this lack of blinding may have affected the results (social desirability bias).
Incomplete outcome data (attrition bias)	Unclear risk	The authors did not ask the participants about their specific reason for drop‐out (email conversation with Gillian Paul: ‘Control group: 1 person stated they didn't like being in the control group, the others did not wish to continue in the study, no specific reason was given. Intervention group: apart from the 5 with a reason (3 hospitalized, 1 deceased, 1 felt too unwell to participate) the others did not wish to continue in the study, no specific reason was given’). It is unclear if this may be connected with the outcomes measured.
Selective reporting (reporting bias)	High risk	The study authors do not report the results of multiple outcomes, although they are listed in the Methods section. The authors explained that ‘This was a short report for the funders reporting the significant findings’ and that they did no longer have access to the results (email conversation with Gillian Paul).
Other bias	Low risk	No indication

MacIntyre 1999

**Table:** 

**Methods**	Experimental: Randomized controlled trial
**Participants**	26 community elderly (68% women, mean age 80 years) thought to be lonely and socially isolated (as assessed by professional nurses of nursing services)
**Interventions**	1. *Friendly visiting program* in which trained community volunteers (undergraduate students studying gerontology) provided weekly visits (on average 3 h) for 6 weeks. Activities included walks around the house, talking, assisting with care activities, reading, writing letters and often just listening. The elderly indicated the visitors provided ‘company’ and gave them ‘something to do’. 2. *No friendly visiting*
**Outcomes**	Primary: none Secondary: *Life satisfaction* according to the LSIZ scale: change score between post‐test and pre‐test
**Notes**	

Risk of bias table

**Table:** 

Bias	Authors’ judgement	Support for judgement
Random sequence generation (selection bias)	Unclear risk	No information provided
Allocation concealment (selection bias)	Unclear risk	No information provided
Blinding of participants and personnel (performance bias)	High risk	Participants: It was impossible to blind the participants, as the visiting is part of the intervention. Although the authors mention that the measurements were performed by independent research assistants, this lack of blinding may have affected the results (social desirability bias). Personnel: It was impossible to blind the volunteers who deliver the intervention, as their visiting is the intervention. It is conceivable that the volunteers have influenced the older adults with regard to the test results.
Blinding of outcome assessment (detection bias)	High risk	It was impossible to blind the participants, who are also the outcome assessors, as the visiting is part of the intervention. Although the authors mention that the measurements were performed by independent research assistants, this lack of blinding may have affected the results (social desirability bias).
Incomplete outcome data (attrition bias)	Low risk	The authors clearly report that 4 participants (intervention *n* = 3, control *n* = 1) were unable to complete the questionnaire themselves and were therefore excluded from the study. There is no reason to assume that this was caused by the intervention.
Selective reporting (reporting bias)	Unclear risk	Insufficient information available (no study protocol, not clear that all the expected outcomes are included in the paper)
Other bias	Unclear risk	The older adults were also receiving professional and homemaking nursing services of the organization to assist in their personal needs, e.g., bathing, monitor medications, monitor mobility, preparation of meals, household chores, shopping… The authors did not correct for multiple comparisons Limited sample size (*n* = 22) and short time period (6 weeks)

McNeil 1991–1995

**Table:** 

**Methods**	Experimental: Randomized controlled trial Two publications on the same RCT: one in 1991 (short‐term effects of intervention) and one in 1995 (long‐term effects of intervention)
**Participants**	30 community‐dwelling older adults (≥60 years, mean 72.5 ± 6.9 years; 26 men and 4 women) with moderate levels of depressed mood (Beck Depression Inventory score 12‐24) but not receiving treatment from a mental health professional, not using sedatives or tranquilizers and not suicidal, were randomly assigned to one of 3 groups: 1. Accompanied walking 2. Home visit + conversation 3. Wait‐list control As the accompanied walking intervention consisted of walking with the visitor twice and walking alone once per week, data concerning the accompanied walking group were not extracted.
**Interventions**	1. *Home visit + conversation:* the same undergraduate psychology student provided visits to the older adult's home twice a week, increasing gradually in duration from 20 to 40 min over the 6‐week program. The visit consisted of casual conversation. It was made clear at the outset of the study that, although the student had taken several psychology courses, she was not a professional and could not help them to overcome personal problems in any specific therapeutic way. All older adults underwent a 4‐week termination period that involved the gradual reduction in the number of weekly contacts. 2. *Wait‐list control:* the older adults were told that their home visit would be delayed for 6 weeks.
**Outcomes**	Primary: none Secondary: ￮ 1991: *Depressive symptoms* according to the Beck Depression Inventory (BDI): Change score between post‐test and pre‐test (total BDI score). [BDI subscores on items 1–14 (psychological symptoms) and items 15–21 (somatic symptoms) were not extracted] ￮ 1995: *Psychological well‐being* according to the Memorial University of Newfoundland Scale of Happiness (MUNSH): Change over time
**Notes**	null

Risk of bias table

**Table:** 

Bias	Authors’ judgement	Support for judgement
Random sequence generation (selection bias)	Unclear risk	No information provided
Allocation concealment (selection bias)	Unclear risk	No information provided
Blinding of participants and personnel (performance bias)	Unclear risk	Insufficient information provided
Blinding of outcome assessment (detection bias)	Unclear risk	Insufficient information provided
Incomplete outcome data (attrition bias)	Low risk	There were no drop‐outs
Selective reporting (reporting bias)	Unclear risk	Insufficient information available (no study protocol, not clear that all the expected outcomes are included in the paper)
Other bias	Unclear risk	Very limited reporting

Mulligan 1978

**Table:** 

**Methods**	Experimental: Non‐randomized controlled trial
**Participants**	23 isolated older adults (≥65 years, mean age 77 years, 21 women and 2 men) living on the upper West Side of New York City in 1971–1972. This sample was obtained by knocking on doors and selecting people after some preliminary questioning relevant to their isolation. Based on their living area (which were both found comparable to the 1960 census concerning their demography), they were assigned to the intervention (*n* = 11) or control (*n* = 12) group.
**Interventions**	1. *Friendly visiting program* in which 1 of 5 pairs of trained community volunteers provided 1‐h‐long visits to 1 isolated older adult every 2 weeks for 6 months. The older adults were assessed at each visit. 2. *No friendly visiting*. Just one interview for data collection at the beginning and one at the end of the study.
**Outcomes**	Primary: *Social isolation* according to Past Month Isolation Index: ￮ Change scores between first and final (12th) visit; ￮ Change scores between first and 6‐month follow‐up visit. Secondary: *Presence of functional mental disorders* according to the Mental Status Schedule and the MSS‐Geriatric Supplement: Change scores between first and final (12th) visit
**Notes**	In the papers, two scales are mentioned, that is, the Adulthood Isolation Index and the Past Month Isolation Index. However, only one measure is reported. As social isolation is defined by the study authors as ‘the number of face‐to‐face contacts available to the person in the past month as assessed on a 5‐item index. Isolation was indicated by scores in the 0–2 range out of a possible score of 10’ and the Adulthood Isolation Index's possible scores range from 0 to 32 (instead of 0–10), the systematic reviewers assumed the authors reported the findings of the Past Month Isolation Index. Risk of bias items not applicable, see Supporting Information: Appendix 3 for ROBINS‐I assessment.

Reinke 1981

**Table:** 

**Methods**	Experimental: Randomized controlled trial
**Participants**	49 nursing home residents were randomly assigned to one of 3 groups: 1. Friendly visiting focusing on conversational interaction 2. Friendly visiting with conversational interaction and playing of cognitively challenging games 3. No treatment Demographics of group who completed the study (*n* = 39): 27 women and 12 men, mean age 79.45 ± 10.47 years (range: 59‐97 years), mean length of continuous residency in nursing homes 44.27 ± 36.09 months (range: 2–169 months)
**Interventions**	1. *Friendly visiting focusing on conversation*. Other social interaction (e.g., taking a walk, pasting photos into an album, making popcorn) were permissible if they did not resemble playing games. 2. *Friendly visiting with conversation and cognitive game playing*: at least one cognitive game (checkers, dominoes, triominoes, concentration game (card game involving memory), gin rummy, crossword puzzles, Mastermind) was played each visit in addition to the conversational component. 3. *No friendly visiting*: Waiting‐list control condition. Each nursing home resident was assigned 2 (of a total of 38) undergraduate student visitors who provided 1‐h long visits every week for 8 weeks. Therefore, each resident received a total of 16 visits between the pre‐ and post‐test. Each visitor was assigned to visit one resident in the conversation group and one in the conversation + games group. The visitors received 2 h of training concerning suggested ways of interacting with older adults and procedures for conducting each visit.
**Outcomes**	Primary: none Secondary: Post‐test scores residualized for pre‐test scores ￮ *Life satisfaction* according to the LSIA ￮ *Morale* according to the Philadelphia Geriatric Center Morale Scale
**Notes**	null

Risk of bias table

**Table:** 

Bias	Authors’ judgement	Support for judgement
Random sequence generation (selection bias)	Unclear risk	No information on the randomization procedure provided
Allocation concealment (selection bias)	Unclear risk	No information provided
Blinding of participants and personnel (performance bias)	High risk	Participants: it was impossible to blind the participants, as the visiting is part of the intervention. Although the authors state that ‘with almost all subjects, the experimenters were unaware of the condition in which the subjects were serving’, this lack of blinding may have affected the results (social desirability bias). Personnel: It was impossible to blind the volunteers who deliver the intervention, as their visiting is the intervention. It is conceivable that the volunteers have influenced the older adults with regard to the test results.
Blinding of outcome assessment (detection bias)	High risk	It was impossible to blind the participants, who are also the outcome assessors, as the visiting is part of the intervention. Although the authors state that ‘with almost all subjects, the experimenters were unaware of the condition in which the subjects were serving’, this lack of blinding may have affected the results (social desirability bias).
Incomplete outcome data (attrition bias)	Low risk	Ten of the subjects (conversation *n* = 4, conversation + games *n* = 3, control *n* = 3) did not complete the experiment for reasons of death (*n* = 1), psychiatric hospitalization (*n* = 2), severe physical illness (*n* = 1), severe lapse in mental status (*n* = 1), request to discontinue visitors (*n* = 1), and refusal to participate in post‐testing (*n* = 4). Not only do the authors provide a clear explanation, they also state that these subjects did not differ on other demographic or pretest data with the other study subjects.
Selective reporting (reporting bias)	Unclear risk	Insufficient information available (no study protocol, not clear that all the expected outcomes are included in the paper)
Other bias	High risk	All 49 residents expressed an interest in being visited. This may have biased the results. It is unclear which types of social interaction (e.g., taking a walk, pasting photos in an album, making popcorn) were used by the ‘conversation’ visitors. Therefore, it is unclear if the effects should be attributed to the conversation or to another type of social interaction. The results of the analyses in this article were questioned by one of the authors, who reanalysed the data in a second article (Denney, 1988). The other authors, Reinke and Holmes, replied to the comments of Denney in a third article (Reinke, 1988), pointing out errors made in the reanalyses and possible reasons for the differences in findings. Therefore, the results of this article are of questionable quality.

Schulz 1976–1978

**Table:** 

**Methods**	Experimental: Randomized controlled trial Two publications on the same RCT: one in 1976 (short‐term effects of intervention) and one in 1978 (long‐term effects of intervention)
**Participants**	42 institutionalized older adults (mean age 81.5 years, range 67–96 years; 6 men and 36 women) living in a private, church‐affiliated retirement home were randomly assigned to 1 of 4 groups: 1. Controlling the frequency and duration of visits 2. Informed about frequency and duration of visits 3. Visited on the basis of a random schedule 4. Not visited Regular contact with others in the home was usually limited to 2 or 3 close friends living in close proximity.
**Interventions**	1. *Controlling the frequency and duration of visits*. The visitor reminded the older adult at each visit ‘not to let me stay any longer than you want me to’. Shortly before leaving, the visitor asked the older adult when would be a good time to come back for another visit, and left his name and phone number, so the older adult could give him a call. Mean length of each visit: 50 min. 2. *Informed about frequency and duration of visits*. Older adults knew when to expect a visitor but were not given the opportunity to determine when a visitor came (‘I'll drop by to see you at …’) or how long he stayed (they were informed at the beginning of each meeting approximately how long the visit would last). Mean length of each visit: 49 min. 3. *Visited on the basis of a random schedule*. Older adults were not given the opportunity to control either when a visitor came or how long he stayed. Nor were they notified when a visitor was coming (‘I decided to drop by and pay you a visit today’). Mean length of each visit: 50.8 min. 4. *Not visited* 5 undergraduate students (1 man and 4 women) visited the residents. Each was assigned an older adult in each visitation condition, and trained to carry out the experiment accurately. The visitor played a relatively passive role when interacting with the subject. All conversations were terminated with the visitor saying: ‘I really enjoyed talking to you’. Older adults were visited an average of 1.3 times per week.
**Outcomes**	Primary: Obtained via questionnaires and interviews by the same researcher (introducing himself as a graduate student) before the start of the program and ±2 months after the initial interview and expressed as change scores: ￮ *Loneliness* as reported by the older adult in an interview in response to the question ‘What percentage of the time are you lonely?’ ￮ *Activity index*, as reported by the older adult in a questionnaire reflecting the frequency per week of number of visits to neighbours in the building, number of visits outside the building, number of times the building was left for activities other than visiting, number of club meetings attended, number of visits to church and number of phone calls made Secondary: ￮ *Zest for life* as rated by the activities director of the nursing home using a 9‐point Likert scale (ranging between ‘extremely enthusiastic about life’ and ‘completely hopeless’): ￭ 1976: after the program (±2 months after the initial interview with the older adults) ￭ 1978: 24‐, 30‐ and 42‐months follow‐up ￮ 1976: Obtained via questionnaires and interviews after the program: ￭ *Level of hope* according to the Wohlford Hope Scale, administered verbally ￭ *Happiness* as reported by the older adult in a questionnaire using a 9‐point Likert scale ￭ *Usefulness* as reported by the older adult in a questionnaire using a 9‐point Likert scale [As the authors did not report/analyse data on the 4 groups separately (the only analyses reported compared the no treatment + random groups to the predict + control groups), the reviewers were not able to extract data on these primary and secondary outcomes in the 1976 and 1978 publications. Activity, sociability, awareness and pleasantness scores were not extracted from the 1978 publication, as the outcomes were not clearly defined or described.]
**Notes**	Interpretation of the 42‐month follow‐up data must remain clouded. A fire killing several persons occurred shortly before these data were collected, and while none of the study participants were directly injured by the fire, all were inconvenienced by it and all suffered emotionally.

**Characteristics of excluded studies**

Aday 1991

**Table:** 

**Reason for exclusion**	Intervention: not just friendly visiting of the seniors by the students, but also other activities (visits to the elementary school, Christmas party, group session ‘painting to music’, farewell picnic).
Bowling 1989	
**Reason for exclusion**	Intervention: Study has used the ‘Social Network List’, a measure of the number of people who the person has contact with at least once a month. However, this does not indicate if the contact is face‐to‐face and what the nature of the contact is.
Butler 2006	
**Reason for exclusion**	Intervention: following types of assistance: companionship; rides to medical appointments, grocery stores, and banks; respite to caregivers; and the completion of small tasks and errands such as picking up medications or groceries
Clarke 1992	
**Reason for exclusion**	Intervention: Much more than friendly visiting (‘The type of intervention carried out fell into one of five main categories, some examples of which are given in parentheses: social and social services (arranging visits to another elderly person or outings with voluntary organizations; Meals on Wheels; home help); financial (liaising with local administrative offices for rates, benefits, or collecting pensions); housing (installing safety chains and spy holes onto doors, arranging for volunteers to do gardening or decorating); nursing (referral for assessment for a bath nurse or requesting advice from the continence nurse); and medical (assistance in making an appointment to see the family doctor or informal liaison/discussion with the general practitioner)’.)
Corella 2012	
**Reason for exclusion**	Design: thesis containing research proposal. Director of the School of Social Work Nancy Meyer‐Adams confirmed that the project was never executed.
Davis 2009	
**Reason for exclusion**	Design: No quantitative data available on the effectiveness of befriending part of the programme
Dean 1998	
**Reason for exclusion**	Design: the reviewers discussed the summary report and concluded that there would probably not be quantitative data on impact in the full report (otherwise they would have been included in the summary report). Therefore, the full report was not ordered.
Debonera 2020	
**Reason for exclusion**	Intervention: focus lies on mindful breathing
Dooley 1990	
**Reason for exclusion**	Intervention: students assisted with cooking, laundry, snow removal, indoor/outdoor maintenance, shopping or other errands
Doyle 2021	
**Reason for exclusion**	Population: clinically depressed people
Falkowski 2014	
**Reason for exclusion**	Intervention: the reviewers contacted Paul Falkowski. He confirmed that there were no data on the impact of friendly visits alone.
Goldman 2003	
**Reason for exclusion**	Design: descriptive, no results. The reviewers tried to contact the author, but were not able to retrieve contact details.
Green 1987	
**Reason for exclusion**	Population: no information on the age of the participants is available; only ‘elderly’
Grond 1984	
**Reason for exclusion**	Intervention: delivered by students who require their own professional skills to do so (students in social work and nursing)
Harris 2005	
**Reason for exclusion**	Intervention: No separate information on volunteering that only consists of paying friendly visits to other older people.
Hart 2016	
**Reason for exclusion**	Intervention: personal communication with Derek Willis revealed that the intervention consisted of visits + telephone calls
Jacob 2020	
**Reason for exclusion**	Design: no quantitative data on effectiveness of the programme. The reviewers tried to contact the study authors, but were not able to retrieve contact details.
Kildemoes 1977	
**Reason for exclusion**	Design: no quantitative data. The reviewers considered it very unlikely that these would be available.
Kim 2012	
**Reason for exclusion**	Intervention: much more than just friendly visiting (e.g., checking health status, performing hand massage, performing indoor exercise together, informing public health nurses of emergency health problems)
Lloyd‐Sherlock 2017	
**Reason for exclusion**	Intervention: Volunteers provide domiciliary care services, such as assistance with eating, exercise and taking medicine, and liaising with local health workers.
Martin 2012	
**Reason for exclusion**	Intervention: much more than friendly visiting. ‘The volunteers’ support helps these elders remain independent: volunteers assist elders with errands outside the home (58%), support their medical needs (39%), help with home maintenance (33%), and advocate for elders when necessary (30%)’.
Martina 2006	
**Reason for exclusion**	Intervention: Friendship enrichment program consists of lessons that include theory, practice in skills that are important in friendship, role‐playing of difficult social situations and homework
McHugh Power 2016	
**Reason for exclusion**	Intervention: surpasses the aim of tackling loneliness and social isolation
McWilliams 2008	
**Reason for exclusion**	Intervention: the objective of the program goes further than socialization; it also aims to improve functional capacity in the seniors, increased understanding of their health status, perceived quality of care
Mulligan 1974	
**Reason for exclusion**	Other: covered by publication of Mulligan 1977–1978
NCT00105378	
**Reason for exclusion**	Intervention: delivered by a healthcare professional (geriatric nurse)
NCT01408654	
**Reason for exclusion**	Intervention: combination of telephone and face‐to‐face visits. The reviewers contacted the study authors. They confirmed that there were no dyades without telephone calls.
NCT01829594	
**Reason for exclusion**	Intervention: main purpose is physical/medical improvement
NCT01842984	
**Reason for exclusion**	Intervention: (partly) group intervention
NCT02308696	
**Reason for exclusion**	Intervention: broader than friendly visiting. To make this assessment, the reviewers contacted the study investigators (Elizabeth Jacobs). The personal communication and another peer‐reviewed paper describing the intervention (doi:10.1001/jamanetworkopen.2020.30090) enabled to exclude the study.
NCT03343483	
**Reason for exclusion**	Intervention: Senior Companion program entails visits that have multiple goals, e.g., assisting with meal prep and nutrition, accompany shopping, assist with reading or writing, help pay bills, assist with medication etc.
NCT03345862	
**Reason for exclusion**	Intervention: partly telephone intervention
NCT03593967	
**Reason for exclusion**	Intervention: much more than just friendly visiting
NCT03989284	
**Reason for exclusion**	Intervention: goal of the intervention exceeds friendly visiting
NCT04336553	
**Reason for exclusion**	Design: qualitative study (information obtained from study investigator Erika Johansson)
Noguchi 2014	
**Reason for exclusion**	Intervention: exceeds the aim of tackling loneliness and social isolation (financial advice, health advice, etc.)
Oppikofer 2002	
**Reason for exclusion**	Population: the reviewers contacted Sandra Oppikofer twice (6/7/2020 and 2/11/2020) to inform if data were available on the subgroup of older adults with mild cognitively impairment. No response was obtained. Therefore, the study population consisted also of people with dementia.
Oppikofer 2010	
**Reason for exclusion**	Population: the reviewers contacted Sandra Oppikofer twice (6/7/2020 and 2/11/2020) to inform if data were available on the subgroup of older adults with mild cognitively impairment. No response was obtained. Therefore, the study population consisted also of people with dementia.
Rachasrimuang 2018	
**Reason for exclusion**	Intervention: involved much more than friendly visiting to alleviate loneliness and social isolation, as confirmed by email conversation with Sarawut Rachasrimuang (e.g., giving tips on fall prevention, exercise, hobbies, healthy foods, etc.)
Rantanen 2015	
**Reason for exclusion**	Intervention: focus lies on outdoor activities (not friendly talking, playing games and/or reminiscing)
Saito 2012	
**Reason for exclusion**	Intervention: Group‐based educational, cognitive, and social support program (4 2‐h sessions)
Schwei 2021	
**Reason for exclusion**	Intervention: The reviewers contacted Rebecca Schwei. She confirmed that the majority of the peer‐to‐peer support was done face‐to‐face, but that the volunteers also sometimes did sunshine calls where they would call and check in on their partner. Therefore, not only face‐to‐face contact.
Sillanmaki 2014	
**Reason for exclusion**	Intervention: included various activities such as visits places of interest, walking, participation in cultural events and daily taking care of things; focus on outdoor activities
Stevens 2000	
**Reason for exclusion**	Intervention: Program consists of 12 lessons, consisting of theory on the topic, practice in skill that are important in friendship, role playing and homework assignments + opportunity to discuss their personal experiences.
Sørensen 1988	
**Reason for exclusion**	Intervention: Intervention was aimed at relieving unmet medical (and social) needs
van den Elzen 2006	
**Reason for exclusion**	Intervention: Elderly support home visits where services delivered mainly included shopping, walking, doing chores and talking. The reviewers concluded this was more than just friendly visiting.
van Haastregt 2000	
**Reason for exclusion**	Intervention: Preventive home visits are ‘aimed at multidimensional medical, functional, psychosocial, and environmental evaluation of their problems and resources’
Wilson 2010	
**Reason for exclusion**	Intervention: Control group received a friendly weekday greeting. No control group that did not receive any visit.
Wright 1977	
**Reason for exclusion**	Design: no quantitative data on effectiveness. The reviewers wanted to contact Ruth Bennett to ask if there were other papers related to this study, but could not find contact details.

**Characteristics of studies awaiting classification**

Al‐Khazraji 1974

**Table:** 

**Methods**	Experimental study (no details available)
**Participants**	Older adults previously institutionalized for mental health problems
**Interventions**	Support by a lay volunteer (no details available). Comparison not known.
**Outcomes**	Not known
**Notes**	Full‐text not available from KU Leuven library collection or external university libraries requested by KU Leuven

Cattan 2002

**Table:** 

**Methods**	Systematic review, survey, case studies
**Participants**	Older people
**Interventions**	Health promotion interventions
**Outcomes**	Not known
**Notes**	Full‐text not available. The reviewers contacted Mima Cattan to ask if the research in this PhD thesis was covered by her other papers, but received an out‐of‐office due to retirement.

Cattan 2003

**Table:** 

**Methods**	Case study interviews, focus groups
**Participants**	Project staff and older people
**Interventions**	Not known
**Outcomes**	Not known
**Notes**	Full‐text not available. The reviewers contacted Mima Cattan to ask if this research also included quantitative data (from abstract it appears to be only qualitative), but received an out‐of‐office due to retirement.

ChiCTR1800017915

**Table:** 

**Methods**	Observational study
**Participants**	Older adults in the community (≥60 years)
**Interventions**	Influencing factors of social isolation, and their interaction
**Outcomes**	Social isolation, frailty, cognition, family support, social support
**Notes**	Authors were contacted twice (9/4/2021 and 16/4/2021) to find out the study purpose. No response obtained.

CTRI/2018/01/011466

**Table:** 

**Methods**	Observational: Cross‐sectional study
**Participants**	Older people (≥60 years) living in the Ernakulam district of Kerala (India)
**Interventions**	Coping strategies to overcome loneliness
**Outcomes**	Loneliness
**Notes**	Authors were contacted twice (9/4/2021 and 19/4/2021) to find out what was included in the quantitative aspect of the study. No response was obtained.

NCT03405675

**Table:** 

**Methods**	Observational prospective cohort study
**Participants**	Community‐dwelling older adults (≥55 years) in the Singapore areas of Geylang, Aljunied, MacPherson, Marine Parade, Bedok, Bukit Merah and Jurong
**Interventions**	Biological, clinical, psychosocial and behavioural predictors of health status
**Outcomes**	Primary: dementia and mild cognitive impairment, cognitive functioning ability, frailty, depressive symptoms and diagnosis, successful ageing Secondary: self‐reported independent functioning, physical performance, health services utilization, quality of life, mortality, post‐bronchodilation spirometry
**Notes**	Authors were contacted twice (2/7/2020 and 2/11/2020) to find out if social contact will be studied. No response was obtained.

NCT03695133

**Table:** 

**Methods**	Experimental: Randomized controlled trial
**Participants**	Community‐dwelling older women (≥65 years) that experience high levels of loneliness
**Interventions**	Intervention: ∘ Group‐based interventions: physical activity, sightseeing, picnics, theater, cinema, group education ∘ Individual interventions: interventions in the Omaha System Nursing Interventions Scheme Control: no intervention
**Outcomes**	Primary: loneliness Secondary: physical activity, health status perception, social inclusion, perceived social support, well‐being, healthy life style behaviour
**Notes**	Authors were contacted twice (9/4/2021 and 19/4/2021) to find out if it there is a group that receives one‐to‐one interventions or if all groups receive group interventions. No response was obtained.

NCT04224038

**Table:** 

**Methods**	Observational: Prospective cohort study
**Participants**	Adults (≥30 years) living in the Occitania Region (France)
**Interventions**	The Inspire Bio‐resource Research Platform for Healthy Aging. Several follow‐up visits will be undertaken during the 10‐year time frame of this project.
**Outcomes**	Primary: data collection of biological, clinical and digital resources + biospecimens Secondary: ∘ Identification of biomarkers of aging through a comprehensive biobank ∘ Intrinsic capacity ∘ Basic and instrumental activities of daily living ∘ Nutritional status and diet ∘ Lifestyle ∘ Physical activity ∘ Visceral pain ∘ Participant‐reported cognition ∘ Participant‐reported mobility ∘ Participant‐reported fatigue ∘ Participant‐reported social isolation ∘ Oral health ∘ Physical performance ∘ Cognitive function ∘ Skin elasticity ∘ Muscle strength ∘ Oxygen consumption and aerobic power
**Notes**	Authors were contacted twice (2/7/2020 and 2/11/2020) to find out if social contact will be used as an intervention. No response was obtained.

**Characteristics of ongoing studies**

Ninesling 2018

**Table:** 

**Study name**	Effects of a Volunteer‐based Lunch Program on Feelings of Loneliness in Elders
**Methods**	Experimental: Randomized controlled trial
**Participants**	Older adults (≥60 years) with feelings of loneliness
**Interventions**	1. *Volunteer‐based lunch program* where trained medical students bring and share a 1‐h lunch once a week for 6 weeks at the home of an older adult 2. *No intervention*: older adults receive daily meals from Meals on Wheels
**Outcomes**	Primary: *Loneliness* according to the Revised UCLA scale: Change score between pre‐ and post‐intervention Secondary: ∘ *Depressive symptom experiencing* according to the Patient Health Questionnaire‐9 scale ∘ *Feelings of anxiety* according to the General Anxiety Disorder 7‐item scale
**Starting date**	October 15, 2018
**Contact information**	Lucy Guerra
**Notes**	Although this is a meal delivery program, the main aim is to decrease feelings of loneliness in older adults

## SUMMARY OF FINDINGS TABLES

**Table 1:** Summary of findings. Short‐term effects of friendly visiting by a volunteer compared to no friendly visiting for reducing loneliness and social isolation in older adults.

**Patient or population:** older adults **Setting:** any **Intervention:** friendly visiting by a volunteer **Comparison:** no friendly visiting by a volunteer						

	Anticipated absolute effects* (95% CI)					
Outcomes	Risk with no friendly visiting by a volunteer	Risk with friendly visiting by a volunteer	Relative effect (95% CI)	No of participants (studies)	Certainty of the evidence (GRADE)	Comments
Loneliness	The mean loneliness was **6.7** (De Jong Gierveld 11‐item Loneliness Scale (scores 0–11): 0–2: not lonely 3–8: moderately lonely 9–10: severely lonely 11: very severely lonely)	MD **1.1 lower** (2.1 lower to 0.1 lower)	‐	88 (1 RCT)^1^	⨁◯◯◯ Very low^a^^,^ b	The evidence is very uncertain about the effect of friendly visiting by a volunteer on loneliness.
	The mean increase in loneliness between the first and final visit was **3.0** (UCLA scale version 3 (scores 20–80): 20: not lonely at all 80: as lonely as possible)	MD **2.84 lower** (10.0 lower to 4.3 higher)	‐	35 (1 non‐RCT)^2^	⨁◯◯◯Very low^b^^,^ c	
Social isolation	The mean social isolation was **21.5** (Lubben Social Network Scale (scores 0– 50): ≤20: small social network 21–25: moderate small social network 26–30: moderate large social network ≥31: large social network)	MD **2.2 higher** (0.05 lower to 4.5 higher)	‐	88 (1 RCT)^1^	⨁◯◯◯Very low^a^^,^ b	The evidence is very uncertain about the effect of friendly visiting by a volunteer on social isolation
	3/4 effects favoured friendly visiting			46 (2 non‐RCTs)^3^^,^ 4	⨁◯◯◯Very low^c^^,^ d	
Depressive symptom experiencing	2/2 effects favoured friendly visiting			143 (3 RCTs)^1^^,^ 5 ^,^ 6	⨁◯◯◯Very low^a^^,^ d	The evidence is very uncertain about the effect of friendly visiting by a volunteer on depressive symptom experiencing.
Life satisfaction	3/5 effects favoured friendly visiting			130 (4 RCTs)^5^^,^ 7 ^,^ 8 ^,^ 9	⨁◯◯◯Very low^a^^,^ d	The evidence is very uncertain about the effect of friendly visiting by a volunteer on life satisfaction.
	1/3 effects favoured friendly visiting			89 (3 non‐RCTs)^2^^,^ 3 ^,^ 10	⨁◯◯◯Very low^c^^,^ d	
Mental health	2/5 effects favoured friendly visiting			133 (4 RCTs)^5^^,^ 6 ^,^ 9 ^,^ 11	⨁◯◯◯Very low^a^^,^ d	The evidence is very uncertain about the effect of friendly visiting by a volunteer on mental health.
	2/2 effects favoured friendly visiting			57 (2 non‐RCTs)^2^^,^ 4	⨁◯◯◯Very low^b^^,^ c	
*The risk in the intervention group (and its 95% confidence interval) is based on the assumed risk in the comparison group and the **relative effect** of the intervention (and its 95% CI). **CI:** confidence interval; **MD:** mean difference						
**GRADE Working Group grades of evidence. High certainty:** we are very confident that the true effect lies close to that of the estimate of the effect. **Moderate certainty:** we are moderately confident in the effect estimate: the true effect is likely to be close to the estimate of the effect, but there is a possibility that it is substantially different. **Low certainty:** our confidence in the effect estimate is limited: the true effect may be substantially different from the estimate of the effect. **Very low certainty:** we have very little confidence in the effect estimate: the true effect is likely to be substantially different from the estimate of effect.						

^a^ Downgraded two levels for very serious limitations in study design (RoB assessment).

^b^ Downgraded one level for serious imprecision: limited sample sizes.

^c^ Downgraded two levels for very serious risk of bias (serious in ROBINS‐I assessment).

^d^ Downgraded one level for serious imprecision: limited sample sizes and lack of data.

^1^ Lawlor, 2014.

^2^ Kahlbaugh, 2011.

^3^ Bogat, 1983.

^4^ Mulligan, 1978.

^5^ Haight, 1988–1992.

^6^ McNeil, 1991–1995.

^7^ Calsyn, 1984.

^8^ MacIntyre, 1999.

^9^ Reinke, 1981.

^10^ Arthur, 1973.

^11^ Hautzinger, 1992.

## DATA AND ANALYSES

**1 Friendly visiting versus no friendly visiting**

**Table:** 

**Outcome or Subgroup**	**Studies**	**Participants**	**Statistical Method**	**Effect Estimate**
1.1 Short‐term loneliness (non‐RCTs)	1	Mean Difference (IV, Random, 95% CI)	No totals	
1.2 Long‐term depressive symptom experiencing (RCTs)	1	Mean Difference (IV, Random, 95% CI)	No totals	
1.3 Short‐term life satisfaction (RCTs)	2	56	Mean Difference (IV, Random, 95% CI)	2.35 [‐4.86, 9.56]
1.4 Short‐term life satisfaction (non‐RCTs)	1		Mean Difference (IV, Random, 95% CI)	No totals
1.5 Long‐term life satisfaction (RCTs)	1	Mean Difference (IV, Random, 95% CI)	No totals	
1.6 Short‐term mental health (RCTs)	1	Mean Difference (IV, Random, 95% CI)	No totals	
1.7 Short‐term positive mood (non‐RCTs)	1	Mean Difference (IV, Random, 95% CI)	No totals	
1.8 Short‐term negative mood (non‐RCTs)	1	Mean Difference (IV, Random, 95% CI)	No totals	
1.9 Long‐term mental health (RCTs)	1	Mean Difference (IV, Random, 95% CI)	No totals	


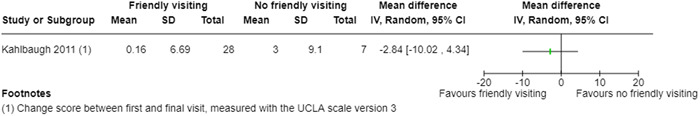



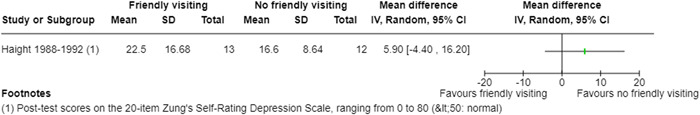



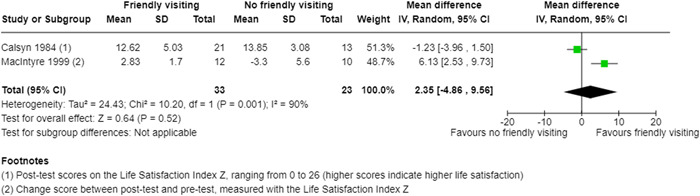



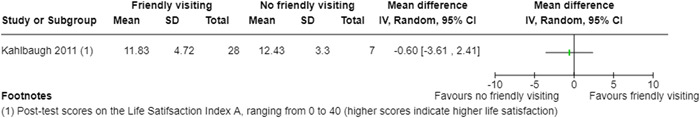



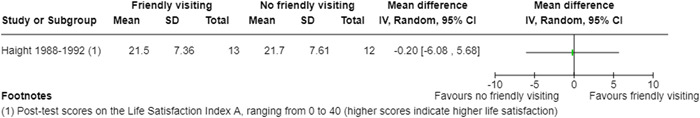



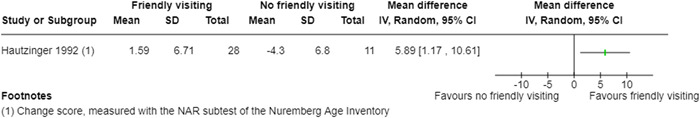



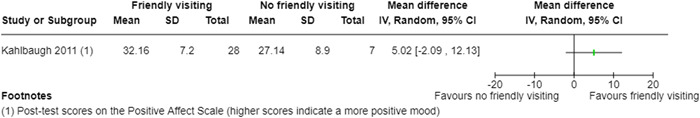



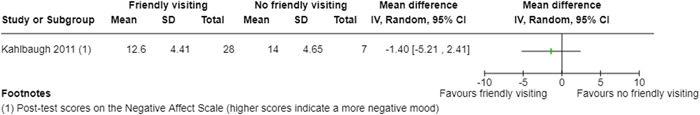



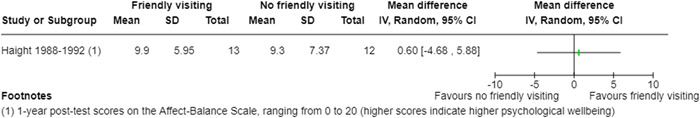


## SOURCES OF SUPPORT

**Internal sources**

- Belgian Red Cross, Belgium
  This systematic review is funded by the Foundation for Scientific Research of the Belgian Red Cross.

**External sources**

- None

## Supporting information

Supporting information.
