# Supplementary material for: Friendly visiting by a volunteer for reducing loneliness or social isolation in older adults: A systematic review
Source: Campbell Syst Rev. 2023 Nov 30;19(4):e1359. doi: 10.1002/cl2.1359 (PMC10688573; doi:10.1002/cl2.1359)
Supplement: Supplementary file 1 — Supporting information. [file CL2-19-e1359-s001.docx]

# Appendices

## 1 Search strategies

**MEDLINE (PubMed)**

#1 “Aged”[Mesh] OR elderly[TIAB] OR “Retirement”[Mesh] OR retire*[TIAB] OR pension*[TIAB] OR “old people”[TIAB] OR “older adults”[TIAB] OR “older people”[TIAB] OR ((resident[TIAB] OR residents[TIAB]) AND (“retirement home”[TIAB] OR “retirement homes”[TIAB] OR “nursing home”[TIAB] OR “nursing homes”[TIAB]))

#2 "Volunteers"[Mesh] OR volunt*[TIAB] OR “friendly visitor”[TIAB] OR “friendly visitors”[TIAB] OR “friendly visiting”[TIAB] OR “friendly visit”[TIAB] OR “friendly visits”[TIAB] OR befriending[TIAB]

#3 Visit*[TIAB]

#4 “Social Isolation”[Mesh:NoExp] OR “Social isolation”[TIAB] OR “Loneliness”[Mesh] OR lonel*[TIAB] OR “Depression”[Mesh] OR depression[TIAB] OR depressive symptom*[TIAB] OR “Life satisfaction”[TIAB] OR “Mental health”[Mesh] OR “mental health”[TIAB]

#5 (#1 AND #2 AND #3 AND #4)

**Embase (Elsevier)**

#1 ‘aged’/exp OR elderly:ab,ti OR ‘retirement’/exp OR ‘pensioner’/exp OR retire*:ab,ti OR pension*:ab,ti OR ‘old people’:ab,ti OR ‘older adults’:ab,ti OR ‘older people’:ab,ti OR ‘nursing home patient’/exp OR ((resident:ab,ti OR residents:ab,ti) AND (‘retirement home’:ab,ti OR ‘retirement homes’:ab,ti OR ‘nursing home’:ab,ti OR ‘nursing homes’:ab,ti))

#2 ‘Voluntary worker’/exp OR volunt*:ab,ti OR ‘friendly visitor’:ab,ti OR ‘friendly visitors’:ab,ti OR ‘friendly visiting’:ab,ti OR ‘friendly visit’:ab,ti OR ‘friendly visits’:ab,ti OR befriending:ab,ti

#3 Visit*:ab,ti

#4 ‘Social isolation’/de OR ‘Social isolation’:ab,ti OR ‘Loneliness’/exp OR lonel*:ab,ti OR ‘Depression’/exp OR depression:ab,ti OR (depressive NEXT/1 symptom*):ab,ti OR ‘life satisfaction’:ab,ti OR ‘Mental health’/exp OR ‘mental health’:ab,ti

#5 (#1 AND #2 AND #3 AND #4)

**The Cochrane Library (CENTRAL and CSDR, Wiley)**

#1 [mh “Aged”] OR elderly:ti,ab,kw OR [mh “Retirement”] OR retire*:ti,ab,kw OR pension*:ti,ab,kw OR “old people”:ti,ab,kw OR “older adults”:ti,ab,kw OR “older people”:ti,ab,kw OR ((resident:ti,ab,kw OR residents:ti,ab,kw) AND (“retirement home”:ti,ab,kw OR “retirement homes”:ti,ab,kw OR “nursing home”:ti,ab,kw OR “nursing homes”:ti,ab,kw))

#2 [mh “Volunteers”] OR volunt*:ti,ab,kw OR “friendly visitor”:ti,ab,kw OR “friendly visitors”:ti,ab,kw OR “friendly visiting”:ti,ab,kw OR “friendly visit”:ti,ab,kw OR “friendly visits”:ti,ab,kw OR befriending:ti,ab,kw

#3 Visit*:ti,ab,kw

#4 [mh “Social Isolation”] OR “Social isolation”:ti,ab,kw OR [mh “Loneliness”] OR lonel*:ti,ab,kw OR [mh “Depression”] OR depression:ti,ab,kw OR (depressive NEXT symptom*):ti,ab,kw OR “life satisfaction”:ti,ab,kw OR [mh “Mental health”] OR “mental health”:ti,ab,kw

#5 (#1 AND #2 AND #3 AND #4)

**PsycNet (PsycInfo and PsycArticles) - Basic search**

(it=(“Aged (Attitudes Toward)” OR Ageism OR Aging OR “Aging (Attitudes Toward)” OR “Aging in Place” OR “Physiological Aging” OR Retirement OR “Retirement communities”) OR ab=(elderly OR retire* OR pension* OR “old people” OR “older adults” OR “older people” OR ((resident OR residents) AND (“nursing home” OR “nursing homes” OR “retirement home” OR “retirement homes”))) OR KEYWORDS=(elderly OR retire* OR pension* OR “old people” OR “older adults” OR “older people” OR ((resident OR residents) AND (“nursing home” OR “nursing homes” OR “retirement home” OR “retirement homes”)))) AND (it=(Volunteers) OR ab=(volunt* OR “friendly visitor” OR “friendly visitors” OR “friendly visiting” OR “friendly visit” OR “friendly visits” OR befriending) OR KEYWORDS=(volunt* OR “friendly visitor” OR “friendly visitors” OR “friendly visiting” OR “friendly visit” OR “friendly visits” OR befriending)) AND (ab=(visit*) OR KEYWORDS=(visit*))
AND (it=(Loneliness OR “Patient Seclusion” OR “Social Isolation” OR “Major depression” OR “Depression (Emotion)” OR “Life Satisfaction” OR “Mental Health”) OR ab=(“social isolation” OR lonel* OR depression OR “depressive symptom” OR “depressive symptoms” OR “life satisfaction” OR “mental health”) OR KEYWORDS=(“social isolation” OR lonel* OR depression OR “depressive symptom” OR “depressive symptoms” OR “life satisfaction” OR “mental health”))

**ProQuest Sociology - Advanced search**

#1 MAINSUBJECT.EXACT("Older people") OR MAINSUBJECT.EXACT(“Aging”) OR MAINSUBJECT.EXACT("Retirement") OR MAINSUBJECT.EXACT(“Retirees”) OR MAINSUBJECT.EXACT(“Pensions”) OR MAINSUBJECT.EXACT(“Old age benefits”) OR MAINSUBJECT.EXACT(“Retirement homes”) OR MAINSUBJECT.EXACT(“Nursing homes”) OR ab,ti(elderly) OR ab,ti(retire*) OR ab,ti(pension*) OR ab,ti(“old people”) OR ab,ti(“older adults”) OR ab,ti(“older people”) OR ((ab,ti(resident) OR ab,ti(residents)) AND (ab,ti(“retirement home”) OR ab,ti(“retirement homes”) OR ab,ti(“nursing home”) OR ab,ti(“nursing homes”)))

#2 MAINSUBJECT.EXACT("Volunteers") OR ab,ti(volunt*) OR ab,ti(“friendly visitor”) OR ab,ti(“friendly visitors”) OR ab,ti(“friendly visiting”) OR ab,ti(“friendly visit”) OR ab,ti(“friendly visits”) OR ab,ti(befriending)

#3 ab,ti(visit*)

#4 MAINSUBJECT.EXACT("Loneliness") OR ab,ti(lonel*) OR MAINSUBJECT.EXACT(“Mental depression”) OR ab,ti(depression) OR ab,ti(“depressive symptom”) OR ab,ti(“depressive symptoms”) OR ab,ti(“life satisfaction”) OR MAINSUBJECT.EXACT(“Mental health”) OR ab,ti(“mental health”) OR MAINSUBJECT.EXACT(“Social isolation”) OR ab,ti(“social isolation”)

#5 (#1 AND #2 AND #3 AND #4; uncheck 'Peer reviewed' box)

**Social Sciences Citation Index 1956-present (Web of Science)**

#1 Topic: “elderly” OR “aged” OR “retire*” OR “pension*” OR “old people” OR “older adults” OR “older people” OR ((“resident” OR “residents”) AND (“nursing home” OR “nursing homes” OR “retirement home” OR “retirement homes”))

#2 Topic: “volunt*” OR “friendly visitor” OR “friendly visitors” OR “friendly visiting” OR “friendly visit” OR “friendly visits” OR “befriending”

#3 Topic: “visit*”

#4 Topic: “social isolation” OR “lonel*” OR “depression” OR “depressive symptom*” OR “life satisfaction” OR “mental health”

#5 (#1 AND #2 AND #3 AND #4)

**ClinicalTrials.Gov - Advanced search**

- Other terms: (visit OR visits OR visiting OR befriending) AND (loneliness OR lonely OR ((social OR socially) AND (isolation OR isolated)))
- Age Group: check boxes Adult (18-64) and Older Adult (65+)

**WHO International Clinical Trials Registry Platform**

Lonel* OR social isolat* OR socially isolat*

**Grey Literature Report (www.greylit.org)**

Search 1: Loneliness
Search 2: Social isolation

**OpenGrey (www.opengrey.eu)**

(visit* OR befriending) AND (lonely OR loneliness OR (social* AND isolat*))

**Campaign to end loneliness - Webpage screened**

www.campaigntoendloneliness.org/useful-reports-and-publications/

**Age UK - Webpages screened**

- www.ageuk.org.uk/our-impact/policy-research/loneliness-research-and-resources/
- www.ageuk.org.uk/our‐impact/policy‐research/publications/ (reports and briefings, consultation responses, evaluation reports)

**No isolation - Webpage screened**

www.noisolation.com/global/research/

**Eén tegen Eenzaamheid - Webpages screened**

- www.eentegeneenzaamheid.nl/over-eenzaamheid/over-een-tegen-eenzaamheid/
- www.rijksoverheid.nl/onderwerpen/eenzaamheid/documenten

**Friends for Good (www.friendsforgood.org.au)**

"Resources" screened

**Centre for Ageing Better**

All publications screened via www.ageing‐better.org.uk/publications

**International Longevity Centre UK - Reports (https://ilcuk.org.uk/reports/), search by Keyword**

Search 1: Loneliness
Search 2: Social isolation

**WHO Aging and life-course program - Screened all publications**

www.who.int/health-topics/ageing#tab=tab_1

**National Ageing Research Institute - Screened all publications**

https://www.nari.net.au/Pages/Category/research?Take=46

**Google Scholar - Searched using Harzing's Publish or Perish Software**

“older adults” (“friendly visiting” OR ("befriending" "volunteer")) (“loneliness” OR "social isolation")

## 2 Data collection form

| **Characteristics of included studies** | | | | |
| --- | --- | --- | --- | --- |
| Author, year, country | Study design | Population | Comparison | Remarks |
|  |  |  |  |  |
|  |  |  |  |  |

| **Synthesis of findings** | | | | |
| --- | --- | --- | --- | --- |
| Outcome | Comparison | Effect size | # studies, # participants | Reference |
|  |  |  |  |  |
|  |  |  |  |  |

## 3 ROBINS-I assessment

**Arthur 1973**

*Pre-assessment*

| Target randomized trial | Design | Individually randomized |
| --- | --- | --- |
|  | Participants | Nursing home residents |
|  | Intervention | I1: Friendly visiting by the same volunteer each week I2: Friendly visiting by a different volunteer each week |
|  | Comparator | No friendly visiting |
| Aim of the study | | Assess effect of starting and adhering to the intervention |
| Outcome(s) | | Life satisfaction |
| Confounding factors, identified a priori | | Gender, age |
| Confounding factors, additionally identified by study author | | NA |
| Co-interventions, identified a priori | | NA |
| Co-interventions, additionally identified by study author | | NA |

*Assessment*
= same for the different comparisons

| **Outcome** | **Risk of bias domain** | **Signaling question** | **Answer [Y/PY/N/PN/NI]** | **Rationale** | |
| --- | --- | --- | --- | --- | --- |
| Life satisfaction | Confounding | 1.1 | PY | Although very little information is available, the reviewers cannot rule out that the study investigators used certain criteria to allocate the older adults to one or the other group (i.e. used non-random allocation). | |
|  |  | 1.2 | PN |  | |
|  |  | 1.3 | NA |  | |
|  |  | 1.4 | N | No appropriate analysis method reported | |
|  |  | 1.5 | NA |  | |
|  |  | 1.6 | N |  | |
|  |  | 1.7 | NA |  | |
|  |  | 1.8 | NA |  | |
|  | Selection of the participants into the study | 2.1 | N | Selection of participants into the study occurred prior to the start of the intervention | |
|  |  | 2.2 | NA |  | |
|  |  | 2.3 | NA |  | |
|  |  | 2.4 | Y |  | |
|  |  | 2.5 | NA |  | |
|  | Classification of interventions | 3.1 | PY |  | |
|  |  | 3.2 | PY |  | |
|  |  | 3.3 | PN |  | |
|  | Deviations from intended interventions | 4.1 | NA |  | |
|  |  | 4.2 | NA |  | |
|  |  | 4.3 | Y | No co-interventions | |
|  |  | 4.4 | Y | No drop-out occurred, nor did any of the friendly visitors depart (email conversation with Gary Arthur) | |
|  |  | 4.5 | Y |  |  |
|  |  | 4.6 | NA |  | |
|  | Missing data | 5.1 | Y | No drop-out occurred, nor did any of the friendly visitors depart (email conversation with Gary Arthur) | |
|  |  | 5.2 | N |  |  |
|  |  | 5.3 | N |  |  |
|  |  | 5.4 | NA |  | |
|  |  | 5.5 | NA |  | |
|  | Measurement of outcomes | 6.1 | Y | It was impossible to blind the participants and the volunteers who delivered the intervention, as the visiting itself is the intervention. This may have affected the subjective LSI-A scale results due to social desirability bias. | |
|  |  | 6.2 | Y | Outcome assessors were the participants themselves. | |
|  |  | 6.3 | Y |  | |
|  |  | 6.4 | Y |  | |
|  | Selection of the reported result | 7.1 | PN | No indication |  |
|  |  | 7.2 | PN |  |  |
|  |  | 7.3 | N |  |  |

**Bogat 1983**

*Pre-assessment*

| Target randomized trial | Design | Individually randomized |
| --- | --- | --- |
|  | Participants | Community-dwelling older adults |
|  | Intervention | Friendly visiting by a volunteer |
|  | Comparator | No friendly visiting by a volunteer |
| Aim of the study | | Assess effect of starting and adhering to the intervention |
| Outcome(s) | | Number of daily telephone calls, number of visitors and visits made, current networks, life satisfaction |
| Confounding factors, identified a priori | | Age, income, educational level, marital status |
| Confounding factors, additionally identified by study author | | NA |
| Co-interventions, identified a priori | | NA |
| Co-interventions, additionally identified by study author | | NA |

*Assessment*

| **Risk of bias domain** | **Outcome** | **Signaling question** | **Answer [Y/PY/N/PN/NI]** | **Rationale** |
| --- | --- | --- | --- | --- |
| Confounding | All | 1.1 | Y | Random allocation only occurred in the two intervention groups. The control group was not included in the randomization process. |
|  |  | 1.2 | N |  |
|  |  | 1.3 | NA |  |
|  |  | 1.4 | N |  |
|  |  | 1.5 | NA |  |
|  |  | 1.6 | N |  |
|  |  | 1.7 | NA |  |
|  |  | 1.8 | NA |  |
| Selection of the participants into the study |  | 2.1 | N | Selection of participants into the analyses was not related to the outcomes. |
|  |  | 2.2 | NA |  |
|  |  | 2.3 | NA |  |
|  |  | 2.4 | Y |  |
|  |  | 2.5 | NA |  |
| Classification of interventions |  | 3.1 | PY |  |
|  |  | 3.2 | PY |  |
|  |  | 3.3 | PN |  |
| Deviations from intended interventions |  | 4.1 | NA |  |
|  |  | 4.2 | NA |  |
|  |  | 4.3 | Y | No co-interventions |
|  |  | 4.4 | Y | Minimal drop-out (N=4 out of 39, equally distributed among study arms), no switches |
|  |  | 4.5 | Y |  |
|  |  | 4.6 | NA |  |
| Missing data |  | 5.1 | Y | Minimal drop-out (N=4 out of 39, equally distributed among study arms), no switches |
|  |  | 5.2 | N |  |
|  |  | 5.3 | NI |  |
|  |  | 5.4 | NA |  |
|  |  | 5.5 | NA |  |
| Measurement of outcomes | Number of telephone calls, number of visitors and visits | 6.1 | NI | It is unclear if these outcomes were measured via self-reporting or in a more objective way (e.g. via phone provider logs, which involve negligible assessor judgement). |
|  |  | 6.2 | NI | The authors state that in order to control for volunteer expectations and social desirability responses, post-point data for the experimental group were collected by testers unknown to the participants. However, it is unclear how outcomes were measured. |
|  |  | 6.3 | PY |  |
|  |  | 6.4 | PY | Outcomes for the intervention group were collected by the student visitor, whereas those for the control group were collected by a nun. Although outcomes are self-reported, the presence of a student vs a nun might have impacted the effort made by the study person to cooperate or might have led to differential social desirability bias. |
|  | Current networks | 6.1 | PY | This outcome was self-reported (via survey). Responses may be prone to desirability bias. |
|  |  | 6.2 | PY |  |
|  |  | 6.3 | PY |  |
|  |  | 6.4 | PY |  |
|  | Life satisfaction | 6.1 | Y | The LSI-A scale is subjective. |
|  |  | 6.2 | Y | It was impossible to blind the participants who received the intervention, as the visiting itself is the intervention. This may have affected the subjective LSI-A scale results, regardless of who took the tests from the older adults. |
|  |  | 6.3 | PY |  |
|  |  | 6.4 | PY |  |
| Selection of the reported result | All | 7.1 | PN | No indication |
|  |  | 7.2 | PY | Some of the data are reported as post-test means adjusted for pre-test scores, others as mean changes between post- and pre-test scores. |
|  |  | 7.3 | N |  |

**Kahlbaugh 2011**

*Pre-assessment*

| Target randomized trial | Design | Individually randomized |
| --- | --- | --- |
|  | Participants | Older adults residing in independent living appartments |
|  | Intervention | I1: Friendly visiting + playing Wii I2: Friendly visiting + watching TV |
|  | Comparator | No friendly visiting |
| Aim of the study | | Assess effect of starting and adhering to the intervention |
| Outcome(s) | | Loneliness, life satisfaction, positive mood, negative mood |
| Confounding factors, identified a priori | | Age, race, marital status, average health quotient, educational level, physical activity |
| Confounding factors, additionally identified by study author | | NA |
| Co-interventions, identified a priori | | NA |
| Co-interventions, additionally identified by study author | | NA |

*Assessment*

= same for the different comparisons and the different outcomes

| **Risk of bias domain** | **Signaling question** | **Answer [Y/PY/N/PN/NI]** | **Rationale** |
| --- | --- | --- | --- |
| Confounding | 1.1 | Y | Random allocation only occurred in the two intervention groups. The control group was not included in the randomization process. |
|  | 1.2 | N |  |
|  | 1.3 | NA |  |
|  | 1.4 | N |  |
|  | 1.5 | NA |  |
|  | 1.6 | N |  |
|  | 1.7 | NA |  |
|  | 1.8 | NA |  |
| Selection of the participants into the study | 2.1 | N | Selection of participants into the analyses was not related to the outcomes. |
|  | 2.2 | NA |  |
|  | 2.3 | NA |  |
|  | 2.4 | Y |  |
|  | 2.5 | NA |  |
| Classification of interventions | 3.1 | PY |  |
|  | 3.2 | PY |  |
|  | 3.3 | PN |  |
| Deviations from intended interventions | 4.1 | NA |  |
|  | 4.2 | NA |  |
|  | 4.3 | Y | No co-interventions |
|  | 4.4 | Y |  |
|  | 4.5 | Y |  |
|  | 4.6 | NA |  |
| Missing data | 5.1 | Y | Only one older female participant in the television condition, age 86, died during the study period at Week 4 and was excluded from all analyses |
|  | 5.2 | N |  |
|  | 5.3 | NI |  |
|  | 5.4 | NA |  |
|  | 5.5 | NA |  |
| Measurement of outcomes | 6.1 | Y | The UCLA, PANAS and LSI-A scales are subjective. |
|  | 6.2 | Y | It was impossible to blind the participants who received the intervention, as the visiting itself is the intervention. This may have affected the subjective scale results (social desirability bias). |
|  | 6.3 | PY |  |
|  | 6.4 | PY |  |
| Selection of the reported result | 7.1 | PN | No indication |
|  | 7.2 | PN |  |
|  | 7.3 | N |  |

**Mulligan 1978**

*Pre-assessment*

| Target randomized trial | Design | Individually randomized |
| --- | --- | --- |
|  | Participant | Community-dwelling socially isolated older adults |
|  | Intervention | Friendly visiting by a volunteer |
|  | Comparator | No friendly visiting by a volunteer |
| Aim of the study | | Assess effect of starting and adhering to the intervention |
| Outcome(s) | | Social isolation, presence of functional mental disorders |
| Confounding factors, identified a priori | | Age, gender, race, "other demographic factors" |
| Confounding factors, additionally identified by study author | | NA |
| Co-interventions, identified a priori | | NA |
| Co-interventions, additionally identified by study author | | NA |

*Assessment*

| **Risk of bias domain** | **Outcome** | **Signaling question** | **Answer [Y/PY/N/PN/NI]** | **Rationale** |
| --- | --- | --- | --- | --- |
| Confounding | All | 1.1 | Y | Allocation was based on geographic location |
|  |  | 1.2 | PN |  |
|  |  | 1.3 | NA |  |
|  |  | 1.4 | N |  |
|  |  | 1.5 | NA |  |
|  |  | 1.6 | N |  |
|  |  | 1.7 | N |  |
|  |  | 1.8 | NA |  |
| Selection of the participants into the study |  | 2.1 | NI | In both the intervention and control group, the number of participants is different at the first visit (intervention: n=10, control: n=12) than at the last visit (intervention: n=11, control: n=11). The authors do not provide any explanation on this. It is unclear if selection into the analysis is related to the intervention and/or outcome. |
|  |  | 2.2 | NA |  |
|  |  | 2.3 | NA |  |
|  |  | 2.4 | Y |  |
|  |  | 2.5 | NA |  |
| Classification of interventions |  | 3.1 | PY |  |
|  |  | 3.2 | PY |  |
|  |  | 3.3 | PN |  |
| Deviations from intended interventions |  | 4.1 | NA |  |
|  |  | 4.2 | NA |  |
|  |  | 4.3 | Y | No co-interventions |
|  |  | 4.4 | PY |  |
|  |  | 4.5 | PY |  |
|  |  | 4.6 | NA |  |
| Missing data | Short-term social isolation; Functional mental disorders | 5.1 | PY |  |
|  |  | 5.2 | NI |  |
|  |  | 5.3 | NI |  |
|  |  | 5.4 | NA |  |
|  |  | 5.5 | NA |  |
|  | Long-term social isolation | 5.1 | N | Substantial drop-out from last visit to follow-up in both intervention arms: in the friendly visiting group, 8 out of 10 (or 11) participants were found at follow-up; in the control group, only 5 out of 12 (or 11) participants were found at follow-up. |
|  |  | 5.2 | NI |  |
|  |  | 5.3 | NI |  |
|  |  | 5.4 | N | Reasons were similar, but proportions were not. |
|  |  | 5.5 | N | No sensitivity analyses were performed by the investigators, nor by the reviewers. |
| Measurement of outcomes | Social isolation | 6.1 | PY | The Past Month Isolation Index measures the number of face-to-face contacts in the preceding month. The reviewers consider it likely that the responses of the participants are influenced by social desirability bias and recall bias. |
|  |  | 6.2 | Y | It was impossible to blind the participants, who are also the outcome assessors, as the visiting was part of the intervention. |
|  |  | 6.3 | PY |  |
|  |  | 6.4 | Y |  |
|  | Presence of functional mental disorders | 6.1 | PY | The MSS requires assessor judgement. |
|  |  | 6.2 | Y | It was impossible to blind the visitors (as the visiting was part of the intervention) who also filled in the MSS. Although the outcomes were assessed independently by the 2 visiting volunteers, the results may still be biased. |
|  |  | 6.3 | PY |  |
|  |  | 6.4 | Y |  |
| Selection of the reported result | Social isolation | 7.1 | PY | Two scales are mentioned, i.e. the Adulthood Isolation Index and the Past Month Isolation Index. However, only one measure is reported. |
|  |  | 7.2 | PN |  |
|  |  | 7.3 | N |  |
|  | Presence of functional mental disorders | 7.1 | PN |  |
|  |  | 7.2 | PN |  |
|  |  | 7.3 | N |  |

***ROBINS-I summary***

| **Author, Year** | **Comparison** | **Outcome** | **Bias due to confounding** | **Bias in selection of the participants into the study** | **Bias in classification of interventions** | **Bias due to deviations from intended interventions** | **Bias due to missing data** | **Bias in measurement of outcomes** | **Bias in selection of the reported result** | **Overall risk of bias** |
| --- | --- | --- | --- | --- | --- | --- | --- | --- | --- | --- |
| Arthur, 1973 | Same volunteer vs no visiting | Life satisfaction | No information | Low | Low | Low | Low | Serious | Low | Serious |
|  | Different volunteer vs no visiting |  |  |  |  |  |  |  |  |  |
| Bogat, 1983 | Visiting vs no visiting | Number of telephone calls; Number of visitors and visits; Current networks | Serious | Low | Low | Low | Low | Serious | Serious | Serious |
|  |  | Life satisfaction |  |  |  |  |  |  |  |  |
| Kahlbaugh, 2011 | Visit + Wii vs no visit | Loneliness; life satisfaction; positive mood; negative mood | Serious | Low | Low | Low | Low | Serious | Low | Serious |
|  | Visit + TV vs no visit |  |  |  |  |  |  |  |  |  |
| Mulligan 1978 | Visiting vs no visiting | Short-term social isolation | Serious | No information | Low | Low | Low | Serious | Serious | Serious |
|  |  | Long-term social isolation |  |  |  |  | Serious |  |  |  |
|  |  | Functional mental disorders |  |  |  |  | Low |  | Low |  |
